# Supplementary material for: Age-Related Changes in the Matrisome of the Mouse Skeletal Muscle
Source: Int J Mol Sci. 2021 Sep 29;22(19):10564. doi: 10.3390/ijms221910564 (PMC8508832; doi:10.3390/ijms221910564)
Supplement: Supplementary file 1 [file ijms-22-10564-s001.zip › Table S2.pdf]

Table S2. Label free quantification of matrisome proteins identified with at least of two peptides.

PBS extraction				
**Category**	**Protein symbol**	**Log_2 Fold change**	**p-value**	**Protein name**
**ECM regulators**	SPA3K	-0,172	0,4507	Serine protease inhibitor A3K
	SPA3N	1,773	0,0284	Serine protease inhibitor A3N
	A1AT3	0,296	0,2062	Alpha-1-antitrypsin 1-3
	A1AT2	0,693	0,0056	Alpha-1-antitrypsin 1-2 (AAT)
	A1AT4	0,746	0,1332	Alpha-1-antitrypsin 1-4
	PZP	0,863	0,0123	Pregnancy zone protein (Alpha-2-macroglobulin)
	KNG1	0,970	0,0435	Kininogen-1
	ANT3	0,516	0,2097	Antithrombin-III
	A2AP	1,037	0,0382	Alpha-2-antiplasmin
	CBG	1,824	0,0492	Corticosteroid-binding globulin
	ITIH2	2,002	0,0384	Inter-alpha-trypsin inhibitor heavy chain H2
	PLMN	0,403	0,4787	Plasminogen
	ITIH4	1,424	0,0176	Inter alpha-trypsin inhibitor, heavy chain 4
	ILEUA	1,107	0,0083	Leukocyte elastase inhibitor A
	CATD	0,578	0,0008	Cathepsin D
	HEP2	1,277	0,0913	Heparin cofactor 2 (Heparin cofactor II) (HC-II)
	CATB	0,712	0,0783	Cathepsin B
	ITIH3	1,153	0,0598	Inter-alpha-trypsin inhibitor heavy chain H3
	PEDF	1,113	0,0399	Pigment epithelium-derived factor
	HRG	0,484	0,5714	Histidine-rich glycoprotein
	IC1	0,514	0,3235	Plasma protease C1 inhibitor
	THRB	0,530	0,5704	Prothrombin
	AMBP	2,275	0,0785	Protein AMBP
	ITIH1	0,819	0,1858	Inter-alpha-trypsin inhibitor heavy chain H1
	CPN2	0,666	0,4728	Carboxypeptidase N subunit 2
	CYTB	2,805	0,0780	Cystatin-B
	FIBG	1,138	0,0014	Fibrinogen gamma chain

**ECM_Glycoproteins**	FIBB	1,190	0,0015	Fibrinogen beta chain
	FIBA	1,190	0,0009	Fibrinogen alpha chain
	TSP4	1,438	0,0004	Thrombospondin-4
	COMP	0,320	0,7150	Cartilage oligomeric matrix protein
	VMA5A	0,806	0,0002	von Willebrand factor A domain-containing protein 5A
	ADIPO	0,071	0,8364	Adiponectin
**Proteoglycans**	LUM	0,484	0,0304	Lumican
	PRELP	0,373	0,0007	Prolargin
	MIME	0,398	0,1075	Mimecan
	PGS2	0,162	0,1598	Decorin
	ASPN	0,531	0,0036	Asporin
	FMOD	-0,023	0,9634	Fibromodulin
**ECM affiliated**	ANXA6	-0,392	0,0079	Annexin A6
	ANX11	0,281	0,0209	Annexin A11
	ANXA4	0,635	0,0020	Annexin A4
	ANXA3	0,273	0,2470	Annexin A3
	HEMO	0,744	0,0176	Hemopexin
	ANXA5	0,513	0,0085	Annexin A5
	ANXA2	0,263	0,1759	Annexin A2
	LEG1	-0,015	0,9281	Galectin-1
	ANXA1	0,002	0,9973	Annexin A1
	ANXA7	-0,077	0,3791	Annexin A7
	LEGL	-0,956	0,1733	Galectin-related protein
**Secreted factors**	S10A1	0,243	0,3729	Protein S100-A1
	S10A6	0,404	0,0627	Protein S100-A6
**UT extraction**				
**Collagens**	CO6A1	0,614	0,0004	Collagen alpha-1(VI) chain
	CO6A2	0,592	0,0012	Collagen alpha-2(VI) chain
	CO1A2	0,007	0,9864	Collagen alpha-2(I) chain
	CO1A1	-0,228	0,5536	Collagen alpha-1(I) chain

	CO6A6	0,090	0,4178	Collagen alpha-6(VI) chain
	COFA1	0,356	0,3462	Collagen alpha-1(XV) chain
	CO4A1	1,302	0,0163	Collagen alpha-1(IV) chain
**ECM regulators**	SPA3K	0,104	0,8133	Serine protease inhibitor A3K
	A1AT3	0,392	0,1245	Alpha-1-antitrypsin 1-3
	A1AT2	0,868	0,0216	Alpha-1-antitrypsin 1-2
	PZP	0,930	0,1287	Pregnancy zone protein (Alpha-2-macroglobulin)
	KNG1	-0,570	0,8294	Kininogen-1
	ANT3	1,006	0,4191	Antithrombin-III (ATIII) (Serpin C1)
	CATD	1,037	0,0002	Cathepsin D
	CATB	1,054	0,0945	Cathepsin B
	PEDF	-0,528	0,7054	Pigment epithelium-derived factor
	TGM2	0,805	0,0130	Protein-glutamine gamma-glutamyltransferase 2
	SERPH	-0,547	0,1747	Serpin H1
**ECM_Glycoproteins**	FIBG	0,897	0,0872	Fibrinogen gamma chain
	FIBB	0,714	0,1157	Fibrinogen beta chain
	FIBA	0,846	0,2517	Fibrinogen alpha chain
	TSP4	1,338	0,0480	Thrombospondin-4
	COMP	1,533	0,0515	Cartilage oligomeric matrix protein
	LAMA2	0,454	0,0001	Laminin subunit alpha-2
	LAMC1	0,561	0,0000	Laminin subunit gamma-1
	NID1	0,544	0,0000	Nidogen-1
	NID2	1,090	0,0004	Nidogen-2
	LAMB2	0,526	0,0002	Laminin subunit beta-2
	LAMB1	0,470	0,0102	Laminin subunit beta-1
	BGH3	0,775	0,0158	Transforming growth factor-beta-induced protein ig-h3
	POSTN	1,280	0,0034	Periostin
	FINC	1,348	0,0360	Fibronectin
	DERM	0,508	0,0035	Dermatopontin
	LAMA5	0,790	0,0019	Laminin subunit alpha-5

	CILP1	1,468	0,0001	Cartilage intermediate layer protein 1
	LAMA4	1,452	0,1224	Laminin subunit alpha-4
	FBN1	0,385	0,4600	Fibrillin-1
	MFAP4	1,162	0,6121	Microfibril-associated glycoprotein 4
	TENA	2,547	0,0219	Tenascin
	VWA1	3,966	0,1050	von Willebrand factor A domain-containing protein 1
**Proteoglycans**	LUM	0,806	0,0010	Lumican
	PRELP	0,865	0,0071	Prolargin
	MIME	0,543	0,0084	Mimecan
	PGS2	0,437	0,0540	Decorin
	ASPN	0,791	0,0079	Asporin
	FMOD	0,728	0,0053	Fibromodulin
	PGS1	1,048	0,0024	Biglycan
	PGBM	0,133	0,0879	Basement membrane-specific heparan sulfate proteoglycan core protein
**ECM affiliated**	ANXA6	0,260	0,1341	Annexin A6
	ANX11	0,904	0,0000	Annexin A11
	HEMO	1,593	0,0074	Hemopexin
	ANXA5	2,283	0,0018	Annexin A5
	ANXA2	0,420	0,0046	Annexin A2
	LEG1	1,191	0,0209	Galectin-1
	GPC1	0,835	0,1576	Glypican-1
	LMAN1	0,517	0,7165	Protein ERGIC-53
**Secreted factors**	ANGL7	1,140	0,0763	Angiopoietin-related protein 7
	S10AA	0,764	0,4253	Protein S100-A10
**Gu extraction**				
	CO6A1	0,126	0,4160	Collagen alpha-1(VI) chain
	CO6A2	0,216	0,1564	Collagen alpha-2(VI) chain
	CO1A2	-0,114	0,8601	Collagen alpha-2(I) chain
	CO1A1	-0,205	0,7748	Collagen alpha-1(I) chain
	CO6A6	0,706	0,3562	Collagen alpha-6(VI) chain

**Collagens**	COFA1	0,672	0,0191	Collagen alpha-1(XV) chain
	CO4A1	0,694	0,0984	Collagen alpha-1(IV) chain
	CO4A2	0,501	0,2004	Collagen alpha-2(IV) chain
	CO3A1	1,231	0,4124	Collagen alpha-1(III) chain
	CO5A1	-0,257	0,5889	Collagen alpha-1(V) chain
	CO5A2	-0,390	0,7073	Collagen alpha-2(V) chain
**ECM regulators**	SPA3K	-0,417	0,4127	Serine protease inhibitor A3K
	A1AT3	0,084	0,8121	Alpha-1-antitrypsin 1-3
	ITIH3	-0,007	0,9848	Inter-alpha-trypsin inhibitor heavy chain H3
	ITIH1	0,210	0,8696	Inter-alpha-trypsin inhibitor heavy chain H1
	TGM2	0,427	0,0127	Protein-glutamine gamma-glutamyltransferase 2
	ITIH5	0,995	0,2585	Inter-alpha-trypsin inhibitor heavy chain H5
**ECM_Glycoproteins**	FIBG	-0,182	0,8531	Fibrinogen gamma chain
	FIBB	0,779	0,3330	Fibrinogen beta chain
	FIBA	-0,874	0,1989	Fibrinogen alpha chain
	TSP4	0,667	0,2280	Thrombospondin-4
	COMP	0,446	0,4315	Cartilage oligomeric matrix protein
	ADIPO	2,916	0,0532	Adiponectin
	LAMA2	0,373	0,0280	Laminin subunit alpha-2
	LAMC1	0,355	0,0124	Laminin subunit gamma-1
	NID1	0,235	0,0452	Nidogen-1
	NID2	0,457	0,4769	Nidogen-2
	LAMB2	0,386	0,0570	Laminin subunit beta-2
	LAMB1	0,434	0,0049	Laminin subunit beta-1
	BGH3	-0,141	0,7957	Transforming growth factor-beta-induced protein ig-h3
	FINC	0,563	0,4324	Fibronectin
	DERM	0,374	0,1918	Dermatopontin
	LAMA5	0,810	0,0065	Laminin subunit alpha-5
	CILP1	0,620	0,1852	Cartilage intermediate layer protein 1
	FBN1	0,272	0,5764	Fibrillin-1

	TENA	-0,319	0,5716	Tenascin
	CILP2	0,250	0,6497	Cartilage intermediate layer protein 2
**Proteoglycans**	LUM	0,314	0,2667	Lumican
	PRELP	0,515	0,3634	Prolargin
	MIME	0,196	0,4148	Mimecan
	PGS2	0,043	0,9052	Decorin
	ASPN	0,535	0,1137	Asporin
	FMOD	-0,171	0,5134	Fibromodulin
	PGS1	0,641	0,1162	Biglycan
	PGBM	0,552	0,0599	Basement membrane-specific heparan sulfate proteoglycan core protein
	PRG2	2,860	0,0796	Bone marrow proteoglycan
**ECM affiliated**	ANXA6	0,326	0,3756	Annexin A6
	ANX11	0,966	0,0745	Annexin A11
	HEMO	0,573	0,2109	Hemopexin
	ANXA2	0,670	0,0460	Annexin A2
	LEG1	0,323	0,3258	Galectin-1
**Secreted factors**	ANGL7	0,375	0,7501	Angiopoietin-related protein 7
