# Supplementary material for: Age-Related Changes in the Matrisome of the Mouse Skeletal Muscle
Source: Int J Mol Sci. 2021 Sep 29;22(19):10564. doi: 10.3390/ijms221910564 (PMC8508832; doi:10.3390/ijms221910564)
Supplement: Supplementary file 1 [file ijms-22-10564-s001.zip › Table S1.pdf]

Table S1. List of identified proteins in each fraction. The protein library was built by LC-MS/MS raw data of gastrocnemius muscle from adult and old mice.

PBS extract						
**Protein symbol**	**Score**	**Mass**	**Num. of significant matches**	**Num. of significant sequences**	**Num. of significant unique sequences**	**Protein name**
1433B_MOUSE	3708	28183	199	13	6	14-3-3 protein beta/alpha
1433E_MOUSE	11008	29326	481	18	15	14-3-3 protein epsilon
1433F_MOUSE	2260	28365	128	9	4	14-3-3 protein eta
1433G_MOUSE	9375	28456	441	15	9	14-3-3 protein gamma
1433T_MOUSE	2119	28046	102	8	3	14-3-3 protein theta
1433Z_MOUSE	4793	27925	233	14	9	14-3-3 protein zeta/delta
2A5E_MOUSE	32	55078	1	1	1	Serine/threonine-protein phosphatase 2A 56 kDa regulatory subunit epsilon isoform
2AAA_MOUSE	6254	66079	341	24	24	Serine/threonine-protein phosphatase 2A 65 kDa regulatory subunit A alpha isoform
2ABA_MOUSE	1209	52173	71	7	7	Serine/threonine-protein phosphatase 2A 55 kDa regulatory subunit B alpha isoform
3HIDH_MOUSE	907	35816	43	5	5	3-hydroxyisobutyrate dehydrogenase, mitochondrial
5NT1A_MOUSE	32	40824	2	2	2	Cytosolic 5'-nucleotidase 1A
5NT3A_MOUSE	1035	37571	90	11	11	Cytosolic 5'-nucleotidase 3A
6PGD_MOUSE	1723	53726	95	13	13	6-phosphogluconate dehydrogenase, decarboxylating
6PGL_MOUSE	1205	27465	81	9	9	6-phosphogluconolactonase
A16A1_MOUSE	20	85501	2	1	1	Aldehyde dehydrogenase family 16 member A1
A1AG1_MOUSE	794	23994	24	3	3	Alpha-1-acid glycoprotein 1
A1AT1_MOUSE	15902	46145	648	14	1	Alpha-1-antitrypsin 1-1
A1AT2_MOUSE	15185	46117	644	13	4	Alpha-1-antitrypsin 1-2
A1AT3_MOUSE	17308	45966	727	14	1	Alpha-1-antitrypsin 1-3
A1AT4_MOUSE	13826	46140	615	12	2	Alpha-1-antitrypsin 1-4
A2AP_MOUSE	1464	55165	88	10	10	Alpha-2-antiplasmin
AAKB2_MOUSE	69	30304	7	3	3	5'-AMP-activated protein kinase subunit beta-2
AAKG1_MOUSE	553	37668	23	3	3	5'-AMP-activated protein kinase subunit gamma-1
AAKG3_MOUSE	252	54271	18	3	3	5'-AMP-activated protein kinase subunit gamma-3
AAMDC_MOUSE	735	13350	35	3	3	Mth938 domain-containing protein
AAPK2_MOUSE	109	62610	16	5	5	5'-AMP-activated protein kinase catalytic subunit alpha-2
AATC_MOUSE	28473	46504	1189	28	28	Aspartate aminotransferase, cytoplasmic
AATM_MOUSE	30635	47780	1383	29	29	Aspartate aminotransferase, mitochondrial
ABCA2_MOUSE	20	272788	1	1	1	ATP-binding cassette sub-family A member 2
ABCB8_MOUSE	30	78691	1	1	1	ATP-binding cassette sub-family B member 8, mitochondrial
ABCE1_MOUSE	132	68240	12	5	5	ATP-binding cassette sub-family E member 1
ABEC2_MOUSE	3409	25872	175	10	10	C->U-editing enzyme APOBEC-2
ABHDB_MOUSE	362	33654	23	4	4	Protein ABHD11
ABHDD_MOUSE	45	38785	6	1	1	Protein ABHD13
ABRA_MOUSE	108	42976	12	1	1	Actin-binding Rho-activating protein
ACACB_MOUSE	32	277969	2	2	2	Acetyl-CoA carboxylase 2
ACAD8_MOUSE	124	45789	7	5	5	Isobutyryl-CoA dehydrogenase, mitochondrial
ACAD9_MOUSE	406	69192	30	3	3	Acyl-CoA dehydrogenase family member 9, mitochondrial
ACADL_MOUSE	13195	48277	708	23	23	Long-chain specific acyl-CoA dehydrogenase, mitochondrial
ACADM_MOUSE	5888	46908	309	16	16	Medium-chain specific acyl-CoA dehydrogenase, mitochondrial
ACADS_MOUSE	2455	45146	162	15	15	Short-chain specific acyl-CoA dehydrogenase, mitochondrial
ACADV_MOUSE	11086	71230	524	32	32	Very long-chain specific acyl-CoA dehydrogenase, mitochondrial
ACBP_MOUSE	478	9994	40	3	3	Acyl-CoA-binding protein
ACDSB_MOUSE	1586	48300	55	7	7	Short/branched chain specific acyl-CoA dehydrogenase, mitochondrial
ACLY_MOUSE	226	120564	9	3	3	ATP-citrate synthase
ACO13_MOUSE	382	15287	22	3	3	Acyl-coenzyme A thioesterase 13
ACOC_MOUSE	207	98691	24	7	7	Cytoplasmic aconitate hydratase
ACON_MOUSE	52991	86151	2158	43	43	Aconitate hydratase, mitochondrial
ACOT2_MOUSE	1447	49854	94	14	14	Acyl-coenzyme A thioesterase 2, mitochondrial
ACOT9_MOUSE	23	50813	3	3	3	Acyl-coenzyme A thioesterase 9, mitochondrial
ACS2L_MOUSE	20	75317	1	1	1	Acetyl-coenzyme A synthetase 2-like, mitochondrial
ACSL1_MOUSE	99	78928	9	5	5	Long-chain-fatty-acid--CoA ligase 1
ACTB_MOUSE	16693	42052	839	20	8	Actin, cytoplasmic 1
ACTN2_MOUSE	394	104339	18	5	2	Alpha-actinin-2
ACTN3_MOUSE	580	103605	31	7	4	Alpha-actinin-3
ACTS_MOUSE	36202	42366	1524	27	15	Actin, alpha skeletal muscle
ACTY_MOUSE	275	42369	25	2	2	Beta-centractin
ACY2_MOUSE	79	35778	3	1	1	Aspartoacylase
ACYP1_MOUSE	20	11234	1	1	1	Acylphosphatase-1
ACYP2_MOUSE	1050	11927	71	6	6	Acylphosphatase-2
ADGB_MOUSE	36	189464	8	2	2	Androglobin
ADH1_MOUSE	1392	40601	102	10	10	Alcohol dehydrogenase 1
ADHX_MOUSE	3358	40320	212	15	15	Alcohol dehydrogenase class-3
ADIPO_MOUSE	62	26906	7	2	2	Adiponectin
ADK_MOUSE	398	40466	45	9	9	Adenosine kinase
ADNP_MOUSE	28	125769	4	1	1	Activity-dependent neuroprotector homeobox protein
ADPRH_MOUSE	27	40328	2	2	2	[Protein ADP-ribosylarginine] hydrolase
ADT1_MOUSE	6507	33111	352	19	19	ADP/ATP translocase 1
AF1L2_MOUSE	35	92802	7	2	2	Actin filament-associated protein 1-like 2
AFAM_MOUSE	65	71272	7	4	4	Afamin
AGM1_MOUSE	19	60043	1	1	1	Phosphoacetylglucosamine mutase
AGRIN_MOUSE	25	215386	1	1	1	Agrin
AHSA1_MOUSE	576	38321	28	4	4	Activator of 90 kDa heat shock protein ATPase homolog 1
AIMP1_MOUSE	22	34318	1	1	1	Aminoacyl tRNA synthase complex-interacting multifunctional protein 1
AIMP2_MOUSE	161	35640	13	2	2	Aminoacyl tRNA synthase complex-interacting multifunctional protein 2
AK1A1_MOUSE	695	36792	55	11	11	Alcohol dehydrogenase [NADP(+)]
AKT2_MOUSE	709	56105	21	3	3	RAC-beta serine/threonine-protein kinase
AL1A1_MOUSE	1181	55060	79	10	9	Retinal dehydrogenase 1

AL1L1_MOUSE	21	99502	1	1	1	Cytosolic 10-formyltetrahydrofolate dehydrogenase
AL3A1_MOUSE	1834	50848	102	14	14	Aldehyde dehydrogenase, dimeric NADP-preferring
AL4A1_MOUSE	986	62258	69	12	12	Delta-1-pyrroline-5-carboxylate dehydrogenase, mitochondrial
AL7A1_MOUSE	32	59337	2	1	1	Alpha-aminoadipic semialdehyde dehydrogenase
ALAT1_MOUSE	135	55905	22	4	4	Alanine aminotransferase 1
ALBU_MOUSE	114385	70700	4158	55	52	Serum albumin
ALD1_MOUSE	772	36137	61	6	4	Aldose reductase-related protein 1
ALD2_MOUSE	742	36440	50	6	4	Aldose reductase-related protein 2
ALDH2_MOUSE	7243	57015	400	23	22	Aldehyde dehydrogenase, mitochondrial
ALDOA_MOUSE	213115	39787	9549	42	7	Fructose-bisphosphate aldolase A
ALDR_MOUSE	12532	36052	588	17	17	Aldose reductase
AMBP_MOUSE	84	39916	7	2	2	Protein AMBP
AMPB_MOUSE	19	73054	1	1	1	Aminopeptidase B
AMPD1_MOUSE	7317	86734	470	36	36	AMP deaminase 1
AMPL_MOUSE	989	56505	88	12	12	Cytosol aminopeptidase
AMRP_MOUSE	20	42189	1	1	1	Alpha-2-macroglobulin receptor-associated protein
AN32A_MOUSE	151	28691	13	3	2	Acidic leucine-rich nuclear phosphoprotein 32 family member A
AN32B_MOUSE	168	31231	13	3	2	Acidic leucine-rich nuclear phosphoprotein 32 family member B
AN32E_MOUSE	467	29832	18	1	1	Acidic leucine-rich nuclear phosphoprotein 32 family member E
ANGT_MOUSE	32	52243	1	1	1	Angiotensinogen
ANKR2_MOUSE	3195	36855	140	13	13	Ankyrin repeat domain-containing protein 2
ANM1_MOUSE	128	43035	13	3	3	Protein arginine N-methyltransferase 1
ANM5_MOUSE	56	73318	6	1	1	Protein arginine N-methyltransferase 5
ANT3_MOUSE	2058	52484	145	18	18	Antithrombin-III
ANX11_MOUSE	5017	54387	243	14	14	Annexin A11
ANXA1_MOUSE	1394	38995	39	4	4	Annexin A1
ANXA2_MOUSE	6474	38937	279	17	17	Annexin A2
ANXA3_MOUSE	742	36533	55	4	4	Annexin A3
ANXA4_MOUSE	1261	36178	78	9	8	Annexin A4
ANXA5_MOUSE	8130	35787	297	17	17	Annexin A5
ANXA6_MOUSE	21148	76294	966	42	41	Annexin A6
ANXA7_MOUSE	935	50178	93	12	12	Annexin A7
AP2A2_MOUSE	118	104863	11	2	2	AP-2 complex subunit alpha-2
AP2B1_MOUSE	78	105428	6	3	3	AP-2 complex subunit beta
AP2S1_MOUSE	20	17178	1	1	1	AP-2 complex subunit sigma
AP4A_MOUSE	357	17150	31	6	6	Bis(5'-nucleosyl)-tetraphosphatase [asymmetrical]
APEH_MOUSE	266	82441	28	6	6	Acylamino-acid-releasing enzyme
APOA1_MOUSE	8350	30597	417	17	17	Apolipoprotein A-I
APOA2_MOUSE	185	11359	16	1	1	Apolipoprotein A-II
APOA4_MOUSE	1324	45001	85	10	10	Apolipoprotein A-IV
APOC3_MOUSE	243	10975	11	1	1	Apolipoprotein C-III
APOD_MOUSE	63	21744	3	1	1	Apolipoprotein D
APOE_MOUSE	621	35901	46	7	7	Apolipoprotein E
APOH_MOUSE	341	39904	36	5	5	Beta-2-glycoprotein 1
APT_MOUSE	1530	19883	61	5	5	Adenine phosphoribosyltransferase
ARC1A_MOUSE	48	42170	6	3	2	Actin-related protein 2/3 complex subunit 1A
ARC1B_MOUSE	56	41836	5	2	1	Actin-related protein 2/3 complex subunit 1B
ARF1_MOUSE	2191	20741	117	6	4	ADP-ribosylation factor 1
ARF4_MOUSE	1164	20498	61	3	1	ADP-ribosylation factor 4
ARHL1_MOUSE	329	40373	20	3	3	[Protein ADP-ribosylarginine] hydrolase-like protein 1
ARK72_MOUSE	590	41042	40	6	6	Aflatoxin B1 aldehyde reductase member 2
ARL3_MOUSE	127	20645	8	2	2	ADP-ribosylation factor-like protein 3
ARLY_MOUSE	37	51878	3	1	1	Argininosuccinate lyase
ARMT1_MOUSE	196	51087	18	5	5	Protein-glutamate O-methyltransferase
ARP2_MOUSE	152	45017	11	3	3	Actin-related protein 2
ARP3_MOUSE	673	47783	30	3	3	Actin-related protein 3
ARPC2_MOUSE	80	34450	7	3	3	Actin-related protein 2/3 complex subunit 2
ARPC3_MOUSE	97	20739	9	1	1	Actin-related protein 2/3 complex subunit 3
ARPC4_MOUSE	68	19768	8	4	4	Actin-related protein 2/3 complex subunit 4
ARXS1_MOUSE	43	20191	3	1	1	Adipocyte-related X-chromosome expressed sequence 1
AS3MT_MOUSE	948	42451	48	9	9	Arsenite methyltransferase
ASGL1_MOUSE	837	34385	67	6	6	Isoaspartyl peptidase/L-asparaginase
ASPC1_MOUSE	38	60044	3	1	1	Tether containing UBX domain for GLUT4
ASPN_MOUSE	250	42944	25	6	6	Asporin
ASPP1_MOUSE	20	119667	1	1	1	Apoptosis-stimulating of p53 protein 1
ASSY_MOUSE	62	46840	3	1	1	Argininosuccinate synthase
AT1A2_MOUSE	132	113457	13	6	6	Sodium/potassium-transporting ATPase subunit alpha-2
AT1B4_MOUSE	19	41716	1	1	1	Protein ATP1B4
AT2A1_MOUSE	67489	110723	2529	63	63	Sarcoplasmic/endoplasmic reticulum calcium ATPase 1
AT2L2_MOUSE	384	52333	19	3	3	5-phosphohydroxy-L-lysine phospho-lyase
AT5F1_MOUSE	252	29044	22	5	5	ATP synthase F(0) complex subunit B1, mitochondrial
ATOX1_MOUSE	63	7505	6	3	3	Copper transport protein ATOX1
ATP5E_MOUSE	47	5891	4	1	1	ATP synthase subunit epsilon, mitochondrial
ATP5H_MOUSE	153	18795	15	3	3	ATP synthase subunit d, mitochondrial
ATP5I_MOUSE	156	8230	13	3	3	ATP synthase subunit e, mitochondrial
ATP5J_MOUSE	27	12489	1	1	1	ATP synthase-coupling factor 6, mitochondrial
ATP5L_MOUSE	33	11417	2	2	2	ATP synthase subunit g, mitochondrial
ATPA_MOUSE	26333	59830	937	30	30	ATP synthase subunit alpha, mitochondrial
ATPB_MOUSE	33582	56265	1276	28	28	ATP synthase subunit beta, mitochondrial
ATPD_MOUSE	117	17589	5	1	1	ATP synthase subunit delta, mitochondrial
ATPG_MOUSE	1063	32979	83	8	8	ATP synthase subunit gamma, mitochondrial
ATPK_MOUSE	208	10394	22	1	1	ATP synthase subunit f, mitochondrial
ATPO_MOUSE	573	23406	26	4	4	ATP synthase subunit O, mitochondrial

AUHM_MOUSE	119	33659	12	3	3	Methylglutaconyl-CoA hydratase, mitochondrial
B2L13_MOUSE	387	46862	13	2	2	Bcl-2-like protein 13
BABA2_MOUSE	21	43973	1	1	1	BRISC and BRCA1-A complex member 2
BAF_MOUSE	32	10324	3	2	2	Barrier-to-autointegration factor
BAG3_MOUSE	25	62050	2	1	1	BAG family molecular chaperone regulator 3
BBX_MOUSE	17	101746	1	1	1	HMG box transcription factor BBX
BCAT2_MOUSE	898	44669	72	7	7	Branched-chain-amino-acid aminotransferase, mitochondrial
BFSP1_MOUSE	61	73966	6	1	1	Filensin
BIEA_MOUSE	146	33675	14	1	1	Biliverdin reductase A
BIN1_MOUSE	9458	64658	446	17	17	Myc box-dependent-interacting protein 1
BIP_MOUSE	4709	72492	241	22	20	Endoplasmic reticulum chaperone BiP
BLMH_MOUSE	44	53104	7	2	2	Bleomycin hydrolase
BLVRB_MOUSE	1371	22297	59	4	4	Flavin reductase (NADPH)
BMPR2_MOUSE	22	116259	1	1	1	Bone morphogenetic protein receptor type-2
BPHL_MOUSE	71	33058	8	4	4	Valacyclovir hydrolase
BPNT1_MOUSE	27	33517	3	2	2	3'(2'),5'-bisphosphate nucleotidase 1
BRNP1_MOUSE	20	89896	1	1	1	BMP/retinoic acid-inducible neural-specific protein 1
BTF3_MOUSE	52	22017	2	1	1	Transcription factor BTF3
BZW2_MOUSE	245	48261	32	2	2	Basic leucine zipper and W2 domain-containing protein 2
C1QBP_MOUSE	580	31336	36	3	3	Complement component 1 Q subcomponent-binding protein, mitochondrial
C1TC_MOUSE	1531	101820	97	14	14	C-1-tetrahydrofolate synthase, cytoplasmic
C4BPA_MOUSE	91	52973	7	3	3	C4b-binding protein
CA2D1_MOUSE	52	125692	2	2	2	Voltage-dependent calcium channel subunit alpha-2/delta-1
CAB39_MOUSE	1112	39989	67	6	6	Calcium-binding protein 39
CACB1_MOUSE	52	65731	4	2	2	Voltage-dependent L-type calcium channel subunit beta-1
CACP_MOUSE	4342	71250	289	21	21	Carnitine O-acetyltransferase
CAD13_MOUSE	60	78536	6	3	3	Cadherin-13
CAF17_MOUSE	37	38774	2	1	1	Putative transferase CAF17 homolog, mitochondrial
CAH1_MOUSE	42	28370	2	2	2	Carbonic anhydrase 1
CAH2_MOUSE	1180	29129	78	7	7	Carbonic anhydrase 2
CAH3_MOUSE	29170	29633	1513	23	23	Carbonic anhydrase 3
CALM1_MOUSE	3104	16827	171	10	9	Calmodulin-1
CALR_MOUSE	645	48136	72	7	7	Calreticulin
CALU_MOUSE	126	37155	19	3	3	Calumenin
CAN1_MOUSE	684	82739	55	13	13	Calpain-1 catalytic subunit
CAN2_MOUSE	376	80677	43	11	11	Calpain-2 catalytic subunit
CANB1_MOUSE	22	19402	1	1	1	Calcineurin subunit B type 1
CAND1_MOUSE	211	137955	14	4	4	Cullin-associated NEDD8-dissociated protein 1
CAND2_MOUSE	4720	137086	288	31	31	Cullin-associated NEDD8-dissociated protein 2
CAP1_MOUSE	387	51875	20	3	2	Adenylyl cyclase-associated protein 1
CAP2_MOUSE	43	53114	5	3	2	Adenylyl cyclase-associated protein 2
CAPG_MOUSE	79	39501	6	1	1	Macrophage-capping protein
CAPZB_MOUSE	33	31611	1	1	1	F-actin-capping protein subunit beta
CARME_MOUSE	496	46822	71	7	7	Carnosine N-methyltransferase
CASQ1_MOUSE	18812	46349	837	17	17	Calsequestrin-1
CASQ2_MOUSE	475	48203	19	1	1	Calsequestrin-2
CATA_MOUSE	400	60043	34	9	7	Catalase
CATB_MOUSE	290	38168	35	5	5	Cathepsin B
CATD_MOUSE	388	45381	33	9	9	Cathepsin D
CAVN1_MOUSE	2268	43927	113	8	8	Caveolae-associated protein 1
CAVN4_MOUSE	57	41040	4	4	4	Caveolae-associated protein 4
CAZA2_MOUSE	110	33118	7	2	2	F-actin-capping protein subunit alpha-2
CBG_MOUSE	1083	44911	54	10	10	Corticosteroid-binding globulin
CBPN_MOUSE	31	52098	1	1	1	Carboxypeptidase N catalytic chain
CBPQ_MOUSE	43	51837	2	1	1	Carboxypeptidase Q
CBR1_MOUSE	1340	30907	130	10	9	Carbonyl reductase [NADPH] 1
CBR2_MOUSE	1117	26056	66	8	8	Carbonyl reductase [NADPH] 2
CBR3_MOUSE	146	31333	18	2	1	Carbonyl reductase [NADPH] 3
CBR4_MOUSE	30	25570	3	1	1	Carbonyl reductase family member 4
CBWD1_MOUSE	65	44201	2	1	1	COBW domain-containing protein 1
CC169_MOUSE	124	24739	17	1	1	Coiled-coil domain-containing protein 169
CCD42_MOUSE	34	37965	1	1	1	Coiled-coil domain-containing protein 42
CD158_MOUSE	28	127318	2	1	1	Coiled-coil domain-containing protein 158
CD36_MOUSE	17	53234	1	1	1	Platelet glycoprotein 4
CDC42_MOUSE	358	21587	37	5	4	Cell division control protein 42 homolog
CDC6_MOUSE	18	63658	1	1	1	Cell division control protein 6 homolog
CDNF_MOUSE	60	21645	4	2	2	Cerebral dopamine neurotrophic factor
CDV3_MOUSE	28	29711	1	1	1	Protein CDV3
CE295_MOUSE	33	273923	2	1	1	Centrosomal protein of 295 kDa
CELR3_MOUSE	49	363043	4	1	1	Cadherin EGF LAG seven-pass G-type receptor 3
CERU_MOUSE	5028	121872	265	45	45	Ceruloplasmin
CES1D_MOUSE	822	62034	76	5	2	Carboxylesterase 1D
CFAB_MOUSE	463	86319	40	9	9	Complement factor B
CFAH_MOUSE	38	143722	2	2	2	Complement factor H
CFAI_MOUSE	65	69497	5	3	3	Complement factor I
CH082_MOUSE	102	24656	7	2	2	UPF0598 protein C8orf82 homolog
CH10_MOUSE	2484	10956	133	7	7	10 kDa heat shock protein, mitochondrial
CH60_MOUSE	13405	61088	522	25	25	60 kDa heat shock protein, mitochondrial
CHCH2_MOUSE	52	15879	5	2	2	Coiled-coil-helix-coiled-coil-helix domain-containing protein 2
CIP4_MOUSE	39	68675	1	1	1	Cdc42-interacting protein 4
CISD1_MOUSE	48	12260	2	2	2	CDGSH iron-sulfur domain-containing protein 1
CISY_MOUSE	17653	51988	829	21	21	Citrate synthase, mitochondrial
CKP2L_MOUSE	32	83689	1	1	1	Cytoskeleton-associated protein 2-like

CLAP2_MOUSE	42	141564	4	1	1	CLIP-associating protein 2
CLH1_MOUSE	2319	193202	140	20	20	Clathrin heavy chain 1
CLIC4_MOUSE	47	28939	4	2	2	Chloride intracellular channel protein 4
CLIP1_MOUSE	211	156345	11	7	7	CAP-Gly domain-containing linker protein 1
CLUS_MOUSE	393	52250	23	8	8	Clusterin
CLYBL_MOUSE	73	37867	10	7	7	Citramalyl-CoA lyase, mitochondrial
CMBL_MOUSE	2944	28226	118	8	8	Carboxymethylenebutenolidase homolog
CMC1_MOUSE	1368	74922	86	15	15	Calcium-binding mitochondrial carrier protein Aralar1
CMYA5_MOUSE	97	414327	8	3	3	Cardiomyopathy-associated protein 5
CNBP_MOUSE	144	20833	10	3	3	Cellular nucleic acid-binding protein
CNDP2_MOUSE	360	53190	29	8	8	Cytosolic non-specific dipeptidase
CNPY2_MOUSE	33	21096	1	1	1	Protein canopy homolog 2
CO3_MOUSE	5606	187905	423	53	53	Complement C3
CO4B_MOUSE	335	194447	29	13	13	Complement C4-B
CO5_MOUSE	86	190469	3	1	1	Complement C5
CO6A1_MOUSE	29	109562	2	1	1	Collagen alpha-1(VI) chain
CO8G_MOUSE	19	22665	1	1	1	Complement component C8 gamma chain
CO9_MOUSE	228	63217	9	3	3	Complement component C9
COF1_MOUSE	1690	18776	93	7	5	Cofilin-1
COF2_MOUSE	6546	18812	397	13	11	Cofilin-2
COMP_MOUSE	56	84912	10	4	2	Cartilage oligomeric matrix protein
COMT_MOUSE	19	29695	1	1	1	Catechol O-methyltransferase
COPB_MOUSE	21	108138	1	1	1	Coatomer subunit beta
COPG1_MOUSE	81	98705	2	1	1	Coatomer subunit gamma-1
COQ6_MOUSE	121	51645	5	1	1	Ubiquinone biosynthesis monooxygenase COQ6, mitochondrial
COQ7_MOUSE	19	24197	1	1	1	5-demethoxyubiquinone hydroxylase, mitochondrial
COQ8A_MOUSE	3794	72154	175	13	13	Atypical kinase COQ8A, mitochondrial
COQ9_MOUSE	1996	35232	113	6	6	Ubiquinone biosynthesis protein COQ9, mitochondrial
COTL1_MOUSE	229	16048	10	1	1	Coactosin-like protein
COX2_MOUSE	1481	26130	87	3	3	Cytochrome c oxidase subunit 2
COX41_MOUSE	257	19575	23	6	6	Cytochrome c oxidase subunit 4 isoform 1, mitochondrial
COX5A_MOUSE	377	16319	12	1	1	Cytochrome c oxidase subunit 5A, mitochondrial
COX5B_MOUSE	44	14089	4	2	2	Cytochrome c oxidase subunit 5B, mitochondrial
COX6C_MOUSE	21	8464	1	1	1	Cytochrome c oxidase subunit 6C
COX7C_MOUSE	25	7328	1	1	1	Cytochrome c oxidase subunit 7C, mitochondrial
CP2J5_MOUSE	30	58032	1	1	1	Cytochrome P450 2J5
CPN2_MOUSE	62	61296	5	2	2	Carboxypeptidase N subunit 2
CPNE1_MOUSE	27	59591	2	2	2	Copine-1
CPNE2_MOUSE	114	61681	11	3	3	Copine-2
CPNS1_MOUSE	68	28559	4	2	2	Calpain small subunit 1
CPPED_MOUSE	263	35568	31	4	4	Serine/threonine-protein phosphatase CPPED1
CPT1B_MOUSE	410	89073	18	3	3	Carnitine O-palmitoyltransferase 1, muscle isoform
CPT2_MOUSE	757	74504	54	12	12	Carnitine O-palmitoyltransferase 2, mitochondrial
CQ10B_MOUSE	32	27764	4	1	1	Coenzyme Q-binding protein COQ10 homolog B, mitochondrial
CREG1_MOUSE	66	24550	3	1	1	Protein CREG1
CRK_MOUSE	117	33851	7	1	1	Adapter molecule crk
CRKL_MOUSE	21	33923	1	1	1	Crk-like protein
CRYAB_MOUSE	6100	20056	315	12	12	Alpha-crystallin B chain
CSK21_MOUSE	20	45219	1	1	1	Casein kinase II subunit alpha
CSN1_MOUSE	376	53978	24	4	4	COP9 signalosome complex subunit 1
CSN2_MOUSE	246	51849	23	6	6	COP9 signalosome complex subunit 2
CSN3_MOUSE	162	48371	14	3	3	COP9 signalosome complex subunit 3
CSN4_MOUSE	574	46541	38	7	7	COP9 signalosome complex subunit 4
CSN5_MOUSE	59	37753	4	1	1	COP9 signalosome complex subunit 5
CSN6_MOUSE	171	36142	12	3	3	COP9 signalosome complex subunit 6
CSN7A_MOUSE	536	30377	22	4	4	COP9 signalosome complex subunit 7a
CSN8_MOUSE	58	23298	4	2	2	COP9 signalosome complex subunit 8
CSRP3_MOUSE	622	21793	31	6	6	Cysteine and glycine-rich protein 3
CTBP1_MOUSE	73	48171	4	1	1	C-terminal-binding protein 1
CTL2_MOUSE	16	81539	1	1	1	Choline transporter-like protein 2
CUBN_MOUSE	31	407680	2	1	1	Cubilin
CUL2_MOUSE	110	87448	6	2	2	Cullin-2
CUL4A_MOUSE	42	88211	4	1	1	Cullin-4A
CUL5_MOUSE	425	91486	25	5	5	Cullin-5
CUTA_MOUSE	35	18967	3	2	2	Protein CutA
CUTC_MOUSE	66	29384	3	1	1	Copper homeostasis protein cutC homolog
CX6B1_MOUSE	362	10293	24	3	3	Cytochrome c oxidase subunit 6B1
CX7A1_MOUSE	76	9095	4	1	1	Cytochrome c oxidase subunit 7A1, mitochondrial
CY1_MOUSE	541	35533	38	4	4	Cytochrome c1, heme protein, mitochondrial
CYC_MOUSE	1507	11712	90	6	6	Cytochrome c, somatic
CYGB_MOUSE	70	21566	8	1	1	Cytoglobin
CYTB_MOUSE	30	11153	2	1	1	Cystatin-B
D39U1_MOUSE	69	33145	5	1	1	Epimerase family protein SDR39U1
DAB2_MOUSE	22	82432	1	1	1	Disabled homolog 2
DC1I2_MOUSE	47	68751	1	1	1	Cytoplasmic dynein 1 intermediate chain 2
DCAF8_MOUSE	20	66675	1	1	1	DDB1- and CUL4-associated factor 8
DCAM1_MOUSE	24	38647	1	1	1	S-adenosylmethionine decarboxylase proenzyme 1
DCNL2_MOUSE	161	30334	18	3	3	DCN1-like protein 2
DCTN1_MOUSE	23	142272	1	1	1	Dynactin subunit 1
DCTN2_MOUSE	47	44204	4	2	2	Dynactin subunit 2
DCUP_MOUSE	136	40951	5	1	1	Uroporphyrinogen decarboxylase
DCXR_MOUSE	34	25958	3	1	1	L-xylulose reductase
DD19A_MOUSE	29	54355	1	1	1	ATP-dependent RNA helicase DDX19A

DDB1_MOUSE	989	128027	85	15	15	DNA damage-binding protein 1
DDX1_MOUSE	223	83474	9	1	1	ATP-dependent RNA helicase DDX1
DDX3L_MOUSE	72	73494	3	1	1	Putative ATP-dependent RNA helicase Pl10
DDX6_MOUSE	33	54556	2	1	1	Probable ATP-dependent RNA helicase DDX6
DECR_MOUSE	2720	36476	139	9	9	2,4-dienoyl-CoA reductase, mitochondrial
DENR_MOUSE	34	22551	1	1	1	Density-regulated protein
DESM_MOUSE	461	53522	12	2	1	Desmin
DEST_MOUSE	308	18852	37	5	4	Destrin
DHB4_MOUSE	41	79945	1	1	1	Peroxisomal multifunctional enzyme type 2
DHB8_MOUSE	149	26800	13	3	3	Estradiol 17-beta-dehydrogenase 8
DHDH_MOUSE	4543	36620	199	10	10	Trans-1,2-dihydrobenzene-1,2-diol dehydrogenase
DHE3_MOUSE	812	61640	70	12	12	Glutamate dehydrogenase 1, mitochondrial
DHPR_MOUSE	1312	25782	97	7	7	Dihydropteridine reductase
DHR11_MOUSE	112	28712	10	3	3	Dehydrogenase/reductase SDR family member 11
DHRS4_MOUSE	39	30094	5	3	3	Dehydrogenase/reductase SDR family member 4
DHX8_MOUSE	33	143337	1	1	1	ATP-dependent RNA helicase DHX8
DIAP1_MOUSE	726	140053	37	5	5	Protein diaphanous homolog 1
DLDH_MOUSE	8537	54751	378	18	18	Dihydrolipoyl dehydrogenase, mitochondrial
DMD_MOUSE	131	427676	9	8	8	Dystrophin
DMKN_MOUSE	27	51858	1	1	1	Dermokine
DNJA2_MOUSE	27	46344	2	1	1	DnaJ homolog subfamily A member 2
DNJA4_MOUSE	36	45500	2	1	1	DnaJ homolog subfamily A member 4
DNM1L_MOUSE	3136	83120	157	21	21	Dynamin-1-like protein
DNPEP_MOUSE	238	52744	37	7	7	Aspartyl aminopeptidase
DOPD_MOUSE	752	13183	42	6	6	D-dopachrome decarboxylase
DPP3_MOUSE	28	83245	3	3	3	Dipeptidyl peptidase 3
DPP9_MOUSE	17	98965	1	1	1	Dipeptidyl peptidase 9
DPYL2_MOUSE	2028	62638	105	11	11	Dihydropyrimidinase-related protein 2
DRG2_MOUSE	22	41092	1	1	1	Developmentally-regulated GTP-binding protein 2
DSCAM_MOUSE	138	223897	11	1	1	Down syndrome cell adhesion molecule homolog
DUPD1_MOUSE	413	24405	30	7	7	Dual specificity phosphatase DUPD1
DUS23_MOUSE	102	16855	9	3	3	Dual specificity protein phosphatase 23
DUS28_MOUSE	78	17779	9	1	1	Dual specificity phosphatase 28
DUS3_MOUSE	2495	20687	111	6	6	Dual specificity protein phosphatase 3
DX39A_MOUSE	26	49549	1	1	1	ATP-dependent RNA helicase DDX39A
DYH1_MOUSE	32	490749	2	1	1	Dynein heavy chain 1, axonemal
DYHC1_MOUSE	236	534447	24	10	10	Cytoplasmic dynein 1 heavy chain 1
DYHC2_MOUSE	53	495451	13	1	1	Cytoplasmic dynein 2 heavy chain 1
DYL1_MOUSE	219	10530	12	2	2	Dynein light chain 1, cytoplasmic
DYN2_MOUSE	66	98426	4	3	3	Dynamin-2
DYSF_MOUSE	542	240040	20	4	4	Dysferlin
DZIP3_MOUSE	19	139586	1	1	1	E3 ubiquitin-protein ligase DZIP3
ECH1_MOUSE	423	36437	49	8	8	Delta(3,5)-Delta(2,4)-dienoyl-CoA isomerase, mitochondrial
ECHA_MOUSE	9011	83302	441	29	29	Trifunctional enzyme subunit alpha, mitochondrial
ECHB_MOUSE	4174	51639	226	16	16	Trifunctional enzyme subunit beta, mitochondrial
ECHD1_MOUSE	76	35786	4	1	1	Ethylmalonyl-CoA decarboxylase
ECHD2_MOUSE	171	32174	10	2	2	Enoyl-CoA hydratase domain-containing protein 2, mitochondrial
ECHM_MOUSE	2459	31853	148	10	10	Enoyl-CoA hydratase, mitochondrial
ECI1_MOUSE	6075	32515	299	12	12	Enoyl-CoA delta isomerase 1, mitochondrial
ECI2_MOUSE	46	43639	4	1	1	Enoyl-CoA delta isomerase 2, mitochondrial
ECM29_MOUSE	68	205398	7	5	5	Proteasome adapter and scaffold protein ECM29
EEA1_MOUSE	19	161958	1	1	1	Early endosome antigen 1
EF1A1_MOUSE	13274	50424	534	15	7	Elongation factor 1-alpha 1
EF1A2_MOUSE	19139	50764	893	21	13	Elongation factor 1-alpha 2
EF1B_MOUSE	57	24849	4	2	2	Elongation factor 1-beta
EF1D_MOUSE	1231	31388	84	5	5	Elongation factor 1-delta
EF1G_MOUSE	4024	50371	281	20	20	Elongation factor 1-gamma
EF2_MOUSE	22664	96222	1143	44	44	Elongation factor 2
EFGM_MOUSE	59	84238	3	1	1	Elongation factor G, mitochondrial
EFTU_MOUSE	3974	49876	185	19	19	Elongation factor Tu, mitochondrial
EH1L1_MOUSE	303	185634	22	4	4	EH domain-binding protein 1-like protein 1
EHD1_MOUSE	314	60622	27	3	3	EH domain-containing protein 1
EHD2_MOUSE	4267	61307	242	15	14	EH domain-containing protein 2
EHD4_MOUSE	272	61670	28	4	3	EH domain-containing protein 4
EI2BA_MOUSE	22	34023	1	1	1	Translation initiation factor eIF-2B subunit alpha
EI3JA_MOUSE	572	29440	24	4	4	Eukaryotic translation initiation factor 3 subunit J-A
EIF1B_MOUSE	21	12930	2	1	1	Eukaryotic translation initiation factor 1b
EIF3D_MOUSE	25	64576	1	1	1	Eukaryotic translation initiation factor 3 subunit D
EIF3E_MOUSE	19	52587	1	1	1	Eukaryotic translation initiation factor 3 subunit E
EIF3F_MOUSE	88	38074	7	2	2	Eukaryotic translation initiation factor 3 subunit F
EIF3I_MOUSE	26	36837	2	2	2	Eukaryotic translation initiation factor 3 subunit I
EIF3L_MOUSE	55	66798	4	2	2	Eukaryotic translation initiation factor 3 subunit L
EIF3M_MOUSE	81	42946	5	1	1	Eukaryotic translation initiation factor 3 subunit M
ELOB_MOUSE	25	13219	2	2	2	Elongin-B
ELOC_MOUSE	22	12636	1	1	1	Elongin-C
EMAL1_MOUSE	269	90650	31	8	8	Echinoderm microtubule-associated protein-like 1
EMAL2_MOUSE	361	71602	25	5	5	Echinoderm microtubule-associated protein-like 2
ENOA_MOUSE	128808	47453	4612	26	18	Alpha-enolase
ENOB_MOUSE	206113	47337	7237	45	36	Beta-enolase
ENOG_MOUSE	90233	47609	3423	11	2	Gamma-enolase
ENOPH_MOUSE	225	28696	21	1	1	Enolase-phosphatase E1
ENPL_MOUSE	1057	92703	44	10	8	Endoplasmin
ENTP2_MOUSE	29	54912	2	1	1	Ectonucleoside triphosphate diphosphohydrolase 2

EPDR1_MOUSE	70	25868	4	2	2	Mammalian ependymin-related protein 1
ERF1_MOUSE	29	49228	2	2	2	Eukaryotic peptide chain release factor subunit 1
ERF3A_MOUSE	30	69380	3	2	2	Eukaryotic peptide chain release factor GTP-binding subunit ERF3A
ERGI2_MOUSE	26	42740	2	1	1	Endoplasmic reticulum-Golgi intermediate compartment protein 2
ERP44_MOUSE	593	47222	20	2	2	Endoplasmic reticulum resident protein 44
ES1_MOUSE	3354	28415	214	10	10	ES1 protein homolog, mitochondrial
EST1C_MOUSE	3245	61302	213	13	10	Carboxylesterase 1C
EST2C_MOUSE	538	62714	28	8	8	Acylcarnitine hydrolase
ESTD_MOUSE	2220	31870	137	10	10	S-formylglutathione hydrolase
ETFA_MOUSE	11016	35330	478	16	16	Electron transfer flavoprotein subunit alpha, mitochondrial
ETFB_MOUSE	12939	27834	514	13	13	Electron transfer flavoprotein subunit beta
ETFD_MOUSE	33	68903	1	1	1	Electron transfer flavoprotein-ubiquinone oxidoreductase, mitochondrial
ETHE1_MOUSE	249	28234	20	3	3	Persulfide dioxygenase ETHE1, mitochondrial
EXT1_MOUSE	19	86994	2	1	1	Exostosin-1
F10A1_MOUSE	40	41801	2	1	1	Hsc70-interacting protein
F16P2_MOUSE	2991	37209	152	11	11	Fructose-1,6-bisphosphatase isozyme 2
F261_MOUSE	85	55385	6	3	3	6-phosphofructo-2-kinase/fructose-2,6-bisphosphatase 1
FA12_MOUSE	74	67939	3	1	1	Coagulation factor XII
FA83B_MOUSE	26	115119	5	1	1	Protein FAM83B
FABP4_MOUSE	8428	14755	371	10	10	Fatty acid-binding protein, adipocyte
FABP5_MOUSE	77	15470	6	2	2	Fatty acid-binding protein, epidermal
FABPH_MOUSE	8400	14810	396	10	10	Fatty acid-binding protein, heart
FAD1_MOUSE	18	55245	1	1	1	FAD synthase
FAHD1_MOUSE	463	25498	37	2	2	Acylpyruvase FAHD1, mitochondrial
FAHD2_MOUSE	125	35067	14	4	4	Fumarylacetoacetate hydrolase domain-containing protein 2A
FAK2_MOUSE	19	117032	1	1	1	Protein-tyrosine kinase 2-beta
FAS_MOUSE	3575	274994	224	47	47	Fatty acid synthase
FCER2_MOUSE	49	38251	5	1	1	Low affinity immunoglobulin epsilon Fc receptor
FCL_MOUSE	134	36083	17	3	3	GDP-L-fucose synthase
FERM2_MOUSE	32	78435	2	2	2	Fermitin family homolog 2
FETA_MOUSE	19	69118	2	1	1	Alpha-fetoprotein
FETUA_MOUSE	4807	38100	212	9	9	Alpha-2-HS-glycoprotein
FETUB_MOUSE	87	43541	5	2	2	Fetuin-B
FHL1_MOUSE	11071	33806	576	17	17	Four and a half LIM domains protein 1
FHL3_MOUSE	24	33769	1	1	1	Four and a half LIM domains protein 3
FHOD3_MOUSE	52	176630	6	1	1	FH1/FH2 domain-containing protein 3
FIBA_MOUSE	1590	88117	105	14	14	Fibrinogen alpha chain
FIBB_MOUSE	2094	55402	166	18	18	Fibrinogen beta chain
FIBG_MOUSE	2990	50044	164	13	13	Fibrinogen gamma chain
FIGL1_MOUSE	20	75716	1	1	1	Fidgetin-like protein 1
FKB1A_MOUSE	1075	11972	48	3	3	Peptidyl-prolyl cis-trans isomerase FKBP1A
FKBP3_MOUSE	145	25189	12	3	3	Peptidyl-prolyl cis-trans isomerase FKBP3
FLNA_MOUSE	1376	283897	60	7	3	Filamin-A
FLNC_MOUSE	5702	293560	324	43	39	Filamin-C
FMOD_MOUSE	169	43427	14	3	3	Fibromodulin
FN3K_MOUSE	183	35238	11	1	1	Fructosamine-3-kinase
FNTA_MOUSE	239	44157	16	2	2	Protein farnesyltransferase/geranylgeranyltransferase type-1 subunit alpha
FNTB_MOUSE	24	49757	1	1	1	Protein farnesyltransferase subunit beta
FRIH_MOUSE	2478	21224	164	11	11	Ferritin heavy chain
FRIL1_MOUSE	3244	20847	126	9	9	Ferritin light chain 1
FSCN1_MOUSE	45	55215	5	2	2	Fascin
FUMH_MOUSE	11789	54550	609	20	20	Fumarate hydratase, mitochondrial
FXR1_MOUSE	120	76517	5	2	2	Fragile X mental retardation syndrome-related protein 1
FYB2_MOUSE	75	82815	4	2	2	FYN-binding protein 2
G3P_MOUSE	127934	36072	5292	35	35	Glyceraldehyde-3-phosphate dehydrogenase
G3ST2_MOUSE	22	47107	1	1	1	Galactose-3-O-sulfotransferase 2
G6PE_MOUSE	26	89442	1	1	1	GDH/6PGL endoplasmic bifunctional protein
G6PI_MOUSE	39239	62955	1907	34	34	Glucose-6-phosphate isomerase
GALK1_MOUSE	17	42668	1	1	1	Galactokinase
GARS_MOUSE	3249	82624	131	18	18	Glycine--tRNA ligase
GBB1_MOUSE	59	38151	3	2	2	Guanine nucleotide-binding protein G(I)/G(S)/G(T) subunit beta-1
GCAA_MOUSE	1190	36936	87	8	8	Ig gamma-2A chain C region, A allele
GCDH_MOUSE	40	49088	3	1	1	Glutaryl-CoA dehydrogenase, mitochondrial
GDIA_MOUSE	3256	51059	125	9	5	Rab GDP dissociation inhibitor alpha
GDIB_MOUSE	8222	51018	339	23	19	Rab GDP dissociation inhibitor beta
GDIR1_MOUSE	1965	23450	104	7	7	Rho GDP-dissociation inhibitor 1
GDIR2_MOUSE	21	22894	1	1	1	Rho GDP-dissociation inhibitor 2
GELS_MOUSE	331	86287	34	6	6	Gelsolin
GEPH_MOUSE	32	83971	2	2	2	Gephyrin
GGA1_MOUSE	19	70270	1	1	1	ADP-ribosylation factor-binding protein GGA1
GGYF2_MOUSE	18	149387	1	1	1	GRB10-interacting GYF protein 2
GLGB_MOUSE	2838	80769	145	17	17	1,4-alpha-glucan-branching enzyme
GLNA_MOUSE	283	42834	16	3	3	Glutamine synthetase
GLO2_MOUSE	288	34519	26	3	3	Hydroxyacylglutathione hydrolase, mitochondrial
GLOD4_MOUSE	1664	33581	115	12	12	Glyoxalase domain-containing protein 4
GLRX1_MOUSE	2530	12148	107	4	4	Glutaredoxin-1
GLRX3_MOUSE	709	38039	45	8	8	Glutaredoxin-3
GLUCM_MOUSE	842	67236	39	10	10	D-glutamate cyclase, mitochondrial
GLYC_MOUSE	42	53138	3	1	1	Serine hydroxymethyltransferase, cytosolic
GLYG_MOUSE	1519	37606	95	8	8	Glycogenin-1
GMFB_MOUSE	396	16884	17	2	2	Glia maturation factor beta
GMPR1_MOUSE	2207	37971	107	10	10	GMP reductase 1
GNAL_MOUSE	47	44794	5	1	1	Guanine nucleotide-binding protein G(olf) subunit alpha

GP101_MOUSE	19	56893	1	1	1	Probable G-protein coupled receptor 101
GPD1L_MOUSE	3746	38828	146	14	13	Glycerol-3-phosphate dehydrogenase 1-like protein
GPDA_MOUSE	26901	38176	1206	24	23	Glycerol-3-phosphate dehydrogenase [NAD(+)], cytoplasmic
GPR87_MOUSE	20	42014	1	1	1	G-protein coupled receptor 87
GPX1_MOUSE	188	22684	25	3	3	Glutathione peroxidase 1
GPX3_MOUSE	272	25720	33	3	3	Glutathione peroxidase 3
GPX4_MOUSE	540	22925	26	3	3	Phospholipid hydroperoxide glutathione peroxidase
GRB2_MOUSE	23	25336	2	1	1	Growth factor receptor-bound protein 2
GRHPR_MOUSE	2964	35706	180	11	11	Glyoxylate reductase/hydroxypyruvate reductase
GRP75_MOUSE	2383	73701	158	20	20	Stress-70 protein, mitochondrial
GRPE1_MOUSE	186	24520	16	2	2	GrpE protein homolog 1, mitochondrial
GSHB_MOUSE	34	52442	2	1	1	Glutathione synthetase
GSHR_MOUSE	1207	54256	74	8	8	Glutathione reductase, mitochondrial
GSTA3_MOUSE	135	25401	19	2	1	Glutathione S-transferase A3
GSTA4_MOUSE	1056	25547	91	8	7	Glutathione S-transferase A4
GSTK1_MOUSE	173	25801	9	3	3	Glutathione S-transferase kappa 1
GSTM1_MOUSE	9929	26067	477	19	16	Glutathione S-transferase Mu 1
GSTM2_MOUSE	6539	25871	336	18	15	Glutathione S-transferase Mu 2
GSTM5_MOUSE	4417	27016	195	13	12	Glutathione S-transferase Mu 5
GSTO1_MOUSE	148	27708	14	6	6	Glutathione S-transferase omega-1
GSTP1_MOUSE	11558	23765	524	10	3	Glutathione S-transferase P 1
GSTP2_MOUSE	6122	23693	290	9	2	Glutathione S-transferase P 2
GSTT1_MOUSE	31	27641	4	2	2	Glutathione S-transferase theta-1
GYS1_MOUSE	3627	84672	225	20	20	Glycogen [starch] synthase, muscle
H10_MOUSE	45	20848	2	2	2	Histone H1.0
H12_MOUSE	297	21254	19	6	6	Histone H1.2
H2A2A_MOUSE	166	14087	15	4	4	Histone H2A type 2-A
H2B1A_MOUSE	107	14285	11	2	2	Histone H2B type 1-A
H3C_MOUSE	75	15363	9	2	2	Histone H3.3C
H4_MOUSE	82	11360	9	2	2	Histone H4
HA10_MOUSE	105	37455	5	3	3	H-2 class I histocompatibility antigen, Q10 alpha chain
HBA_MOUSE	19513	15133	941	11	11	Hemoglobin subunit alpha
HBB1_MOUSE	44005	15944	1570	20	11	Hemoglobin subunit beta-1
HBB2_MOUSE	26001	15982	1078	16	7	Hemoglobin subunit beta-2
HCD2_MOUSE	3139	27516	147	9	9	3-hydroxyacyl-CoA dehydrogenase type-2
HCDH_MOUSE	6541	34613	341	17	17	Hydroxyacyl-coenzyme A dehydrogenase, mitochondrial
HDDC2_MOUSE	89	22909	2	1	1	HD domain-containing protein 2
HDGF_MOUSE	151	26367	9	2	2	Hepatoma-derived growth factor
HDHD2_MOUSE	1716	28883	121	7	7	Haloacid dehalogenase-like hydrolase domain-containing protein 2
HDHD5_MOUSE	329	46562	23	5	5	Haloacid dehalogenase-like hydrolase domain-containing 5
HECD1_MOUSE	72	292870	2	1	1	E3 ubiquitin-protein ligase HECTD1
HEM2_MOUSE	2407	36456	133	11	11	Delta-aminolevulinic acid dehydratase
HEM3_MOUSE	197	39605	16	3	3	Porphobilinogen deaminase
HEM4_MOUSE	17	28885	1	1	1	Uroporphyrinogen-III synthase
HEMH_MOUSE	37	47670	1	1	1	Ferrochelatase, mitochondrial
HEMO_MOUSE	17810	52026	908	26	26	Hemopexin
HEP2_MOUSE	375	54747	29	9	9	Heparin cofactor 2
HHATL_MOUSE	390	57021	27	6	6	Protein-cysteine N-palmitoyltransferase HHAT-like protein
HIBCH_MOUSE	1140	43295	96	13	13	3-hydroxyisobutyryl-CoA hydrolase, mitochondrial
HINT1_MOUSE	3866	13882	206	6	6	Histidine triad nucleotide-binding protein 1
HINT2_MOUSE	205	17366	14	3	3	Histidine triad nucleotide-binding protein 2, mitochondrial
HMGB1_MOUSE	550	25049	45	3	3	High mobility group protein B1
HMGCL_MOUSE	294	34673	18	4	4	Hydroxymethylglutaryl-CoA lyase, mitochondrial
HNRPD_MOUSE	26	38501	2	1	1	Heterogeneous nuclear ribonucleoprotein D0
HNRPK_MOUSE	2185	51230	107	9	9	Heterogeneous nuclear ribonucleoprotein K
HNRPU_MOUSE	49	88661	2	2	2	Heterogeneous nuclear ribonucleoprotein U
HOOK3_MOUSE	43	83566	2	1	1	Protein Hook homolog 3
HOT_MOUSE	20	50362	1	1	1	Hydroxyacid-oxoacid transhydrogenase, mitochondrial
HPRT_MOUSE	182	24783	10	3	3	Hypoxanthine-guanine phosphoribosyltransferase
HPT_MOUSE	1255	39241	66	8	8	Haptoglobin
HRG_MOUSE	203	60095	13	6	6	Histidine-rich glycoprotein
HS3S2_MOUSE	27	41865	8	1	1	Heparan sulfate glucosamine 3-O-sulfotransferase 2
HS71A_MOUSE	2978	70321	160	7	3	Heat shock 70 kDa protein 1A
HS90A_MOUSE	5944	85134	294	18	7	Heat shock protein HSP 90-alpha
HS90B_MOUSE	16385	83571	887	35	22	Heat shock protein HSP 90-beta
HSDL2_MOUSE	38	54516	2	2	2	Hydroxysteroid dehydrogenase-like protein 2
HSP74_MOUSE	1727	94872	100	17	17	Heat shock 70 kDa protein 4
HSP7C_MOUSE	20369	71055	1011	37	32	Heat shock cognate 71 kDa protein
HSPB1_MOUSE	4798	23057	283	13	13	Heat shock protein beta-1
HSPB2_MOUSE	821	20419	61	3	3	Heat shock protein beta-2
HSPB6_MOUSE	3377	17567	170	9	9	Heat shock protein beta-6
HSPB7_MOUSE	357	18680	15	2	2	Heat shock protein beta-7
HTR5B_MOUSE	29	226799	1	1	1	HEAT repeat-containing protein 5B
HUTH_MOUSE	22	72897	1	1	1	Histidine ammonia-lyase
HUWE1_MOUSE	62	486267	3	2	2	E3 ubiquitin-protein ligase HUWE1
HVM12_MOUSE	72	13089	3	1	1	Ig heavy chain V region MOPC 104E
HVM27_MOUSE	96	12781	4	1	1	Ig heavy chain V-III region A4
HVM56_MOUSE	30	10768	1	1	1	Ig heavy chain V region 914
HXK2_MOUSE	3713	103951	249	31	31	Hexokinase-2
HYAL2_MOUSE	18	54211	1	1	1	Hyaluronidase-2
HYES_MOUSE	779	63045	49	6	6	Bifunctional epoxide hydrolase 2
IC1_MOUSE	160	55834	6	4	4	Plasma protease C1 inhibitor
ICA_MOUSE	687	78711	31	4	3	Inhibitor of carbonic anhydrase

IDE_MOUSE	103	118438	12	4	4	Insulin-degrading enzyme
IDH3A_MOUSE	9931	40069	486	16	16	Isocitrate dehydrogenase [NAD] subunit alpha, mitochondrial
IDHC_MOUSE	1827	47044	128	16	15	Isocitrate dehydrogenase [NADP] cytoplasmic
IDHG1_MOUSE	4915	43157	307	13	13	Isocitrate dehydrogenase [NAD] subunit gamma 1, mitochondrial
IDHP_MOUSE	13760	51330	747	26	25	Isocitrate dehydrogenase [NADP], mitochondrial
IF2A_MOUSE	104	36371	4	2	2	Eukaryotic translation initiation factor 2 subunit 1
IF2G_MOUSE	76	51603	6	1	1	Eukaryotic translation initiation factor 2 subunit 3, X-linked
IF2P_MOUSE	34	137988	1	1	1	Eukaryotic translation initiation factor 5B
IF4A1_MOUSE	3637	46353	194	13	5	Eukaryotic initiation factor 4A-I
IF4A2_MOUSE	3402	46601	185	13	5	Eukaryotic initiation factor 4A-II
IF4B_MOUSE	169	68970	9	2	2	Eukaryotic translation initiation factor 4B
IF4E_MOUSE	77	25266	9	1	1	Eukaryotic translation initiation factor 4E
IF4G1_MOUSE	255	176823	18	6	6	Eukaryotic translation initiation factor 4 gamma 1
IF4G3_MOUSE	121	176092	4	1	1	Eukaryotic translation initiation factor 4 gamma 3
IF4H_MOUSE	97	27381	5	1	1	Eukaryotic translation initiation factor 4H
IF5A1_MOUSE	3662	17049	171	7	7	Eukaryotic translation initiation factor 5A-1
IF6_MOUSE	117	27007	6	2	2	Eukaryotic translation initiation factor 6
IGG2B_MOUSE	763	44972	48	6	6	Ig gamma-2B chain C region
IGH1M_MOUSE	3084	44043	136	11	11	Ig gamma-1 chain C region, membrane-bound form
IGHG3_MOUSE	87	44472	8	5	5	Ig gamma-3 chain C region
IGHM_MOUSE	389	50625	29	6	6	Immunoglobulin heavy constant mu
IGKC_MOUSE	1217	12098	86	7	7	Immunoglobulin kappa constant
IGS10_MOUSE	31	287757	3	1	1	Immunoglobulin superfamily member 10
IL1R2_MOUSE	30	46130	1	1	1	Interleukin-1 receptor type 2
ILEUA_MOUSE	537	42719	39	8	7	Leukocyte elastase inhibitor A
ILK_MOUSE	28	51853	1	1	1	Integrin-linked protein kinase
IMA3_MOUSE	414	58400	30	6	3	Importin subunit alpha-3
IMA4_MOUSE	485	58193	40	10	7	Importin subunit alpha-4
IMA5_MOUSE	77	60886	5	1	1	Importin subunit alpha-5
IMB1_MOUSE	530	98377	40	11	11	Importin subunit beta-1
IMDH1_MOUSE	27	55586	1	1	1	Inosine-5'-monophosphate dehydrogenase 1
IMDH2_MOUSE	25	56179	2	1	1	Inosine-5'-monophosphate dehydrogenase 2
IMPA1_MOUSE	1127	30815	56	4	4	Inositol monophosphatase 1
INHBA_MOUSE	70	48160	16	1	1	Inhibin beta A chain
INMT_MOUSE	1035	30068	57	5	5	Indolethylamine N-methyltransferase
INT4_MOUSE	31	109093	1	1	1	Integrator complex subunit 4
IPKA_MOUSE	79	7956	5	2	2	cAMP-dependent protein kinase inhibitor alpha
IPO5_MOUSE	761	124993	45	10	10	Importin-5
IPO7_MOUSE	213	120721	21	5	5	Importin-7
IPYR_MOUSE	1426	33102	93	9	9	Inorganic pyrophosphatase
IPYR2_MOUSE	143	38546	12	3	3	Inorganic pyrophosphatase 2, mitochondrial
IQGA1_MOUSE	55	189423	3	2	2	Ras GTPase-activating-like protein IQGAP1
IQGA2_MOUSE	51	181100	9	2	2	Ras GTPase-activating-like protein IQGAP2
IRS1_MOUSE	19	131783	1	1	1	Insulin receptor substrate 1
ISC2A_MOUSE	1677	22745	66	5	5	Isochorismatase domain-containing protein 2A
ISOC1_MOUSE	651	32297	51	9	9	Isochorismatase domain-containing protein 1
ITBP2_MOUSE	113	39541	9	1	1	Integrin beta-1-binding protein 2
ITIH1_MOUSE	76	101460	4	3	3	Inter-alpha-trypsin inhibitor heavy chain H1
ITIH2_MOUSE	664	106261	43	15	15	Inter-alpha-trypsin inhibitor heavy chain H2
ITIH3_MOUSE	279	99638	22	6	6	Inter-alpha-trypsin inhibitor heavy chain H3
ITIH4_MOUSE	606	104765	25	10	10	Inter alpha-trypsin inhibitor, heavy chain 4
ITPA_MOUSE	356	22225	42	4	4	Inosine triphosphate pyrophosphatase
ITPR3_MOUSE	40	306931	3	1	1	Inositol 1,4,5-trisphosphate receptor type 3
IVD_MOUSE	2229	46695	161	12	12	Isovaleryl-CoA dehydrogenase, mitochondrial
JIP4_MOUSE	79	146984	2	1	1	C-Jun-amino-terminal kinase-interacting protein 4
JPH1_MOUSE	38	72374	3	2	2	Junctophilin-1
JPH2_MOUSE	28	74818	1	1	1	Junctophilin-2
JSPR1_MOUSE	32	36242	2	1	1	Junctional sarcoplasmic reticulum protein 1
K0319_MOUSE	28	119055	1	1	1	Dyslexia-associated protein KIAA0319 homolog
K1109_MOUSE	24	559349	1	1	1	Uncharacterized protein KIAA1109
K1C14_MOUSE	73	53176	5	4	3	Keratin, type I cytoskeletal 14
K22O_MOUSE	157	63319	11	2	1	Keratin, type II cytoskeletal 2 oral
K2C6A_MOUSE	461	59641	22	3	1	Keratin, type II cytoskeletal 6A
KAD1_MOUSE	43152	21640	1389	18	18	Adenylate kinase isoenzyme 1
KAD2_MOUSE	47	26737	3	1	1	Adenylate kinase 2, mitochondrial
KAD4_MOUSE	24	25160	2	2	2	Adenylate kinase 4, mitochondrial
KANK4_MOUSE	44	111077	2	1	1	KN motif and ankyrin repeat domain-containing protein 4
KAP0_MOUSE	89	43443	10	4	4	cAMP-dependent protein kinase type I-alpha regulatory subunit
KAP2_MOUSE	1376	45703	99	11	11	cAMP-dependent protein kinase type II-alpha regulatory subunit
KAPCA_MOUSE	1751	40659	107	10	4	cAMP-dependent protein kinase catalytic subunit alpha
KAPCB_MOUSE	776	40853	73	8	2	cAMP-dependent protein kinase catalytic subunit beta
KAT1_MOUSE	334	47932	31	4	4	Kynurenine--oxoglutarate transaminase 1
KAT6B_MOUSE	33	211074	1	1	1	Histone acetyltransferase KAT6B
KCC2A_MOUSE	253	54651	26	3	2	Calcium/calmodulin-dependent protein kinase type II subunit alpha
KCC2B_MOUSE	678	61164	41	6	3	Calcium/calmodulin-dependent protein kinase type II subunit beta
KCC2D_MOUSE	619	56961	38	4	1	Calcium/calmodulin-dependent protein kinase type II subunit delta
KCNF1_MOUSE	32	56410	1	1	1	Potassium voltage-gated channel subfamily F member 1
KCRB_MOUSE	1718	42971	139	11	10	Creatine kinase B-type
KCRM_MOUSE	449248	43246	17966	51	49	Creatine kinase M-type
KCRS_MOUSE	31274	47899	1395	26	25	Creatine kinase S-type, mitochondrial
KCY_MOUSE	839	22379	42	6	6	UMP-CMP kinase
KERA_MOUSE	30	40777	1	1	1	Keratocan
KIF11_MOUSE	27	118980	8	1	1	Kinesin-like protein KIF11

KIF4_MOUSE	26	141029	1	1	1	Chromosome-associated kinesin KIF4
KINH_MOUSE	248	110225	16	5	5	Kinesin-1 heavy chain
KLC1_MOUSE	39	61868	3	2	2	Kinesin light chain 1
KLH31_MOUSE	632	71163	22	3	3	Kelch-like protein 31
KLH40_MOUSE	52	70400	2	2	2	Kelch-like protein 40
KLH41_MOUSE	127	68945	15	6	6	Kelch-like protein 41
KNG1_MOUSE	2934	74140	209	15	15	Kininogen-1
KPB1_MOUSE	4450	139877	253	27	27	Phosphorylase b kinase regulatory subunit alpha, skeletal muscle isoform
KPBB_MOUSE	3193	124951	176	21	21	Phosphorylase b kinase regulatory subunit beta
KPCT_MOUSE	47	83059	2	1	1	Protein kinase C theta type
KPRA_MOUSE	733	39692	45	6	5	Phosphoribosyl pyrophosphate synthase-associated protein 1
KPRB_MOUSE	425	41254	34	7	6	Phosphoribosyl pyrophosphate synthase-associated protein 2
KPYM_MOUSE	102386	58378	3969	41	41	Pyruvate kinase PKM
KS6A3_MOUSE	355	83983	25	5	5	Ribosomal protein S6 kinase alpha-3
KT3K_MOUSE	27	34674	1	1	1	Ketosamine-3-kinase
KV2A7_MOUSE	111	12379	3	1	1	Ig kappa chain V-II region 26-10
KV3A1_MOUSE	41	12087	1	1	1	Ig kappa chain V-III region PC 2880/PC 1229
LACB2_MOUSE	109	33019	12	1	1	Endoribonuclease LACTB2
LANC1_MOUSE	18	46053	1	1	1	LanC-like protein 1
LANC2_MOUSE	24	51486	1	1	1	LanC-like protein 2
LCAP_MOUSE	22	117799	1	1	1	Leucyl-cystinyl aminopeptidase
LDB3_MOUSE	6984	77581	396	18	18	LIM domain-binding protein 3
LDHA_MOUSE	50403	36817	2116	33	31	L-lactate dehydrogenase A chain
LDHB_MOUSE	13148	36834	560	21	19	L-lactate dehydrogenase B chain
LEG1_MOUSE	2490	15198	167	7	7	Galectin-1
LEGL_MOUSE	79	19172	5	2	2	Galectin-related protein
LGUL_MOUSE	5763	20967	340	12	12	Lactoylglutathione lyase
LIS1_MOUSE	29	47210	2	1	1	Platelet-activating factor acetylhydrolase IB subunit alpha
LKHA4_MOUSE	1409	69634	76	14	14	Leukotriene A-4 hydrolase
LMCD1_MOUSE	1334	42167	93	16	16	LIM and cysteine-rich domains protein 1
LONM_MOUSE	407	106347	34	8	8	Lon protease homolog, mitochondrial
LOX5_MOUSE	24	78602	1	1	1	Arachidonate 5-lipoxygenase
LPPRC_MOUSE	816	157770	70	17	17	Leucine-rich PPR motif-containing protein, mitochondrial
LRC20_MOUSE	246	20910	34	5	5	Leucine-rich repeat-containing protein 20
LRC47_MOUSE	21	64178	1	1	1	Leucine-rich repeat-containing protein 47
LRC49_MOUSE	39	79397	4	1	1	Leucine-rich repeat-containing protein 49
LRP1_MOUSE	28	523342	1	1	1	Prolow-density lipoprotein receptor-related protein 1
LUM_MOUSE	2553	38640	178	8	8	Lumican
LX12E_MOUSE	20	76092	1	1	1	Arachidonate 12-lipoxygenase, epidermal-type
LYAG_MOUSE	59	106921	2	2	2	Lysosomal alpha-glucosidase
LYPA1_MOUSE	2542	25014	83	6	6	Acyl-protein thioesterase 1
LYPA2_MOUSE	502	25120	24	4	4	Acyl-protein thioesterase 2
LYRM7_MOUSE	18	12096	1	1	1	Complex III assembly factor LYRM7
LZTL1_MOUSE	18	34809	1	1	1	Leucine zipper transcription factor-like protein 1
M2OM_MOUSE	224	34304	16	4	4	Mitochondrial 2-oxoglutarate/malate carrier protein
M3K20_MOUSE	301	92232	21	5	5	Mitogen-activated protein kinase kinase kinase 20
MA2C1_MOUSE	31	116983	3	2	2	Alpha-mannosidase 2C1
MAAI_MOUSE	615	24431	25	5	5	Maleylacetoacetate isomerase
MABP1_MOUSE	18	164615	1	1	1	Mitogen-activated protein kinase-binding protein 1
MACD1_MOUSE	10798	35843	336	10	10	O-acetyl-ADP-ribose deacetylase MACROD1
MAON_MOUSE	2184	67683	92	10	10	NADP-dependent malic enzyme, mitochondrial
MAOX_MOUSE	5588	64540	326	22	22	NADP-dependent malic enzyme
MAP11_MOUSE	38	44163	3	1	1	Methionine aminopeptidase 1
MAP4_MOUSE	237	117927	25	9	9	Microtubule-associated protein 4
MAPK2_MOUSE	19	44421	1	1	1	MAP kinase-activated protein kinase 2
MARE3_MOUSE	44	32231	2	1	1	Microtubule-associated protein RP/EB family member 3
MAT2B_MOUSE	116	37768	9	3	3	Methionine adenosyltransferase 2 subunit beta
MBP_MOUSE	20	27151	1	1	1	Myelin basic protein
MCA3_MOUSE	45	19903	6	1	1	Eukaryotic translation elongation factor 1 epsilon-1
MCCB_MOUSE	55	61910	3	1	1	Methylcrotonoyl-CoA carboxylase beta chain, mitochondrial
MCTS1_MOUSE	30	20770	1	1	1	Malignant T-cell-amplified sequence 1
MDHC_MOUSE	25441	36659	1014	18	18	Malate dehydrogenase, cytoplasmic
MDHM_MOUSE	47535	36045	1691	22	22	Malate dehydrogenase, mitochondrial
MECR_MOUSE	28	40545	4	3	3	Enoyl-[acyl-carrier-protein] reductase, mitochondrial
MED23_MOUSE	17	157925	1	1	1	Mediator of RNA polymerase II transcription subunit 23
MEG11_MOUSE	18	124417	1	1	1	Multiple epidermal growth factor-like domains protein 11
MEII1_MOUSE	23	142723	1	1	1	Meiosis inhibitor protein 1
MEMO1_MOUSE	72	34070	6	1	1	Protein MEMO1
MESH1_MOUSE	23	20307	1	1	1	Guanosine-3',5'-bis(diphosphate) 3'-pyrophosphohydrolase MESH1
MET13_MOUSE	17	79391	1	1	1	Methyltransferase-like protein 13
METK2_MOUSE	376	44003	40	4	4	S-adenosylmethionine synthase isoform type-2
MFAP4_MOUSE	397	29261	17	1	1	Microfibril-associated glycoprotein 4
MGDP1_MOUSE	685	18628	28	2	2	Magnesium-dependent phosphatase 1
MIC60_MOUSE	109	84247	8	2	2	MIC complex subunit Mic60
MIF_MOUSE	1392	12667	80	3	3	Macrophage migration inhibitory factor
MIME_MOUSE	1358	34333	48	3	3	Mimecan
MINP1_MOUSE	64	55130	4	3	3	Multiple inositol polyphosphate phosphatase 1
MINT_MOUSE	25	399881	1	1	1	Msx2-interacting protein
MK01_MOUSE	350	41648	20	6	6	Mitogen-activated protein kinase 1
MK12_MOUSE	2676	42188	173	12	11	Mitogen-activated protein kinase 12
MK14_MOUSE	1333	41489	73	6	5	Mitogen-activated protein kinase 14
MLRS_MOUSE	7125	19057	373	13	13	Myosin regulatory light chain 2, skeletal muscle isoform
MMSA_MOUSE	4155	58335	285	20	20	Methylmalonate-semialdehyde dehydrogenase [acylating], mitochondrial

MOB2_MOUSE	26	27176	1	1	1	MOB kinase activator 2
MOCS1_MOUSE	19	70670	1	1	1	Molybdenum cofactor biosynthesis protein 1
MOES_MOUSE	868	67839	75	11	5	Moesin
MOONR_MOUSE	34	109720	3	1	1	Protein moonraker
MP2K1_MOUSE	569	43788	61	7	5	Dual specificity mitogen-activated protein kinase kinase 1
MP2K2_MOUSE	334	44659	38	4	2	Dual specificity mitogen-activated protein kinase kinase 2
MP2K3_MOUSE	866	39613	40	5	4	Dual specificity mitogen-activated protein kinase kinase 3
MP2K4_MOUSE	236	44542	13	2	2	Dual specificity mitogen-activated protein kinase kinase 4
MP2K6_MOUSE	294	37750	21	3	2	Dual specificity mitogen-activated protein kinase kinase 6
MPC1_MOUSE	365	12617	20	2	2	Mitochondrial pyruvate carrier 1
MPCP_MOUSE	792	40063	49	7	7	Phosphate carrier protein, mitochondrial
MPI_MOUSE	2481	47229	136	11	11	Mannose-6-phosphate isomerase
MPP4_MOUSE	17	73038	1	1	1	MAGUK p55 subfamily member 4
MPPA_MOUSE	1211	58755	25	5	5	Mitochondrial-processing peptidase subunit alpha
MRO2B_MOUSE	27	182188	2	2	2	Maestro heat-like repeat-containing protein family member 2B
MSH6_MOUSE	31	152813	2	1	1	DNA mismatch repair protein Msh6
MSRA_MOUSE	56	26200	9	4	4	Mitochondrial peptide methionine sulfoxide reductase
MSRB3_MOUSE	33	27442	3	2	2	Methionine-R-sulfoxide reductase B3, mitochondrial
MSTN1_MOUSE	36	8936	4	1	1	Musculoskeletal embryonic nuclear protein 1
MTAP_MOUSE	150	31612	11	2	2	S-methyl-5'-thioadenosine phosphorylase
MTBP_MOUSE	20	101619	1	1	1	Mdm2-binding protein
MTL26_MOUSE	183	23015	6	2	2	Methyltransferase-like 26
MTM1_MOUSE	24	70028	2	2	2	Myotubularin
MTNA_MOUSE	126	39671	14	3	3	Methylthioribose-1-phosphate isomerase
MTNB_MOUSE	41	27558	1	1	1	Methylthioribulose-1-phosphate dehydratase
MTND_MOUSE	21	21567	1	1	1	1,2-dihydroxy-3-keto-5-methylthiopentene dioxygenase
MTPN_MOUSE	29	13024	1	1	1	Myotrophin
MUG1_MOUSE	5623	166618	351	37	37	Murinoglobulin-1
MUP1_MOUSE	23	20920	1	1	1	Major urinary protein 1
MUTA_MOUSE	242	83248	17	3	3	Methylmalonyl-CoA mutase, mitochondrial
MY18A_MOUSE	37	233637	1	1	1	Unconventional myosin-XVIIIa
MYBPH_MOUSE	31	53065	3	2	2	Myosin-binding protein H
MYBPP_MOUSE	62	106785	8	1	1	MYCBP-associated protein
MYG_MOUSE	58248	17116	2811	18	17	Myoglobin
MYH1_MOUSE	67299	224116	3036	119	19	Myosin-1
MYH11_MOUSE	1036	227743	62	7	4	Myosin-11
MYH3_MOUSE	23819	224736	1235	50	3	Myosin-3
MYH4_MOUSE	109464	223632	4906	145	41	Myosin-4
MYH6_MOUSE	20128	224225	885	41	3	Myosin-6
MYH7_MOUSE	20784	223539	944	45	4	Myosin-7
MYH8_MOUSE	57923	223653	2575	92	5	Myosin-8
MYH9_MOUSE	2191	227429	131	15	13	Myosin-9
MYL1_MOUSE	14914	20695	613	16	13	Myosin light chain 1/3, skeletal muscle isoform
MYL3_MOUSE	2627	22521	162	10	7	Myosin light chain 3
MYL6_MOUSE	1842	17090	115	4	4	Myosin light polypeptide 6
MYLK2_MOUSE	3888	66633	310	22	22	Myosin light chain kinase 2, skeletal/cardiac muscle
MYO1A_MOUSE	18	119418	1	1	1	Unconventional myosin-Ia
MYO5B_MOUSE	27	212092	1	1	1	Unconventional myosin-Vb
MYOC_MOUSE	66	55622	6	3	3	Myocilin
MYOM1_MOUSE	11753	186546	748	64	64	Myomesin-1
MYOM3_MOUSE	71	162761	4	2	2	Myomesin-3
MYOTI_MOUSE	99	55738	10	3	3	Myotilin
MYP0_MOUSE	41	27775	3	2	2	Myelin protein P0
MYPC2_MOUSE	18574	128128	913	54	54	Myosin-binding protein C, fast-type
NACAM_MOUSE	3376	221277	247	48	48	Nascent polypeptide-associated complex subunit alpha, muscle-specific form
NAMPT_MOUSE	197	55698	25	7	7	Nicotinamide phosphoribosyltransferase
NAR3_MOUSE	36	42330	2	2	2	Ecto-ADP-ribosyltransferase 3
NCAM1_MOUSE	19	120152	1	1	1	Neural cell adhesion molecule 1
NDKA_MOUSE	6306	17311	299	8	3	Nucleoside diphosphate kinase A
NDKB_MOUSE	9628	17466	506	11	6	Nucleoside diphosphate kinase B
NDRG2_MOUSE	4447	41105	282	9	9	Protein NDRG2
NDUA4_MOUSE	310	9321	14	4	4	Cytochrome c oxidase subunit NDUFA4
NDUA5_MOUSE	67	13408	4	1	1	NADH dehydrogenase [ubiquinone] 1 alpha subcomplex subunit 5
NDUA9_MOUSE	113	42612	16	4	4	NADH dehydrogenase [ubiquinone] 1 alpha subcomplex subunit 9, mitochondrial
NDUAA_MOUSE	846	40863	29	2	2	NADH dehydrogenase [ubiquinone] 1 alpha subcomplex subunit 10, mitochondrial
NDUAC_MOUSE	155	17133	8	1	1	NADH dehydrogenase [ubiquinone] 1 alpha subcomplex subunit 12
NDUAD_MOUSE	167	16849	14	1	1	NADH dehydrogenase [ubiquinone] 1 alpha subcomplex subunit 13
NDUB4_MOUSE	34	15072	1	1	1	NADH dehydrogenase [ubiquinone] 1 beta subcomplex subunit 4
NDUB8_MOUSE	23	21919	1	1	1	NADH dehydrogenase [ubiquinone] 1 beta subcomplex subunit 8, mitochondrial
NDUC2_MOUSE	66	14211	6	2	2	NADH dehydrogenase [ubiquinone] 1 subunit C2
NDUS1_MOUSE	689	80752	66	13	13	NADH-ubiquinone oxidoreductase 75 kDa subunit, mitochondrial
NDUS3_MOUSE	549	30302	25	5	5	NADH dehydrogenase [ubiquinone] iron-sulfur protein 3, mitochondrial
NDUS5_MOUSE	116	12867	5	1	1	NADH dehydrogenase [ubiquinone] iron-sulfur protein 5
NDUS7_MOUSE	86	24952	12	2	2	NADH dehydrogenase [ubiquinone] iron-sulfur protein 7, mitochondrial
NDUS8_MOUSE	79	24479	7	1	1	NADH dehydrogenase [ubiquinone] iron-sulfur protein 8, mitochondrial
NDUV1_MOUSE	232	51486	28	6	6	NADH dehydrogenase [ubiquinone] flavoprotein 1, mitochondrial
NDUV2_MOUSE	171	27610	13	2	2	NADH dehydrogenase [ubiquinone] flavoprotein 2, mitochondrial
NEDD4_MOUSE	278	103155	23	8	8	E3 ubiquitin-protein ligase NEDD4
NEDD8_MOUSE	1351	8967	57	2	2	NEDD8
NEIL2_MOUSE	27	37210	3	1	1	Endonuclease 8-like 2
NEK9_MOUSE	33	108843	1	1	1	Serine/threonine-protein kinase Nek9
NHLC2_MOUSE	23	79236	1	1	1	NHL repeat-containing protein 2
NID1_MOUSE	19	139302	1	1	1	Nidogen-1

NIF3L_MOUSE	31	42176	2	2	2	NIF3-like protein 1
NIN_MOUSE	23	245791	2	1	1	Ninein
NIPS2_MOUSE	1700	33083	150	12	12	Protein NipSnap homolog 2
NIT1_MOUSE	62	36423	4	1	1	Deaminated glutathione amidase
NIT2_MOUSE	747	30825	46	5	5	Omega-amidase NIT2
NLTP_MOUSE	28	59715	2	2	2	Non-specific lipid-transfer protein
NNRD_MOUSE	159	37093	18	3	3	ATP-dependent (S)-NAD(P)H-hydrate dehydratase
NNRE_MOUSE	71	31295	11	3	3	NAD(P)H-hydrate epimerase
NOL3_MOUSE	1985	24781	82	7	7	Nucleolar protein 3
NP1L1_MOUSE	282	45602	21	2	1	Nucleosome assembly protein 1-like 1
NP1L4_MOUSE	559	42824	37	5	4	Nucleosome assembly protein 1-like 4
NPM_MOUSE	40	32711	3	1	1	Nucleophosmin
NQO1_MOUSE	64	30997	3	2	2	NAD(P)H dehydrogenase [quinone] 1
NQO2_MOUSE	600	26459	42	7	7	Ribosyldihydronicotinamide dehydrogenase [quinone]
NR1D1_MOUSE	23	67615	2	1	1	Nuclear receptor subfamily 1 group D member 1
NRBP_MOUSE	23	60398	1	1	1	Nuclear receptor-binding protein
NRK_MOUSE	28	164515	1	1	1	Nik-related protein kinase
NRK2_MOUSE	43	22531	3	1	1	Nicotinamide riboside kinase 2
NSF1C_MOUSE	1078	40685	93	13	13	NSFL1 cofactor p47
NT5C_MOUSE	17	23290	1	1	1	5'(3')-deoxyribonucleotidase, cytosolic type
NTF2_MOUSE	371	14640	38	5	5	Nuclear transport factor 2
NU5M_MOUSE	188	68771	9	1	1	NADH-ubiquinone oxidoreductase chain 5
NUAK1_MOUSE	17	74129	1	1	1	NUAK family SNF1-like kinase 1
NUCG_MOUSE	35	32285	3	2	2	Endonuclease G, mitochondrial
NUCL_MOUSE	30	76734	2	2	2	Nucleolin
NUD13_MOUSE	38	39682	4	1	1	Nucleoside diphosphate-linked moiety X motif 13
NUD14_MOUSE	25	24656	1	1	1	Uridine diphosphate glucose pyrophosphatase
NUD16_MOUSE	65	21926	7	2	2	U8 snoRNA-decapping enzyme
NUDT3_MOUSE	29	19246	1	1	1	Diphosphoinositol polyphosphate phosphohydrolase 1
NXN_MOUSE	34	48713	3	2	2	Nucleoredoxin
OAT_MOUSE	3006	48723	139	9	9	Ornithine aminotransferase, mitochondrial
OBSCN_MOUSE	854	979281	84	21	21	Obscurin
OCAD1_MOUSE	21	27821	1	1	1	OCIA domain-containing protein 1
OCTC_MOUSE	357	71131	25	3	3	Peroxisomal carnitine O-octanoyltransferase
ODO1_MOUSE	13630	117572	675	41	41	2-oxoglutarate dehydrogenase, mitochondrial
ODO2_MOUSE	4964	49306	243	11	11	Dihydrolipoyllysine-residue succinyltransferase component of 2-oxoglutarate dehydrogenase complex, mitochondrial
ODP2_MOUSE	3768	68469	211	16	16	Dihydrolipoyllysine-residue acetyltransferase component of pyruvate dehydrogenase complex, mitochondrial
ODPA_MOUSE	5552	43888	275	15	15	Pyruvate dehydrogenase E1 component subunit alpha, somatic form, mitochondrial
ODPB_MOUSE	4060	39254	269	12	12	Pyruvate dehydrogenase E1 component subunit beta, mitochondrial
ODPX_MOUSE	389	54250	32	7	7	Pyruvate dehydrogenase protein X component, mitochondrial
OLA1_MOUSE	3324	44929	184	14	14	Obg-like ATPase 1
OPLA_MOUSE	322	138950	22	6	6	5-oxoprolinase
ORN_MOUSE	70	26950	5	1	1	Oligoribonuclease, mitochondrial
OSBP1_MOUSE	57	89596	8	3	3	Oxysterol-binding protein 1
OSGEP_MOUSE	101	36790	5	1	1	Probable tRNA N6-adenosine threonylcarbamoyltransferase
OTUB1_MOUSE	2105	31478	125	8	8	Ubiquitin thioesterase OTUB1
OXR1_MOUSE	17	96309	1	1	1	Oxidation resistance protein 1
OXSR1_MOUSE	1058	58462	51	7	7	Serine/threonine-protein kinase R1
P20D2_MOUSE	36	46852	2	1	1	Peptidase M20 domain-containing protein 2
PA1B2_MOUSE	1416	25736	75	3	3	Platelet-activating factor acetylhydrolase IB subunit beta
PABP1_MOUSE	106	70854	7	2	2	Polyadenylate-binding protein 1
PACN3_MOUSE	25	48897	1	1	1	Protein kinase C and casein kinase II substrate protein 3
PADI2_MOUSE	4122	76942	209	16	16	Protein-arginine deiminase type-2
PALMD_MOUSE	61	62833	14	1	1	Palmdelphin
PAN2_MOUSE	35	136650	2	2	2	PAN2-PAN3 deadenylation complex catalytic subunit Pan2
PANK4_MOUSE	120	92433	8	3	3	Pantothenate kinase 4
PAOX_MOUSE	20	55868	1	1	1	Peroxisomal N(1)-acetyl-spermine/spermidine oxidase
PAQR9_MOUSE	39	43568	5	1	1	Membrane progesterone receptor epsilon
PARK7_MOUSE	12404	20236	601	17	17	Protein/nucleic acid deglycase DJ-1
PARP9_MOUSE	33	97566	2	1	1	Poly [ADP-ribose] polymerase 9
PARVB_MOUSE	145	41700	11	2	2	Beta-parvin
PBIP1_MOUSE	51	81514	4	1	1	Pre-B-cell leukemia transcription factor-interacting protein 1
PCBP1_MOUSE	176	37987	19	6	4	Poly(rC)-binding protein 1
PCBP2_MOUSE	99	38597	12	4	2	Poly(rC)-binding protein 2
PCCA_MOUSE	151	80498	15	5	5	Propionyl-CoA carboxylase alpha chain, mitochondrial
PCCB_MOUSE	313	58999	18	7	7	Propionyl-CoA carboxylase beta chain, mitochondrial
PCD19_MOUSE	31	127154	1	1	1	Protocadherin-19
PCLO_MOUSE	63	552378	5	1	1	Protein piccolo
PCM1_MOUSE	19	230131	2	1	1	Pericentriolar material 1 protein
PCY2_MOUSE	22	45663	1	1	1	Ethanolamine-phosphate cytidylyltransferase
PDC6I_MOUSE	277	96534	21	11	11	Programmed cell death 6-interacting protein
PDCD5_MOUSE	178	14266	20	4	4	Programmed cell death protein 5
PDCD6_MOUSE	49	21911	4	1	1	Programmed cell death protein 6
PDIA1_MOUSE	7164	57422	400	25	25	Protein disulfide-isomerase
PDIA3_MOUSE	1399	57099	94	13	13	Protein disulfide-isomerase A3
PDIA6_MOUSE	1046	48469	59	8	8	Protein disulfide-isomerase A6
PDK2_MOUSE	1374	46239	71	8	8	[Pyruvate dehydrogenase (acetyl-transferring)] kinase isozyme 2, mitochondrial
PDK4_MOUSE	338	46909	28	4	4	[Pyruvate dehydrogenase (acetyl-transferring)] kinase isozyme 4, mitochondrial
PDLI3_MOUSE	5383	34734	240	11	11	PDZ and LIM domain protein 3
PDLI5_MOUSE	838	64514	67	6	6	PDZ and LIM domain protein 5
PDLI7_MOUSE	526	51170	40	4	4	PDZ and LIM domain protein 7
PDP1_MOUSE	104	61712	9	4	4	[Pyruvate dehydrogenase [acetyl-transferring]]-phosphatase 1, mitochondrial
PDPR_MOUSE	210	100079	18	2	2	Pyruvate dehydrogenase phosphatase regulatory subunit, mitochondrial

PDXK_MOUSE	188	35278	16	3	3	Pyridoxal kinase
PEAK1_MOUSE	17	193088	1	1	1	Pseudopodium-enriched atypical kinase 1
PEBP1_MOUSE	9095	20988	416	11	11	Phosphatidylethanolamine-binding protein 1
PEDF_MOUSE	214	46262	13	5	5	Pigment epithelium-derived factor
PEPD_MOUSE	766	55962	62	11	11	Xaa-Pro dipeptidase
PEPL1_MOUSE	252	56817	19	2	2	Probable aminopeptidase NPEPL1
PEX10_MOUSE	96	37704	7	1	1	Peroxisome biogenesis factor 10
PEX6_MOUSE	35	105681	1	1	1	Peroxisome assembly factor 2
PFD3_MOUSE	235	22592	9	2	2	Prefoldin subunit 3
PFD5_MOUSE	118	17402	7	1	1	Prefoldin subunit 5
PFKAL_MOUSE	6821	86104	294	5	1	ATP-dependent 6-phosphofructokinase, liver type
PFKAM_MOUSE	75686	86070	2871	43	39	ATP-dependent 6-phosphofructokinase, muscle type
PGAM1_MOUSE	5513	28928	240	9	4	Phosphoglycerate mutase 1
PGAM2_MOUSE	43092	28980	1980	25	20	Phosphoglycerate mutase 2
PGFS_MOUSE	400	22055	36	5	5	Prostamide/prostaglandin F synthase
PGK1_MOUSE	54777	44921	2382	34	27	Phosphoglycerate kinase 1
PGK2_MOUSE	10410	45223	441	9	2	Phosphoglycerate kinase 2
PGM1_MOUSE	57797	61665	2419	44	44	Phosphoglucomutase-1
PGM5_MOUSE	357	62751	53	2	2	Phosphoglucomutase-like protein 5
PGP_MOUSE	2357	34975	131	11	11	Glycerol-3-phosphate phosphatase
PGRP2_MOUSE	48	58184	1	1	1	N-acetylmuramoyl-L-alanine amidase
PGS1_MOUSE	99	42069	5	1	1	Biglycan
PGS2_MOUSE	699	40126	76	5	5	Decorin
PGTA_MOUSE	115	66203	13	3	3	Geranylgeranyl transferase type-2 subunit alpha
PHB_MOUSE	91	29859	4	1	1	Prohibitin
PHB2_MOUSE	25	33276	1	1	1	Prohibitin-2
PHF8_MOUSE	25	114508	1	1	1	Histone lysine demethylase PHF8
PHKG1_MOUSE	758	45387	74	8	8	Phosphorylase b kinase gamma catalytic chain, skeletal muscle/heart isoform
PHOP1_MOUSE	30	30348	4	3	3	Phosphoethanolamine/phosphocholine phosphatase
PHP14_MOUSE	686	14159	64	5	5	14 kDa phosphohistidine phosphatase
PICAL_MOUSE	26	71669	2	1	1	Phosphatidylinositol-binding clathrin assembly protein
PIMT_MOUSE	838	24676	67	7	7	Protein-L-isoaspartate(D-aspartate) O-methyltransferase
PIN1_MOUSE	45	18473	3	1	1	Peptidyl-prolyl cis-trans isomerase NIMA-interacting 1
PIPNA_MOUSE	864	32101	35	3	3	Phosphatidylinositol transfer protein alpha isoform
PIPNB_MOUSE	47	31752	4	1	1	Phosphatidylinositol transfer protein beta isoform
PK3CA_MOUSE	18	126099	1	1	1	Phosphatidylinositol 4,5-bisphosphate 3-kinase catalytic subunit alpha isoform
PKHA1_MOUSE	18	43743	1	1	1	Pleckstrin homology domain-containing family A member 1
PKHH3_MOUSE	24	86872	1	1	1	Pleckstrin homology domain-containing family H member 3
PLAP_MOUSE	36	88591	2	1	1	Phospholipase A-2-activating protein
PLCD4_MOUSE	256	93547	11	1	1	1-phosphatidylinositol 4,5-bisphosphate phosphodiesterase delta-4
PLEC_MOUSE	818	535800	42	10	10	Plectin
PLIN4_MOUSE	459	140297	30	6	6	Perilipin-4
PLMN_MOUSE	596	93486	41	9	9	Plasminogen
PLPHP_MOUSE	453	30201	45	4	4	Pyridoxal phosphate homeostasis protein
PLSL_MOUSE	65	70732	6	2	2	Plastin-2
PLXA4_MOUSE	24	215787	1	1	1	Plexin-A4
PMGE_MOUSE	872	30131	66	7	7	Bisphosphoglycerate mutase
PMM2_MOUSE	22	27981	1	1	1	Phosphomannomutase 2
PNCB_MOUSE	18	58799	1	1	1	Nicotinate phosphoribosyltransferase
PNPH_MOUSE	124	32541	10	5	5	Purine nucleoside phosphorylase
PNPO_MOUSE	23	30437	1	1	1	Pyridoxine-5'-phosphate oxidase
PP14B_MOUSE	58	16061	2	1	1	Protein phosphatase 1 regulatory subunit 14B
PP1A_MOUSE	944	38257	60	6	1	Serine/threonine-protein phosphatase PP1-alpha catalytic subunit
PP1B_MOUSE	1009	37961	68	8	3	Serine/threonine-protein phosphatase PP1-beta catalytic subunit
PP1R7_MOUSE	890	41380	57	9	9	Protein phosphatase 1 regulatory subunit 7
PP2AA_MOUSE	634	36156	51	7	7	Serine/threonine-protein phosphatase 2A catalytic subunit alpha isoform
PP2BA_MOUSE	445	59291	40	3	1	Serine/threonine-protein phosphatase 2B catalytic subunit alpha isoform
PP2BB_MOUSE	493	59820	40	3	1	Serine/threonine-protein phosphatase 2B catalytic subunit beta isoform
PP6R3_MOUSE	59	95449	4	1	1	Serine/threonine-protein phosphatase 6 regulatory subunit 3
PPA6_MOUSE	38	47994	2	2	2	Lysophosphatidic acid phosphatase type 6
PPAC_MOUSE	2040	18636	93	6	6	Low molecular weight phosphotyrosine protein phosphatase
PPCE_MOUSE	2070	81669	134	15	15	Prolyl endopeptidase
PPCS_MOUSE	33	33830	2	1	1	Phosphopantothenate--cysteine ligase
PPIA_MOUSE	4231	18131	203	11	11	Peptidyl-prolyl cis-trans isomerase A
PPIB_MOUSE	116	23699	14	3	3	Peptidyl-prolyl cis-trans isomerase B
PPID_MOUSE	91	41116	7	1	1	Peptidyl-prolyl cis-trans isomerase D
PPM1A_MOUSE	37	42976	3	2	2	Protein phosphatase 1A
PPM1F_MOUSE	226	49922	12	1	1	Protein phosphatase 1F
PPME1_MOUSE	209	42628	18	1	1	Protein phosphatase methylesterase 1
PPP5_MOUSE	35	57467	3	3	3	Serine/threonine-protein phosphatase 5
PPP6_MOUSE	18	35821	1	1	1	Serine/threonine-protein phosphatase 6 catalytic subunit
PRAF3_MOUSE	38	21543	1	1	1	PRA1 family protein 3
PRDX1_MOUSE	3980	22390	330	15	15	Peroxiredoxin-1
PRDX2_MOUSE	8875	21936	353	12	12	Peroxiredoxin-2
PRDX3_MOUSE	6054	28337	347	11	11	Thioredoxin-dependent peroxide reductase, mitochondrial
PRDX5_MOUSE	7465	22226	284	11	11	Peroxiredoxin-5, mitochondrial
PRDX6_MOUSE	9702	24969	428	13	13	Peroxiredoxin-6
PRELP_MOUSE	1523	43607	71	7	7	Prolargin
PREP_MOUSE	24	118438	1	1	1	Presequence protease, mitochondrial
PRKN_MOUSE	18	53523	1	1	1	E3 ubiquitin-protein ligase parkin
PROF1_MOUSE	4120	15119	246	6	6	Profilin-1
PROF2_MOUSE	950	15364	81	5	5	Profilin-2
PRPS1_MOUSE	12417	35325	399	15	15	Ribose-phosphate pyrophosphokinase 1

PRR33_MOUSE	49	28673	4	1	1	Proline-rich protein 33
PRS10_MOUSE	307	44430	24	7	7	26S proteasome regulatory subunit 10B
PRS4_MOUSE	62	49325	4	3	3	26S proteasome regulatory subunit 4
PRS6A_MOUSE	83	49803	9	4	4	26S proteasome regulatory subunit 6A
PRS6B_MOUSE	30	47493	1	1	1	26S proteasome regulatory subunit 6B
PRS7_MOUSE	163	49016	14	4	4	26S proteasome regulatory subunit 7
PRS8_MOUSE	322	45768	28	7	7	26S proteasome regulatory subunit 8
PRUN1_MOUSE	364	50892	24	4	4	Exopolyphosphatase PRUNE1
PRVA_MOUSE	20324	11923	779	16	16	Parvalbumin alpha
PSA_MOUSE	2825	103944	213	27	27	Puromycin-sensitive aminopeptidase
PSA1_MOUSE	466	29813	73	7	7	Proteasome subunit alpha type-1
PSA2_MOUSE	1364	26024	67	8	8	Proteasome subunit alpha type-2
PSA3_MOUSE	534	28615	31	6	6	Proteasome subunit alpha type-3
PSA4_MOUSE	750	29737	50	7	7	Proteasome subunit alpha type-4
PSA5_MOUSE	174	26565	12	5	5	Proteasome subunit alpha type-5
PSA6_MOUSE	734	27811	42	5	5	Proteasome subunit alpha type-6
PSA7_MOUSE	731	28009	42	5	5	Proteasome subunit alpha type-7
PSB1_MOUSE	589	26583	42	6	6	Proteasome subunit beta type-1
PSB2_MOUSE	676	23063	23	2	2	Proteasome subunit beta type-2
PSB3_MOUSE	942	23235	25	2	2	Proteasome subunit beta type-3
PSB4_MOUSE	84	29211	9	2	2	Proteasome subunit beta type-4
PSB5_MOUSE	309	28685	20	8	8	Proteasome subunit beta type-5
PSB6_MOUSE	361	25591	34	4	4	Proteasome subunit beta type-6
PSB7_MOUSE	82	30214	7	2	2	Proteasome subunit beta type-7
PSD11_MOUSE	267	47692	27	10	10	26S proteasome non-ATPase regulatory subunit 11
PSD12_MOUSE	495	53261	27	5	5	26S proteasome non-ATPase regulatory subunit 12
PSD13_MOUSE	478	43124	30	6	6	26S proteasome non-ATPase regulatory subunit 13
PSDE_MOUSE	146	34726	20	4	4	26S proteasome non-ATPase regulatory subunit 14
PSMD1_MOUSE	710	106689	45	11	11	26S proteasome non-ATPase regulatory subunit 1
PSMD2_MOUSE	788	100937	58	12	12	26S proteasome non-ATPase regulatory subunit 2
PSMD3_MOUSE	716	60794	33	6	6	26S proteasome non-ATPase regulatory subunit 3
PSMD5_MOUSE	249	56336	16	3	3	26S proteasome non-ATPase regulatory subunit 5
PSMD6_MOUSE	861	45792	33	5	5	26S proteasome non-ATPase regulatory subunit 6
PSMD7_MOUSE	903	36574	30	5	5	26S proteasome non-ATPase regulatory subunit 7
PSMD8_MOUSE	51	40190	4	3	3	26S proteasome non-ATPase regulatory subunit 8
PSME1_MOUSE	721	28826	44	3	3	Proteasome activator complex subunit 1
PSME2_MOUSE	431	27268	38	5	5	Proteasome activator complex subunit 2
PSME4_MOUSE	59	213456	4	1	1	Proteasome activator complex subunit 4
PSYR_MOUSE	70	40103	21	1	1	Psychosine receptor
PTER_MOUSE	163	39535	17	7	7	Phosphotriesterase-related protein
PTG3L_MOUSE	48	15814	4	1	1	Putative protein PTGES3L
PTGR2_MOUSE	465	38447	33	4	4	Prostaglandin reductase 2
PTGR3_MOUSE	46	40788	3	2	2	Prostaglandin reductase-3
PTN11_MOUSE	280	68988	16	4	4	Tyrosine-protein phosphatase non-receptor type 11
PTPA_MOUSE	267	36972	17	4	4	Serine/threonine-protein phosphatase 2A activator
PTPRU_MOUSE	26	164329	3	1	1	Receptor-type tyrosine-protein phosphatase U
PUR2_MOUSE	1993	108633	118	14	14	Trifunctional purine biosynthetic protein adenosine-3
PUR6_MOUSE	1603	47717	82	11	11	Multifunctional protein ADE2
PUR8_MOUSE	6344	55572	291	18	18	Adenylosuccinate lyase
PUR9_MOUSE	3466	64690	216	22	22	Bifunctional purine biosynthesis protein PURH
PURA_MOUSE	47	34976	2	1	1	Transcriptional activator protein Pur-alpha
PURA1_MOUSE	27535	50508	1296	26	26	Adenylosuccinate synthetase isozyme 1
PX11C_MOUSE	21	27533	1	1	1	Peroxisomal membrane protein 11C
PYC_MOUSE	882	130344	69	14	14	Pyruvate carboxylase, mitochondrial
PYGM_MOUSE	122076	97681	4563	70	70	Glycogen phosphorylase, muscle form
PYRG1_MOUSE	33	67324	3	3	3	CTP synthase 1
PZP_MOUSE	13466	167116	742	47	47	Pregnancy zone protein
QCR1_MOUSE	1799	53446	114	12	12	Cytochrome b-c1 complex subunit 1, mitochondrial
QCR10_MOUSE	29	6534	4	1	1	Cytochrome b-c1 complex subunit 10
QCR2_MOUSE	1905	48262	103	14	14	Cytochrome b-c1 complex subunit 2, mitochondrial
QCR6_MOUSE	74	10713	2	1	1	Cytochrome b-c1 complex subunit 6, mitochondrial
QCR8_MOUSE	258	9762	20	3	3	Cytochrome b-c1 complex subunit 8
QCR9_MOUSE	216	7441	18	2	2	Cytochrome b-c1 complex subunit 9
QOR_MOUSE	592	35531	66	10	10	Quinone oxidoreductase
QSOX1_MOUSE	34	83531	2	2	2	Sulfhydryl oxidase 1
RAB10_MOUSE	1790	22755	76	5	4	Ras-related protein Rab-10
RAB12_MOUSE	330	27653	17	2	1	Ras-related protein Rab-12
RAB5A_MOUSE	133	23812	11	1	1	Ras-related protein Rab-5A
RAB7A_MOUSE	29	23760	1	1	1	Ras-related protein Rab-7a
RAC1_MOUSE	686	21835	79	6	5	Ras-related C3 botulinum toxin substrate 1
RACK1_MOUSE	452	35511	27	7	7	Receptor of activated protein C kinase 1
RAD54_MOUSE	36	85724	1	1	1	DNA repair and recombination protein RAD54-like
RADI_MOUSE	1055	68614	103	17	11	Radixin
RAN_MOUSE	2196	24579	142	7	7	GTP-binding nuclear protein Ran
RAP1A_MOUSE	289	21316	13	1	1	Ras-related protein Rap-1A
RB11B_MOUSE	39	24589	2	2	2	Ras-related protein Rab-11B
RBBP7_MOUSE	64	48102	4	1	1	Histone-binding protein RBBP7
RBBP9_MOUSE	29	21069	2	1	1	Putative hydrolase RBBP9
RBM3_MOUSE	113	16595	12	2	2	RNA-binding protein 3
RBM7_MOUSE	19	30130	1	1	1	RNA-binding protein 7
RBP2_MOUSE	20	344727	1	1	1	E3 SUMO-protein ligase RanBP2
RBSK_MOUSE	23	34610	1	1	1	Ribokinase
RD23A_MOUSE	422	39739	25	3	2	UV excision repair protein RAD23 homolog A

RD23B_MOUSE	465	43543	26	4	3	UV excision repair protein RAD23 homolog B
RFTN1_MOUSE	22	61955	2	1	1	Raftlin
RHOA_MOUSE	552	22110	48	5	5	Transforming protein RhoA
RIC1_MOUSE	17	160495	1	1	1	RAB6A-GEF complex partner protein 1
RIFK_MOUSE	131	17483	10	1	1	Riboflavin kinase
RIMB1_MOUSE	52	202143	6	1	1	Peripheral-type benzodiazepine receptor-associated protein 1
RINI_MOUSE	1664	51495	116	14	14	Ribonuclease inhibitor
RL10L_MOUSE	35	24998	3	1	1	60S ribosomal protein L10-like
RL12_MOUSE	29	17965	1	1	1	60S ribosomal protein L12
RL14_MOUSE	46	23663	2	1	1	60S ribosomal protein L14
RL17_MOUSE	28	21637	1	1	1	60S ribosomal protein L17
RL18A_MOUSE	29	21004	1	1	1	60S ribosomal protein L18a
RL19_MOUSE	22	23565	1	1	1	60S ribosomal protein L19
RL22_MOUSE	26	14807	1	1	1	60S ribosomal protein L22
RL23_MOUSE	27	14970	1	1	1	60S ribosomal protein L23
RL26_MOUSE	33	17248	2	1	1	60S ribosomal protein L26
RL27_MOUSE	31	15788	3	2	2	60S ribosomal protein L27
RL27A_MOUSE	49	16709	4	1	1	60S ribosomal protein L27a
RL30_MOUSE	37	12947	2	1	1	60S ribosomal protein L30
RL31_MOUSE	49	14454	2	1	1	60S ribosomal protein L31
RL4_MOUSE	33	47409	3	2	2	60S ribosomal protein L4
RL7_MOUSE	128	31457	10	2	2	60S ribosomal protein L7
RL7A_MOUSE	24	30129	2	1	1	60S ribosomal protein L7a
RL8_MOUSE	147	28235	6	1	1	60S ribosomal protein L8
RL9_MOUSE	24	21982	1	1	1	60S ribosomal protein L9
RM21_MOUSE	25	23466	3	1	1	39S ribosomal protein L21, mitochondrial
RMD1_MOUSE	38	35263	1	1	1	Regulator of microtubule dynamics protein 1
RN123_MOUSE	488	150040	20	2	2	E3 ubiquitin-protein ligase RNF123
RNAS4_MOUSE	21	17470	1	1	1	Ribonuclease 4
RNF14_MOUSE	62	56601	3	1	1	E3 ubiquitin-protein ligase RNF14
RNF31_MOUSE	107	122374	11	1	1	E3 ubiquitin-protein ligase RNF31
ROA2_MOUSE	222	37437	13	4	4	Heterogeneous nuclear ribonucleoproteins A2/B1
ROA3_MOUSE	47	39856	3	1	1	Heterogeneous nuclear ribonucleoprotein A3
ROAA_MOUSE	22	30926	2	1	1	Heterogeneous nuclear ribonucleoprotein A/B
ROBO1_MOUSE	17	177552	1	1	1	Roundabout homolog 1
RP3A_MOUSE	21	76297	1	1	1	Rabphilin-3A
RPIA_MOUSE	116	32545	6	2	2	Ribose-5-phosphate isomerase
RRAGA_MOUSE	238	36942	14	1	1	Ras-related GTP-binding protein A
RRAGC_MOUSE	28	44492	1	1	1	Ras-related GTP-binding protein C
RS10_MOUSE	490	18904	22	2	2	40S ribosomal protein S10
RS11_MOUSE	37	18590	5	2	2	40S ribosomal protein S11
RS12_MOUSE	32	14858	1	1	1	40S ribosomal protein S12
RS13_MOUSE	346	17212	33	5	5	40S ribosomal protein S13
RS14_MOUSE	132	16434	11	2	2	40S ribosomal protein S14
RS15_MOUSE	80	17029	3	1	1	40S ribosomal protein S15
RS15A_MOUSE	71	14944	6	1	1	40S ribosomal protein S15a
RS16_MOUSE	218	16549	15	3	3	40S ribosomal protein S16
RS17_MOUSE	18	15571	1	1	1	40S ribosomal protein S17
RS18_MOUSE	163	17708	11	3	3	40S ribosomal protein S18
RS19_MOUSE	267	16076	19	3	3	40S ribosomal protein S19
RS2_MOUSE	370	31497	19	4	4	40S ribosomal protein S2
RS20_MOUSE	255	13478	21	3	3	40S ribosomal protein S20
RS21_MOUSE	21	9250	1	1	1	40S ribosomal protein S21
RS23_MOUSE	32	15969	1	1	1	40S ribosomal protein S23
RS24_MOUSE	236	15413	10	1	1	40S ribosomal protein S24
RS25_MOUSE	162	13791	17	5	5	40S ribosomal protein S25
RS26_MOUSE	62	13292	3	1	1	40S ribosomal protein S26
RS27A_MOUSE	1492	18282	82	3	3	Ubiquitin-40S ribosomal protein S27a
RS28_MOUSE	41	7893	2	1	1	40S ribosomal protein S28
RS3_MOUSE	416	26828	31	9	9	40S ribosomal protein S3
RS3A_MOUSE	101	30094	12	7	7	40S ribosomal protein S3a
RS4X_MOUSE	202	29807	14	4	4	40S ribosomal protein S4, X isoform
RS5_MOUSE	73	23046	6	2	2	40S ribosomal protein S5
RS6_MOUSE	217	28834	13	2	2	40S ribosomal protein S6
RS7_MOUSE	366	22113	27	3	3	40S ribosomal protein S7
RS8_MOUSE	105	24475	9	3	3	40S ribosomal protein S8
RS9_MOUSE	73	22635	8	4	4	40S ribosomal protein S9
RSCA1_MOUSE	35	62125	1	1	1	Regulatory solute carrier protein family 1 member 1
RSPO1_MOUSE	22	30624	1	1	1	R-spondin-1
RSSA_MOUSE	1444	32931	73	7	7	40S ribosomal protein SA
RSU1_MOUSE	703	31531	63	5	5	Ras suppressor protein 1
RT30_MOUSE	23	50364	1	1	1	28S ribosomal protein S30, mitochondrial
RTCA_MOUSE	46	39686	3	2	2	RNA 3'-terminal phosphate cyclase
RTCB_MOUSE	21	55727	1	1	1	tRNA-splicing ligase RtcB homolog
RTN2_MOUSE	1317	51543	60	6	6	Reticulon-2
RTN4_MOUSE	93	127048	11	3	3	Reticulon-4
RTRAF_MOUSE	320	28249	12	1	1	RNA transcription, translation and transport factor protein
RUFY1_MOUSE	34	81466	3	1	1	RUN and FYVE domain-containing protein 1
RYR1_MOUSE	571	570381	45	11	11	Ryanodine receptor 1
S10A1_MOUSE	1253	10555	47	2	2	Protein S100-A1
S10A4_MOUSE	27	11942	3	1	1	Protein S100-A4
S10A6_MOUSE	280	10101	19	4	4	Protein S100-A6
SAE2_MOUSE	57	71209	4	1	1	SUMO-activating enzyme subunit 2

SAHH_MOUSE	2855	48170	172	13	13	Adenosylhomocysteinase
SAMP_MOUSE	35	26401	2	2	2	Serum amyloid P-component
SAP_MOUSE	64	63263	7	2	2	Prosaposin
SAR1B_MOUSE	933	22482	55	4	4	GTP-binding protein SAR1b
SBP1_MOUSE	678	53051	59	10	10	Methanethiol oxidase
SCOT1_MOUSE	4765	56352	220	19	19	Succinyl-CoA:3-ketoacid coenzyme A transferase 1, mitochondrial
SCRN2_MOUSE	1781	47141	78	8	8	Secernin-2
SCRN3_MOUSE	2205	48030	130	11	11	Secernin-3
SDHA_MOUSE	2716	73623	90	14	14	Succinate dehydrogenase [ubiquinone] flavoprotein subunit, mitochondrial
SDHB_MOUSE	300	32591	25	6	6	Succinate dehydrogenase [ubiquinone] iron-sulfur subunit, mitochondrial
SEPT2_MOUSE	23	41727	2	2	2	Septin-2
SEPT7_MOUSE	87	50860	12	2	2	Septin-7
SERHL_MOUSE	40	35573	1	1	1	Serine hydrolase-like protein
SETB1_MOUSE	19	146453	1	1	1	Histone-lysine N-methyltransferase SETDB1
SGT1_MOUSE	76	38420	9	2	2	Protein SGT1 homolog
SGTA_MOUSE	37	34529	1	1	1	Small glutamine-rich tetratricopeptide repeat-containing protein alpha
SH2B1_MOUSE	33	80032	1	1	1	SH2B adapter protein 1
SH3BG_MOUSE	1405	23146	106	7	7	SH3 domain-binding glutamic acid-rich protein
SIAS_MOUSE	185	40455	15	2	2	Sialic acid synthase
SIR2_MOUSE	316	43856	19	2	2	NAD-dependent protein deacetylase sirtuin-2
SKP1_MOUSE	175	18831	8	1	1	S-phase kinase-associated protein 1
SLIRP_MOUSE	39	12654	2	2	2	SRA stem-loop-interacting RNA-binding protein, mitochondrial
SLIT2_MOUSE	17	174717	1	1	1	Slit homolog 2 protein
SMPX_MOUSE	17	9247	1	1	1	Small muscular protein
SMTL2_MOUSE	292	49778	13	2	2	Smoothelin-like protein 2
SMYD1_MOUSE	4807	57428	197	17	17	Histone-lysine N-methyltransferase Smyd1
SMYD2_MOUSE	156	50618	12	1	1	N-lysine methyltransferase SMYD2
SMYD5_MOUSE	25	48319	1	1	1	SET and MYND domain-containing protein 5
SNTA1_MOUSE	272	54088	27	6	6	Alpha-1-syntrophin
SNX12_MOUSE	18	19275	1	1	1	Sorting nexin-12
SNX19_MOUSE	24	111107	1	1	1	Sorting nexin-19
SNX3_MOUSE	75	18803	7	1	1	Sorting nexin-3
SOCS1_MOUSE	22	23985	1	1	1	Suppressor of cytokine signaling 1
SODC_MOUSE	4528	16104	175	8	8	Superoxide dismutase [Cu-Zn]
SODE_MOUSE	20	27717	1	1	1	Extracellular superoxide dismutase [Cu-Zn]
SODM_MOUSE	4196	24816	245	11	11	Superoxide dismutase [Mn], mitochondrial
SOGA1_MOUSE	23	160627	1	1	1	Protein SOGA1
SPA3K_MOUSE	21594	47021	897	24	16	Serine protease inhibitor A3K
SPA3M_MOUSE	7575	47205	354	10	1	Serine protease inhibitor A3M
SPA3N_MOUSE	4229	46859	176	7	3	Serine protease inhibitor A3N
SPB6_MOUSE	12160	42913	648	21	21	Serpin B6
SPCS_MOUSE	203	56032	23	1	1	O-phosphoseryl-tRNA(Sec) selenium transferase
SPI2_MOUSE	199	45861	13	2	1	Serpin I2
SPN90_MOUSE	28	79207	1	1	1	NCK-interacting protein with SH3 domain
SPRE_MOUSE	4376	28208	201	10	10	Sepiapterin reductase
SPRY4_MOUSE	58	23374	7	2	2	SPRY domain-containing protein 4
SPS1_MOUSE	24	43392	2	1	1	Selenide, water dikinase 1
SPSY_MOUSE	1485	41743	107	14	14	Spermine synthase
SPTB1_MOUSE	20	245897	1	1	1	Spectrin beta chain, erythrocytic
SRCA_MOUSE	23254	99522	910	35	35	Sarcalumenin
ST1A1_MOUSE	32	34180	1	1	1	Sulfotransferase 1A1
STA5A_MOUSE	22	91345	1	1	1	Signal transducer and activator of transcription 5A
STAC3_MOUSE	125	41317	17	6	6	SH3 and cysteine-rich domain-containing protein 3
STBD1_MOUSE	20	36219	1	1	1	Starch-binding domain-containing protein 1
STIP1_MOUSE	352	63170	36	7	7	Stress-induced-phosphoprotein 1
STML2_MOUSE	18	38475	1	1	1	Stomatin-like protein 2, mitochondrial
STRAP_MOUSE	98	38703	10	2	2	Serine-threonine kinase receptor-associated protein
STX7_MOUSE	32	29973	1	1	1	Syntaxin-7
SUCA_MOUSE	4905	36474	294	9	9	Succinate--CoA ligase [ADP/GDP-forming] subunit alpha, mitochondrial
SUCB1_MOUSE	13449	50424	618	27	27	Succinate--CoA ligase [ADP-forming] subunit beta, mitochondrial
SUCB2_MOUSE	296	47096	40	9	9	Succinate--CoA ligase [GDP-forming] subunit beta, mitochondrial
SULF2_MOUSE	28	101688	1	1	1	Extracellular sulfatase Sulf-2
SUOX_MOUSE	500	61231	30	3	3	Sulfite oxidase, mitochondrial
SYAC_MOUSE	892	107697	47	10	10	Alanine--tRNA ligase, cytoplasmic
SYDC_MOUSE	706	57567	79	12	12	Aspartate--tRNA ligase, cytoplasmic
SYEP_MOUSE	236	171739	22	7	7	Bifunctional glutamate/proline--tRNA ligase
SYHC_MOUSE	142	57909	12	4	4	Histidine--tRNA ligase, cytoplasmic
SYIC_MOUSE	23	145547	1	1	1	Isoleucine--tRNA ligase, cytoplasmic
SYIM_MOUSE	412	113873	26	6	6	Isoleucine--tRNA ligase, mitochondrial
SYK_MOUSE	148	68253	8	2	2	Lysine--tRNA ligase
SYLM_MOUSE	22	102555	1	1	1	Probable leucine--tRNA ligase, mitochondrial
SYMC_MOUSE	31	102735	1	1	1	Methionine--tRNA ligase, cytoplasmic
SYNC_MOUSE	700	65150	47	8	8	Asparagine--tRNA ligase, cytoplasmic
SYNP2_MOUSE	30	116968	1	1	1	Synaptopodin-2
SYPL2_MOUSE	1267	29605	41	2	2	Synaptophysin-like protein 2
SYQ_MOUSE	66	88533	8	3	3	Glutamine--tRNA ligase
SYRC_MOUSE	92	76423	14	5	5	Arginine--tRNA ligase, cytoplasmic
SYSC_MOUSE	783	58865	72	8	8	Serine--tRNA ligase, cytoplasmic
SYTC_MOUSE	45	84215	2	2	2	Threonine--tRNA ligase, cytoplasmic
SYVC_MOUSE	194	141439	12	2	2	Valine--tRNA ligase
SYWC_MOUSE	525	54893	26	3	3	Tryptophan--tRNA ligase, cytoplasmic
SYYC_MOUSE	88	59410	14	3	3	Tyrosine--tRNA ligase, cytoplasmic
TACO1_MOUSE	124	32750	13	3	3	Translational activator of cytochrome c oxidase 1

TAGL_MOUSE	591	22618	54	7	7	Transgelin
TAGL2_MOUSE	185	22552	18	5	5	Transgelin-2
TALDO_MOUSE	1673	37534	84	8	8	Transaldolase
TBA1A_MOUSE	15498	50788	749	22	7	Tubulin alpha-1A chain
TBA4A_MOUSE	18829	50634	809	22	7	Tubulin alpha-4A chain
TBA8_MOUSE	11100	50704	465	15	4	Tubulin alpha-8 chain
TBB2B_MOUSE	16559	50377	797	22	3	Tubulin beta-2B chain
TBB3_MOUSE	11398	50842	566	16	1	Tubulin beta-3 chain
TBB4B_MOUSE	20510	50255	998	26	5	Tubulin beta-4B chain
TBB5_MOUSE	16885	50095	814	26	4	Tubulin beta-5 chain
TBB6_MOUSE	9127	50514	436	15	3	Tubulin beta-6 chain
TBCB_MOUSE	18	27654	1	1	1	Tubulin-folding cofactor B
TCPA_MOUSE	140	60867	13	6	6	T-complex protein 1 subunit alpha
TCPB_MOUSE	356	57783	28	8	8	T-complex protein 1 subunit beta
TCPD_MOUSE	713	58543	41	6	6	T-complex protein 1 subunit delta
TCPE_MOUSE	131	60042	10	5	5	T-complex protein 1 subunit epsilon
TCPG_MOUSE	113	61162	13	5	5	T-complex protein 1 subunit gamma
TCPH_MOUSE	391	60127	50	8	8	T-complex protein 1 subunit eta
TCPQ_MOUSE	501	60088	39	7	7	T-complex protein 1 subunit theta
TCPZ_MOUSE	1550	58424	44	3	3	T-complex protein 1 subunit zeta
TCTP_MOUSE	4284	19564	201	9	9	Translationally-controlled tumor protein
TDG_MOUSE	20	47249	1	1	1	G/T mismatch-specific thymine DNA glycosylase
TEBP_MOUSE	41	18995	3	2	2	Prostaglandin E synthase 3
TELT_MOUSE	24	19294	2	1	1	Telethonin
TERA_MOUSE	9140	89950	570	42	42	Transitional endoplasmic reticulum ATPase
THIC_MOUSE	30	41727	2	1	1	Acetyl-CoA acetyltransferase, cytosolic
THIKA_MOUSE	57	44382	2	2	2	3-ketoacyl-CoA thiolase A, peroxisomal
THIL_MOUSE	4717	45129	283	17	17	Acetyl-CoA acetyltransferase, mitochondrial
THIM_MOUSE	6847	42260	388	20	20	3-ketoacyl-CoA thiolase, mitochondrial
THIO_MOUSE	1801	12010	81	3	3	Thioredoxin
THNS2_MOUSE	41	54952	5	2	2	Threonine synthase-like 2
THOP1_MOUSE	190	78774	16	6	6	Thimet oligopeptidase
THRB_MOUSE	91	71649	6	4	4	Prothrombin
THTM_MOUSE	4700	33305	286	12	11	3-mercaptopyruvate sulfurtransferase
THTR_MOUSE	280	33673	34	8	7	Thiosulfate sulfurtransferase
TIGAR_MOUSE	1331	29571	82	8	8	Fructose-2,6-bisphosphatase TIGAR
TIPRL_MOUSE	120	31405	7	1	1	TIP41-like protein
TITIN_MOUSE	2696	3933362	196	47	47	Titin
TKT_MOUSE	1378	68272	96	10	10	Transketolase
TLN1_MOUSE	817	271820	44	17	17	Talin-1
TM1L2_MOUSE	318	55970	10	1	1	TOM1-like protein 2
TM38A_MOUSE	566	33686	21	4	4	Trimeric intracellular cation channel type A
TM9S1_MOUSE	37	69454	8	1	1	Transmembrane 9 superfamily member 1
TMM94_MOUSE	19	154378	1	1	1	Transmembrane protein 94
TMOD4_MOUSE	91	39579	8	2	2	Tropomodulin-4
TNNC2_MOUSE	6313	18155	292	10	9	Troponin C, skeletal muscle
TNNI1_MOUSE	103	21856	5	1	1	Troponin I, slow skeletal muscle
TNNI2_MOUSE	4841	21515	228	7	7	Troponin I, fast skeletal muscle
TNNT3_MOUSE	6965	32221	200	11	11	Troponin T, fast skeletal muscle
TOLIP_MOUSE	189	30553	19	4	4	Toll-interacting protein
TOM1_MOUSE	846	54519	48	6	6	Target of Myb protein 1
TPIS_MOUSE	49259	32684	1945	24	24	Triosephosphate isomerase
TPM1_MOUSE	15954	32718	708	23	11	Tropomyosin alpha-1 chain
TPM2_MOUSE	9415	32931	433	20	12	Tropomyosin beta chain
TPM3_MOUSE	4604	33031	243	11	3	Tropomyosin alpha-3 chain
TPP2_MOUSE	39	141330	2	2	2	Tripeptidyl-peptidase 2
TPPP3_MOUSE	79	19125	5	2	2	Tubulin polymerization-promoting protein family member 3
TRAF5_MOUSE	19	65643	1	1	1	TNF receptor-associated factor 5
TRAP1_MOUSE	1290	80501	58	6	6	Heat shock protein 75 kDa, mitochondrial
TRDN_MOUSE	125	77967	11	2	2	Triadin
TRFE_MOUSE	44344	78841	2053	51	50	Serotransferrin
TRI56_MOUSE	27	81173	1	1	1	E3 ubiquitin-protein ligase TRIM56
TRI69_MOUSE	18	58301	1	1	1	E3 ubiquitin-protein ligase TRIM69
TRI72_MOUSE	5407	53695	269	17	17	Tripartite motif-containing protein 72
TRNT1_MOUSE	25	50035	1	1	1	CCA tRNA nucleotidyltransferase 1, mitochondrial
TRXR1_MOUSE	3245	68380	142	10	10	Thioredoxin reductase 1, cytoplasmic
TRXR2_MOUSE	24	57335	1	1	1	Thioredoxin reductase 2, mitochondrial
TRYB1_MOUSE	56	30773	1	1	1	Tryptase
TS101_MOUSE	33	44267	2	1	1	Tumor susceptibility gene 101 protein
TSN_MOUSE	180	26299	18	4	4	Translin
TSN7_MOUSE	35	28153	1	1	1	Tetraspanin-7
TSNAX_MOUSE	41	33020	2	1	1	Translin-associated protein X
TSP4_MOUSE	459	108979	49	15	13	Thrombospondin-4
TTHY_MOUSE	3098	15880	176	6	6	Transthyretin
TTL12_MOUSE	60	74680	4	2	2	Tubulin--tyrosine ligase-like protein 12
TTPAL_MOUSE	23	39267	1	1	1	Alpha-tocopherol transfer protein-like
TWF2_MOUSE	32	39674	2	1	1	Twinfilin-2
TXD17_MOUSE	87	14348	10	2	2	Thioredoxin domain-containing protein 17
TXLNB_MOUSE	48	77730	2	1	1	Beta-taxilin
TXND5_MOUSE	20	47070	1	1	1	Thioredoxin domain-containing protein 5
TXNL1_MOUSE	231	32616	23	3	3	Thioredoxin-like protein 1
UAP1L_MOUSE	1120	57319	37	2	2	UDP-N-acetylhexosamine pyrophosphorylase-like protein 1
UB2D1_MOUSE	487	16819	42	4	3	Ubiquitin-conjugating enzyme E2 D1

UB2D2_MOUSE	870	16953	76	4	1	Ubiquitin-conjugating enzyme E2 D2
UB2D3_MOUSE	871	16904	76	4	1	Ubiquitin-conjugating enzyme E2 D3
UB2FA_MOUSE	52	21382	1	1	1	NEDD8-conjugating enzyme UBE2F
UB2L3_MOUSE	2356	18021	110	6	6	Ubiquitin-conjugating enzyme E2 L3
UB2V1_MOUSE	1572	16458	107	5	5	Ubiquitin-conjugating enzyme E2 variant 1
UBA1_MOUSE	11488	118931	552	33	33	Ubiquitin-like modifier-activating enzyme 1
UBA3_MOUSE	32	52389	4	4	4	NEDD8-activating enzyme E1 catalytic subunit
UBAC1_MOUSE	30	45730	3	3	3	Ubiquitin-associated domain-containing protein 1
UBC12_MOUSE	227	21172	16	4	4	NEDD8-conjugating enzyme Ubc12
UBCP1_MOUSE	27	36927	1	1	1	Ubiquitin-like domain-containing CTD phosphatase 1
UBE2B_MOUSE	24	17359	1	1	1	Ubiquitin-conjugating enzyme E2 B
UBE2K_MOUSE	516	22507	38	4	4	Ubiquitin-conjugating enzyme E2 K
UBE2N_MOUSE	2093	17184	148	9	9	Ubiquitin-conjugating enzyme E2 N
UBP14_MOUSE	1303	56422	73	9	9	Ubiquitin carboxyl-terminal hydrolase 14
UBP15_MOUSE	67	113508	8	1	1	Ubiquitin carboxyl-terminal hydrolase 15
UBP5_MOUSE	1661	96685	109	17	17	Ubiquitin carboxyl-terminal hydrolase 5
UBR4_MOUSE	22	578883	1	1	1	E3 ubiquitin-protein ligase UBR4
UBXN1_MOUSE	129	33666	13	2	2	UBX domain-containing protein 1
UCHL1_MOUSE	497	25165	32	9	9	Ubiquitin carboxyl-terminal hydrolase isozyme L1
UCHL3_MOUSE	18	26306	1	1	1	Ubiquitin carboxyl-terminal hydrolase isozyme L3
UCRI_MOUSE	177	29634	21	6	6	Cytochrome b-c1 complex subunit Rieske, mitochondrial
UFC1_MOUSE	53	19640	6	2	2	Ubiquitin-fold modifier-conjugating enzyme 1
UFL1_MOUSE	19	90035	2	1	1	E3 UFM1-protein ligase 1
UGGG1_MOUSE	19	177007	1	1	1	UDP-glucose:glycoprotein glucosyltransferase 1
UGPA_MOUSE	18679	57115	856	29	29	UTP--glucose-1-phosphate uridylyltransferase
ULA1_MOUSE	172	60692	15	3	3	NEDD8-activating enzyme E1 regulatory subunit
UN45B_MOUSE	902	104884	62	14	14	Protein unc-45 homolog B
USO1_MOUSE	258	108000	19	4	4	General vesicular transport factor p115
USP9X_MOUSE	23	294061	1	1	1	Probable ubiquitin carboxyl-terminal hydrolase FAF-X
VAPB_MOUSE	71	27157	7	2	2	Vesicle-associated membrane protein-associated protein B
VAT1_MOUSE	351	43297	18	2	2	Synaptic vesicle membrane protein VAT-1 homolog
VATA_MOUSE	277	68625	21	5	5	V-type proton ATPase catalytic subunit A
VATB2_MOUSE	60	56857	6	4	4	V-type proton ATPase subunit B, brain isoform
VATE1_MOUSE	125	26198	9	2	2	V-type proton ATPase subunit E 1
VATH_MOUSE	33	56275	1	1	1	V-type proton ATPase subunit H
VDAC1_MOUSE	1725	32502	100	14	14	Voltage-dependent anion-selective channel protein 1
VDAC2_MOUSE	184	32340	15	3	3	Voltage-dependent anion-selective channel protein 2
VDAC3_MOUSE	339	31076	18	5	5	Voltage-dependent anion-selective channel protein 3
VIME_MOUSE	62	53712	4	2	1	Vimentin
VINC_MOUSE	2820	117215	196	34	34	Vinculin
VMA5A_MOUSE	241	87829	30	8	8	von Willebrand factor A domain-containing protein 5A
VPS29_MOUSE	76	20654	5	1	1	Vacuolar protein sorting-associated protein 29
VPS35_MOUSE	178	92453	17	7	7	Vacuolar protein sorting-associated protein 35
VPS36_MOUSE	18	43993	1	1	1	Vacuolar protein-sorting-associated protein 36
VTA1_MOUSE	17	34177	1	1	1	Vacuolar protein sorting-associated protein VTA1 homolog
VTDB_MOUSE	1760	55162	104	18	18	Vitamin D-binding protein
VWA8_MOUSE	27	214370	1	1	1	von Willebrand factor A domain-containing protein 8
WDFY3_MOUSE	38	396707	3	1	1	WD repeat and FYVE domain-containing protein 3
WDR1_MOUSE	1478	67049	126	17	17	WD repeat-containing protein 1
WDR75_MOUSE	104	95402	13	1	1	WD repeat-containing protein 75
WISP3_MOUSE	19	41462	1	1	1	WNT1-inducible-signaling pathway protein 3
XDH_MOUSE	163	148578	10	1	1	Xanthine dehydrogenase/oxidase
XIRP1_MOUSE	224	124096	14	4	4	Xin actin-binding repeat-containing protein 1
XPO1_MOUSE	59	124153	7	2	2	Exportin-1
XPO2_MOUSE	1018	111183	119	18	18	Exportin-2
XPO5_MOUSE	165	139050	21	2	2	Exportin-5
XPP1_MOUSE	163	70231	14	6	6	Xaa-Pro aminopeptidase 1
XXLT1_MOUSE	145	44266	20	1	1	Xyloside xylosyltransferase 1
XYLT1_MOUSE	21	108086	1	1	1	Xylosyltransferase 1
YBOX3_MOUSE	265	38790	18	5	5	Y-box-binding protein 3
ZA2G_MOUSE	104	35538	10	3	3	Zinc-alpha-2-glycoprotein
ZC4H2_MOUSE	22	26570	1	1	1	Zinc finger C4H2 domain-containing protein
ZEB1_MOUSE	21	124327	1	1	1	Zinc finger E-box-binding homeobox 1
ZN618_MOUSE	19	106511	1	1	1	Zinc finger protein 618
**Urea/Thiourea exstract**						
1433B_MOUSE	1853	28183	106	9	4	14-3-3 protein beta/alpha
1433E_MOUSE	7345	29326	322	16	13	14-3-3 protein epsilon
1433G_MOUSE	9286	28456	409	18	13	14-3-3 protein gamma
1433Z_MOUSE	2681	27925	148	11	8	14-3-3 protein zeta/delta
2AAA_MOUSE	3013	66079	139	15	15	Serine/threonine-protein phosphatase 2A 65 kDa regulatory subunit A alpha isoform
2ABA_MOUSE	189	52173	16	2	2	Serine/threonine-protein phosphatase 2A 55 kDa regulatory subunit B alpha isoform
3HIDH_MOUSE	442	35816	20	2	2	3-hydroxyisobutyrate dehydrogenase, mitochondrial
5NT3A_MOUSE	59	37571	4	2	2	Cytosolic 5'-nucleotidase 3A
68MP_MOUSE	419	6694	28	2	2	6.8 kDa mitochondrial proteolipid
A16L1_MOUSE	23	68586	1	1	1	Autophagy-related protein 16-1
A1AG1_MOUSE	143	23994	5	1	1	Alpha-1-acid glycoprotein 1
A1AT2_MOUSE	3310	46117	171	10	3	Alpha-1-antitrypsin 1-2
A1AT3_MOUSE	4316	45966	226	14	7	Alpha-1-antitrypsin 1-3
A2AP_MOUSE	32	55165	4	1	1	Alpha-2-antiplasmin
AAKG1_MOUSE	411	37668	14	2	2	5'-AMP-activated protein kinase subunit gamma-1
AAKG3_MOUSE	79	54271	6	1	1	5'-AMP-activated protein kinase subunit gamma-3
AAPK2_MOUSE	140	62610	8	2	2	5'-AMP-activated protein kinase catalytic subunit alpha-2
AATC_MOUSE	6740	46504	356	22	22	Aspartate aminotransferase, cytoplasmic

AATM_MOUSE	20455	47780	873	24	24	Aspartate aminotransferase, mitochondrial
ABCA4_MOUSE	36	262323	4	2	2	Retinal-specific ATP-binding cassette transporter
ABCB5_MOUSE	35	138450	5	1	1	ATP-binding cassette sub-family B member 5
ABCB8_MOUSE	111	78691	4	1	1	ATP-binding cassette sub-family B member 8, mitochondrial
ABCE1_MOUSE	26	68240	1	1	1	ATP-binding cassette sub-family E member 1
ABCF2_MOUSE	35	72306	1	1	1	ATP-binding cassette sub-family F member 2
ABEC2_MOUSE	6681	25872	328	12	12	C->U-editing enzyme APOBEC-2
ABH15_MOUSE	17	52032	1	1	1	Protein ABHD15
ABLM2_MOUSE	36	69774	2	2	2	Actin-binding LIM protein 2
ACAD8_MOUSE	20	45789	2	1	1	Isobutyryl-CoA dehydrogenase, mitochondrial
ACAD9_MOUSE	939	69192	70	2	2	Acyl-CoA dehydrogenase family member 9, mitochondrial
ACADL_MOUSE	6086	48277	294	20	20	Long-chain specific acyl-CoA dehydrogenase, mitochondrial
ACADM_MOUSE	4283	46908	212	16	16	Medium-chain specific acyl-CoA dehydrogenase, mitochondrial
ACADS_MOUSE	1114	45146	67	9	9	Short-chain specific acyl-CoA dehydrogenase, mitochondrial
ACADV_MOUSE	18081	71230	732	32	32	Very long-chain specific acyl-CoA dehydrogenase, mitochondrial
ACBP_MOUSE	122	9994	6	1	1	Acyl-CoA-binding protein
ACD10_MOUSE	628	119816	37	6	6	Acyl-CoA dehydrogenase family member 10
ACDSB_MOUSE	1151	48300	33	3	3	Short/branched chain specific acyl-CoA dehydrogenase, mitochondrial
ACHB3_MOUSE	21	53363	2	1	1	Neuronal acetylcholine receptor subunit beta-3
ACO13_MOUSE	235	15287	16	4	4	Acyl-coenzyme A thioesterase 13
ACON_MOUSE	38540	86151	1582	42	42	Aconitate hydratase, mitochondrial
ACOT2_MOUSE	121	49854	8	1	1	Acyl-coenzyme A thioesterase 2, mitochondrial
ACPM_MOUSE	47	17644	3	1	1	Acyl carrier protein, mitochondrial
ACSF3_MOUSE	40	65435	6	1	1	Acyl-CoA synthetase family member 3, mitochondrial
ACSL1_MOUSE	10711	78928	527	31	31	Long-chain-fatty-acid--CoA ligase 1
ACTA_MOUSE	245347	42381	8931	37	4	Actin, aortic smooth muscle
ACTB_MOUSE	78366	42052	3710	27	9	Actin, cytoplasmic 1
ACTBL_MOUSE	38101	42319	1268	14	5	Beta-actin-like protein 2
ACTN1_MOUSE	44317	103631	1726	27	4	Alpha-actinin-1
ACTN2_MOUSE	127571	104339	4804	72	48	Alpha-actinin-2
ACTN3_MOUSE	174624	103605	6267	81	62	Alpha-actinin-3
ACTN4_MOUSE	32572	105368	1344	22	3	Alpha-actinin-4
ACTS_MOUSE	304957	42366	10751	39	5	Actin, alpha skeletal muscle
ACTY_MOUSE	258	42369	23	3	1	Beta-centractin
ACTZ_MOUSE	248	42701	21	3	1	Alpha-centractin
ACYP2_MOUSE	167	11927	12	3	3	Acylphosphatase-2
ADDA_MOUSE	36	80995	1	1	1	Alpha-adducin
ADHX_MOUSE	63	40320	3	3	3	Alcohol dehydrogenase class-3
ADK_MOUSE	25	40466	1	1	1	Adenosine kinase
ADT1_MOUSE	30440	33111	1311	25	25	ADP/ATP translocase 1
AGRF4_MOUSE	18	78479	1	1	1	Adhesion G protein-coupled receptor F4
AGRG6_MOUSE	89	131804	8	1	1	Adhesion G-protein coupled receptor G6
AGRIN_MOUSE	70	215386	19	1	1	Agrin
AIFM1_MOUSE	1854	66952	102	14	14	Apoptosis-inducing factor 1, mitochondrial
AIMP2_MOUSE	74	35640	7	2	2	Aminoacyl tRNA synthase complex-interacting multifunctional protein 2
AKAP9_MOUSE	27	439138	2	1	1	A-kinase anchor protein 9
AL1A1_MOUSE	32	55060	1	1	1	Retinal dehydrogenase 1
AL3A1_MOUSE	82	50848	8	4	4	Aldehyde dehydrogenase, dimeric NADP-preferring
AL4A1_MOUSE	317	62258	21	3	3	Delta-1-pyrroline-5-carboxylate dehydrogenase, mitochondrial
ALBU_MOUSE	51275	70700	2157	41	41	Serum albumin
ALDH2_MOUSE	1812	57015	110	15	15	Aldehyde dehydrogenase, mitochondrial
ALDOA_MOUSE	87523	39787	4119	37	32	Fructose-bisphosphate aldolase A
ALDOC_MOUSE	11799	39769	748	8	3	Fructose-bisphosphate aldolase C
ALDR_MOUSE	4319	36052	203	13	13	Aldose reductase
ALLC_MOUSE	18	46721	1	1	1	Probable allantoicase
AMPD1_MOUSE	15609	86734	770	39	39	AMP deaminase 1
AMPL_MOUSE	43	56505	5	2	2	Cytosol aminopeptidase
AMPN_MOUSE	25	110038	1	1	1	Aminopeptidase N
AMRP_MOUSE	19	42189	1	1	1	Alpha-2-macroglobulin receptor-associated protein
ANGL7_MOUSE	213	39502	11	4	4	Angiopoietin-related protein 7
ANK1_MOUSE	226	205355	16	2	2	Ankyrin-1
ANK2_MOUSE	19	428451	1	1	1	Ankyrin-2
ANK3_MOUSE	24	215412	2	1	1	Ankyrin-3
ANKR2_MOUSE	1289	36855	47	7	7	Ankyrin repeat domain-containing protein 2
ANM6_MOUSE	30	42182	1	1	1	Protein arginine N-methyltransferase 6
ANR44_MOUSE	34	109142	3	1	1	Serine/threonine-protein phosphatase 6 regulatory ankyrin repeat subunit B
ANT3_MOUSE	42	52484	2	2	2	Antithrombin-III
ANX11_MOUSE	1030	54387	57	9	9	Annexin A11
ANXA1_MOUSE	20	38995	1	1	1	Annexin A1
ANXA2_MOUSE	5103	38937	177	15	15	Annexin A2
ANXA5_MOUSE	467	35787	25	6	6	Annexin A5
ANXA6_MOUSE	7862	76294	343	29	29	Annexin A6
ANXA7_MOUSE	57	50178	5	1	1	Annexin A7
AOC3_MOUSE	68	85108	6	1	1	Membrane primary amine oxidase
AOFB_MOUSE	427	59034	17	2	2	Amine oxidase [flavin-containing] B
AP1B1_MOUSE	63	104724	5	3	3	AP-1 complex subunit beta-1
AP2A2_MOUSE	28	104863	1	1	1	AP-2 complex subunit alpha-2
AP2S1_MOUSE	24	17178	1	1	1	AP-2 complex subunit sigma
API5_MOUSE	31	57092	1	1	1	Apoptosis inhibitor 5
APMAP_MOUSE	81	46576	3	1	1	Adipocyte plasma membrane-associated protein
APOA1_MOUSE	3979	30597	137	15	15	Apolipoprotein A-I
APOA2_MOUSE	24	11359	1	1	1	Apolipoprotein A-II
APOA4_MOUSE	90	45001	6	2	2	Apolipoprotein A-IV

APOD_MOUSE	34	21744	2	1	1	Apolipoprotein D
APOE_MOUSE	141	35901	11	4	3	Apolipoprotein E
APT_MOUSE	102	19883	5	2	2	Adenine phosphoribosyltransferase
AQP1_MOUSE	449	29003	21	4	4	Aquaporin-1
ARAP3_MOUSE	76	171573	24	1	1	Arf-GAP with Rho-GAP domain, ANK repeat and PH domain-containing protein 3
ARF1_MOUSE	433	20741	38	7	5	ADP-ribosylation factor 1
ARF4_MOUSE	59	20498	5	3	1	ADP-ribosylation factor 4
ARG33_MOUSE	23	95383	1	1	1	Rho guanine nucleotide exchange factor 33
ARK72_MOUSE	40	41042	2	1	1	Aflatoxin B1 aldehyde reductase member 2
ARL1_MOUSE	37	20513	1	1	1	ADP-ribosylation factor-like protein 1
ARL8B_MOUSE	230	21753	11	3	3	ADP-ribosylation factor-like protein 8B
ARP10_MOUSE	107	46748	10	2	2	Actin-related protein 10
ARP2_MOUSE	29	45017	2	1	1	Actin-related protein 2
ARP3B_MOUSE	636	48062	26	2	2	Actin-related protein 3B
ARPC4_MOUSE	22	19768	1	1	1	Actin-related protein 2/3 complex subunit 4
ARSA_MOUSE	19	54512	1	1	1	Arylsulfatase A
ASGL1_MOUSE	45	34385	1	1	1	Isoaspartyl peptidase/L-asparaginase
ASIC5_MOUSE	21	57342	1	1	1	Acid-sensing ion channel 5
ASPH_MOUSE	388	83390	20	1	1	Aspartyl/asparaginyl beta-hydroxylase
ASPN_MOUSE	9858	42944	485	15	15	Asporin
AT1A1_MOUSE	2225	114221	125	17	4	Sodium/potassium-transporting ATPase subunit alpha-1
AT1A2_MOUSE	4144	113457	250	32	19	Sodium/potassium-transporting ATPase subunit alpha-2
AT1B1_MOUSE	834	35571	36	3	3	Sodium/potassium-transporting ATPase subunit beta-1
AT1B2_MOUSE	40	33722	4	3	3	Sodium/potassium-transporting ATPase subunit beta-2
AT2A1_MOUSE	197952	110723	6535	76	56	Sarcoplasmic/endoplasmic reticulum calcium ATPase 1
AT2A2_MOUSE	60181	116437	2093	35	15	Sarcoplasmic/endoplasmic reticulum calcium ATPase 2
AT5F1_MOUSE	4930	29044	270	13	13	ATP synthase F(0) complex subunit B1, mitochondrial
AT5G1_MOUSE	125	14362	15	1	1	ATP synthase F(0) complex subunit C1, mitochondrial
ATAD1_MOUSE	203	41060	16	2	2	ATPase family AAA domain-containing protein 1
ATAD3_MOUSE	57	66872	2	2	2	ATPase family AAA domain-containing protein 3
ATF6B_MOUSE	18	76473	1	1	1	Cyclic AMP-dependent transcription factor ATF-6 beta
ATLA2_MOUSE	267	66809	24	8	8	Atlastin-2
ATP5E_MOUSE	870	5891	58	2	2	ATP synthase subunit epsilon, mitochondrial
ATP5H_MOUSE	7341	18795	371	10	10	ATP synthase subunit d, mitochondrial
ATP5I_MOUSE	1547	8230	73	4	4	ATP synthase subunit e, mitochondrial
ATP5J_MOUSE	32	12489	1	1	1	ATP synthase-coupling factor 6, mitochondrial
ATP5L_MOUSE	2822	11417	147	2	2	ATP synthase subunit g, mitochondrial
ATP8_MOUSE	148	7761	20	2	2	ATP synthase protein 8
ATPA_MOUSE	59386	59830	1775	38	38	ATP synthase subunit alpha, mitochondrial
ATPB_MOUSE	69839	56265	2495	33	33	ATP synthase subunit beta, mitochondrial
ATPD_MOUSE	1451	17589	58	2	2	ATP synthase subunit delta, mitochondrial
ATPG_MOUSE	8734	32979	412	13	13	ATP synthase subunit gamma, mitochondrial
ATPK_MOUSE	2511	10394	83	2	2	ATP synthase subunit f, mitochondrial
ATPO_MOUSE	16945	23406	523	13	13	ATP synthase subunit O, mitochondrial
ATX2L_MOUSE	39	110865	5	1	1	Ataxin-2-like protein
AUHM_MOUSE	250	33659	21	2	2	Methylglutaconyl-CoA hydratase, mitochondrial
B2L13_MOUSE	321	46862	10	2	2	Bcl-2-like protein 13
B3AT_MOUSE	100	103412	8	2	2	Band 3 anion transport protein
BAF_MOUSE	497	10324	25	2	2	Barrier-to-autointegration factor
BAG3_MOUSE	339	62050	21	4	4	BAG family molecular chaperone regulator 3
BAP31_MOUSE	17	27996	1	1	1	B-cell receptor-associated protein 31
BASI_MOUSE	503	42874	36	7	7	Basigin
BCAM_MOUSE	18	68426	2	1	1	Basal cell adhesion molecule
BCAR1_MOUSE	18	94455	1	1	1	Breast cancer anti-estrogen resistance protein 1
BCAT2_MOUSE	349	44669	14	2	2	Branched-chain-amino-acid aminotransferase, mitochondrial
BCKD_MOUSE	84	46786	5	2	2	[3-methyl-2-oxobutanoate dehydrogenase [lipoamide]] kinase, mitochondrial
BCS1_MOUSE	37	47547	1	1	1	Mitochondrial chaperone BCS1
BDH_MOUSE	1898	38617	93	9	9	D-beta-hydroxybutyrate dehydrogenase, mitochondrial
BGH3_MOUSE	1292	75177	60	8	8	Transforming growth factor-beta-induced protein ig-h3
BICL1_MOUSE	48	65589	7	1	1	BICD family-like cargo adapter 1
BIN1_MOUSE	7278	64658	302	13	13	Myc box-dependent-interacting protein 1
BIP_MOUSE	7911	72492	387	24	22	Endoplasmic reticulum chaperone BiP
BLVRB_MOUSE	126	22297	3	1	1	Flavin reductase (NADPH)
BRCA1_MOUSE	18	201750	1	1	1	Breast cancer type 1 susceptibility protein homolog
BRPF1_MOUSE	19	138877	1	1	1	Peregrin
C1QBP_MOUSE	805	31336	49	3	3	Complement component 1 Q subcomponent-binding protein, mitochondrial
C1TC_MOUSE	50	101820	3	2	2	C-1-tetrahydrofolate synthase, cytoplasmic
C560_MOUSE	1763	18598	101	5	5	Succinate dehydrogenase cytochrome b560 subunit, mitochondrial
CA087_MOUSE	33	63085	1	1	1	Uncharacterized protein C1orf87 homolog
CA2D1_MOUSE	11259	125692	480	29	29	Voltage-dependent calcium channel subunit alpha-2/delta-1
CAB39_MOUSE	29	39989	2	2	2	Calcium-binding protein 39
CAC1S_MOUSE	1780	215402	98	17	17	Voltage-dependent L-type calcium channel subunit alpha-1S
CACB1_MOUSE	3137	65731	207	13	13	Voltage-dependent L-type calcium channel subunit beta-1
CACP_MOUSE	2213	71250	131	15	15	Carnitine O-acetyltransferase
CAD13_MOUSE	807	78536	50	4	4	Cadherin-13
CADM3_MOUSE	16	43337	1	1	1	Cell adhesion molecule 3
CAH2_MOUSE	27	29129	2	1	1	Carbonic anhydrase 2
CAH3_MOUSE	17226	29633	869	19	19	Carbonic anhydrase 3
CALM1_MOUSE	368	16827	43	5	4	Calmodulin-1
CALR_MOUSE	197	48136	15	3	3	Calreticulin
CALX_MOUSE	244	67635	17	2	2	Calnexin
CAN1_MOUSE	30	82739	2	2	2	Calpain-1 catalytic subunit
CAN3_MOUSE	23	95094	1	1	1	Calpain-3

CAND2_MOUSE	3205	137086	158	15	15	Cullin-associated NEDD8-dissociated protein 2
CAP1_MOUSE	376	51875	25	2	1	Adenylyl cyclase-associated protein 1
CAP2_MOUSE	1205	53114	74	7	6	Adenylyl cyclase-associated protein 2
CAPZB_MOUSE	6461	31611	353	17	17	F-actin-capping protein subunit beta
CAR10_MOUSE	27	115598	1	1	1	Caspase recruitment domain-containing protein 10
CASQ1_MOUSE	39023	46349	1752	21	21	Calsequestrin-1
CASQ2_MOUSE	1706	48203	71	8	8	Calsequestrin-2
CATB_MOUSE	43	38168	5	2	2	Cathepsin B
CATD_MOUSE	449	45381	41	8	8	Cathepsin D
CAV1_MOUSE	896	20697	51	6	5	Caveolin-1
CAV3_MOUSE	51	17860	3	2	1	Caveolin-3
CAVN1_MOUSE	4123	43927	169	10	10	Caveolae-associated protein 1
CAVN2_MOUSE	113	46792	7	1	1	Caveolae-associated protein 2
CAVN4_MOUSE	201	41040	23	5	5	Caveolae-associated protein 4
CAZA2_MOUSE	8628	33118	387	15	15	F-actin-capping protein subunit alpha-2
CBPA3_MOUSE	1992	49044	96	11	11	Mast cell carboxypeptidase A
CBPA4_MOUSE	26	47594	1	1	1	Carboxypeptidase A4
CBR2_MOUSE	188	26056	18	2	2	Carbonyl reductase [NADPH] 2
CBR4_MOUSE	342	25570	22	4	4	Carbonyl reductase family member 4
CBX4_MOUSE	23	60884	2	1	1	E3 SUMO-protein ligase CBX4
CC160_MOUSE	17	37432	1	1	1	Coiled-coil domain-containing protein 160
CC169_MOUSE	27	24739	3	1	1	Coiled-coil domain-containing protein 169
CC88B_MOUSE	159	167363	16	2	2	Coiled-coil domain-containing protein 88B
CCG1_MOUSE	32	25617	1	1	1	Voltage-dependent calcium channel gamma-1 subunit
CCR6_MOUSE	27	42873	1	1	1	C-C chemokine receptor type 6
CD151_MOUSE	28	29083	2	1	1	CD151 antigen
CD36_MOUSE	2571	53234	118	9	9	Platelet glycoprotein 4
CD3G_MOUSE	21	20450	1	1	1	T-cell surface glycoprotein CD3 gamma chain
CD47_MOUSE	214	33589	7	1	1	Leukocyte surface antigen CD47
CD81_MOUSE	73	26481	5	1	1	CD81 antigen
CD9_MOUSE	63	25811	2	1	1	CD9 antigen
CD97_MOUSE	23	92863	1	1	1	CD97 antigen
CD99_MOUSE	24	16829	1	1	1	CD99 antigen
CDC42_MOUSE	446	21587	26	3	2	Cell division control protein 42 homolog
CDIPT_MOUSE	65	23925	5	1	1	CDP-diacylglycerol--inositol 3-phosphatidyltransferase
CE128_MOUSE	29	129109	3	2	2	Centrosomal protein of 128 kDa
CE152_MOUSE	50	198578	5	2	2	Centrosomal protein of 152 kDa
CELR1_MOUSE	23	335168	1	1	1	Cadherin EGF LAG seven-pass G-type receptor 1
CEP85_MOUSE	20	86085	1	1	1	Centrosomal protein of 85 kDa
CERU_MOUSE	91	121872	8	4	4	Ceruloplasmin
CES1D_MOUSE	24	62034	1	1	1	Carboxylesterase 1D
CFA36_MOUSE	113	39746	28	1	1	Cilia- and flagella-associated protein 36
CFA54_MOUSE	46	357696	4	1	1	Cilia- and flagella-associated protein 54
CFA73_MOUSE	17	35706	1	1	1	Cilia- and flagella-associated protein 73
CFAB_MOUSE	18	86319	3	1	1	Complement factor B
CH10_MOUSE	1102	10956	47	4	4	10 kDa heat shock protein, mitochondrial
CH60_MOUSE	9634	61088	388	27	27	60 kDa heat shock protein, mitochondrial
CHERP_MOUSE	163	106501	27	1	1	Calcium homeostasis endoplasmic reticulum protein
CILP1_MOUSE	435	134530	18	3	3	Cartilage intermediate layer protein 1
CILP2_MOUSE	64	128078	9	1	1	Cartilage intermediate layer protein 2
CING_MOUSE	86	136593	12	1	1	Cingulin
CIP4_MOUSE	18	68675	1	1	1	Cdc42-interacting protein 4
CISD1_MOUSE	1870	12260	104	6	6	CDGSH iron-sulfur domain-containing protein 1
CISY_MOUSE	12861	51988	521	20	20	Citrate synthase, mitochondrial
CK086_MOUSE	38	14059	1	1	1	Uncharacterized protein C11orf86 homolog
CK2N2_MOUSE	22	8679	1	1	1	Calcium/calmodulin-dependent protein kinase II inhibitor 2
CLH1_MOUSE	3114	193202	173	27	27	Clathrin heavy chain 1
CLU_MOUSE	24	149457	1	1	1	Clustered mitochondria protein homolog
CLUS_MOUSE	22	52250	1	1	1	Clusterin
CMA1_MOUSE	796	27968	46	6	6	Chymase
CMBL_MOUSE	1641	28226	55	5	5	Carboxymethylenebutenolidase homolog
CMC1_MOUSE	25328	74922	1044	35	35	Calcium-binding mitochondrial carrier protein Aralar1
CMTD1_MOUSE	82	29228	4	1	1	Catechol O-methyltransferase domain-containing protein 1
CMYA5_MOUSE	551	414327	36	14	14	Cardiomyopathy-associated protein 5
CN37_MOUSE	207	47493	6	3	3	2',3'-cyclic-nucleotide 3'-phosphodiesterase
CNBP_MOUSE	46	20833	2	1	1	Cellular nucleic acid-binding protein
CO1A1_MOUSE	4595	138974	259	10	10	Collagen alpha-1(I) chain
CO1A2_MOUSE	5317	129992	234	10	10	Collagen alpha-2(I) chain
CO2A1_MOUSE	94	142970	12	1	1	Collagen alpha-1(II) chain
CO3_MOUSE	278	187905	20	3	3	Complement C3
CO3A1_MOUSE	75	140112	10	2	2	Collagen alpha-1(III) chain
CO4A1_MOUSE	130	161719	12	3	3	Collagen alpha-1(IV) chain
CO6A1_MOUSE	17219	109562	791	30	30	Collagen alpha-1(VI) chain
CO6A2_MOUSE	13188	111406	695	27	27	Collagen alpha-2(VI) chain
CO6A6_MOUSE	840	247937	48	10	10	Collagen alpha-6(VI) chain
COA4_MOUSE	23	10357	1	1	1	Cytochrome c oxidase assembly factor 4 homolog, mitochondrial
COBA1_MOUSE	41	181548	4	1	1	Collagen alpha-1(XI) chain
COBL_MOUSE	38	144518	4	2	2	Protein cordon-bleu
COCA1_MOUSE	7838	341202	488	63	63	Collagen alpha-1(XII) chain
COEA1_MOUSE	40	194089	2	1	1	Collagen alpha-1(XIV) chain
COF1_MOUSE	1386	18776	52	5	3	Cofilin-1
COF2_MOUSE	4628	18812	238	11	9	Cofilin-2
COFA1_MOUSE	168	140841	18	4	4	Collagen alpha-1(XV) chain

COIA1_MOUSE	69	183085	4	1	1	Collagen alpha-1(XVIII) chain
COMP_MOUSE	109	84912	11	4	3	Cartilage oligomeric matrix protein
COMT_MOUSE	172	29695	9	1	1	Catechol O-methyltransferase
COPG1_MOUSE	17	98705	1	1	1	Coatomer subunit gamma-1
COQ3_MOUSE	21	41387	1	1	1	Ubiquinone biosynthesis O-methyltransferase, mitochondrial
COQ5_MOUSE	150	37711	12	2	2	2-methoxy-6-polyprenyl-1,4-benzoquinol methylase, mitochondrial
COQ6_MOUSE	453	51645	18	4	4	Ubiquinone biosynthesis monooxygenase COQ6, mitochondrial
COQ7_MOUSE	342	24197	21	3	3	5-demethoxyubiquinone hydroxylase, mitochondrial
COQ8A_MOUSE	6218	72154	274	14	14	Atypical kinase COQ8A, mitochondrial
COQ9_MOUSE	2557	35232	121	8	8	Ubiquinone biosynthesis protein COQ9, mitochondrial
COR1B_MOUSE	39	54505	1	1	1	Coronin-1B
CORO6_MOUSE	153	53022	26	4	4	Coronin-6
COX1_MOUSE	656	56929	47	2	2	Cytochrome c oxidase subunit 1
COX2_MOUSE	4307	26130	194	5	5	Cytochrome c oxidase subunit 2
COX41_MOUSE	6934	19575	327	9	9	Cytochrome c oxidase subunit 4 isoform 1, mitochondrial
COX5A_MOUSE	2035	16319	111	8	8	Cytochrome c oxidase subunit 5A, mitochondrial
COX5B_MOUSE	1236	14089	107	6	6	Cytochrome c oxidase subunit 5B, mitochondrial
COX6C_MOUSE	752	8464	62	3	3	Cytochrome c oxidase subunit 6C
COX7B_MOUSE	19	9063	1	1	1	Cytochrome c oxidase subunit 7B, mitochondrial
COX7C_MOUSE	242	7328	21	2	2	Cytochrome c oxidase subunit 7C, mitochondrial
COX7R_MOUSE	150	12448	11	2	2	Cytochrome c oxidase subunit 7A-related protein, mitochondrial
COX8B_MOUSE	466	7327	28	2	2	Cytochrome c oxidase subunit 8B, mitochondrial
CP27A_MOUSE	140	60910	13	1	1	Sterol 26-hydroxylase, mitochondrial
CP2R1_MOUSE	30	57733	2	1	1	Vitamin D 25-hydroxylase
CPT1B_MOUSE	3447	89073	191	21	21	Carnitine O-palmitoyltransferase 1, muscle isoform
CPT2_MOUSE	2480	74504	115	13	13	Carnitine O-palmitoyltransferase 2, mitochondrial
CQ10B_MOUSE	241	27764	22	1	1	Coenzyme Q-binding protein COQ10 homolog B, mitochondrial
CREL1_MOUSE	263	47968	11	1	1	Cysteine-rich with EGF-like domain protein 1
CRIP2_MOUSE	74	23510	6	1	1	Cysteine-rich protein 2
CRYAB_MOUSE	2792	20056	142	11	11	Alpha-crystallin B chain
CSAD_MOUSE	18	55737	1	1	1	Cysteine sulfinic acid decarboxylase
CSN1_MOUSE	47	53978	4	1	1	COP9 signalosome complex subunit 1
CSN2_MOUSE	318	51849	18	1	1	COP9 signalosome complex subunit 2
CSN4_MOUSE	118	46541	8	1	1	COP9 signalosome complex subunit 4
CSN7A_MOUSE	81	30377	4	1	1	COP9 signalosome complex subunit 7a
CSRP1_MOUSE	26	21425	1	1	1	Cysteine and glycine-rich protein 1
CSRP3_MOUSE	849	21793	40	4	4	Cysteine and glycine-rich protein 3
CTCFL_MOUSE	31	74772	2	2	2	Transcriptional repressor CTCFL
CTIP_MOUSE	27	102023	1	1	1	DNA endonuclease RBBP8
CTL2_MOUSE	59	81539	8	3	3	Choline transporter-like protein 2
CTNA1_MOUSE	19	100728	1	1	1	Catenin alpha-1
CUL5_MOUSE	464	91486	20	3	3	Cullin-5
CX6A1_MOUSE	35	12344	1	1	1	Cytochrome c oxidase subunit 6A1, mitochondrial
CX6A2_MOUSE	376	10856	30	2	2	Cytochrome c oxidase subunit 6A2, mitochondrial
CX6B1_MOUSE	7455	10293	315	6	6	Cytochrome c oxidase subunit 6B1
CX7A1_MOUSE	1278	9095	69	2	2	Cytochrome c oxidase subunit 7A1, mitochondrial
CX7A2_MOUSE	803	9285	23	3	3	Cytochrome c oxidase subunit 7A2, mitochondrial
CY1_MOUSE	6304	35533	302	11	11	Cytochrome c1, heme protein, mitochondrial
CYB_MOUSE	199	43352	9	1	1	Cytochrome b
CYB5B_MOUSE	291	16365	9	1	1	Cytochrome b5 type B
CYC_MOUSE	3321	11712	124	6	6	Cytochrome c, somatic
D39U1_MOUSE	87	33145	11	3	3	Epimerase family protein SDR39U1
DAG1_MOUSE	22	97186	1	1	1	Dystroglycan
DC1L1_MOUSE	71	56864	4	1	1	Cytoplasmic dynein 1 light intermediate chain 1
DCTN2_MOUSE	92	44204	4	3	3	Dynactin subunit 2
DDB1_MOUSE	220	128027	23	6	6	DNA damage-binding protein 1
DDX1_MOUSE	701	83474	29	5	5	ATP-dependent RNA helicase DDX1
DDX3X_MOUSE	1330	73455	55	8	8	ATP-dependent RNA helicase DDX3X
DDX5_MOUSE	55	69760	3	1	1	Probable ATP-dependent RNA helicase DDX5
DECR_MOUSE	5040	36476	227	10	10	2,4-dienoyl-CoA reductase, mitochondrial
DERM_MOUSE	942	24549	50	3	3	Dermatopontin
DESM_MOUSE	46804	53522	1629	39	34	Desmin
DESP_MOUSE	18	335158	1	1	1	Desmoplakin
DHB8_MOUSE	180	26800	14	4	4	Estradiol 17-beta-dehydrogenase 8
DHDH_MOUSE	572	36620	31	3	3	Trans-1,2-dihydrobenzene-1,2-diol dehydrogenase
DHE3_MOUSE	443	61640	32	7	7	Glutamate dehydrogenase 1, mitochondrial
DHPR_MOUSE	164	25782	13	2	2	Dihydropteridine reductase
DHRS4_MOUSE	39	30094	5	2	2	Dehydrogenase/reductase SDR family member 4
DHSD_MOUSE	462	17288	27	2	2	Succinate dehydrogenase [ubiquinone] cytochrome b small subunit, mitochondrial
DHTK1_MOUSE	18	103470	1	1	1	Probable 2-oxoglutarate dehydrogenase E1 component DHKTD1, mitochondrial
DIAP1_MOUSE	228	140053	13	3	3	Protein diaphanous homolog 1
DICER_MOUSE	238	219420	20	1	1	Endoribonuclease Dicer
DJB11_MOUSE	39	40815	3	1	1	DnaJ homolog subfamily B member 11
DJC11_MOUSE	48	63536	4	2	2	DnaJ homolog subfamily C member 11
DLDH_MOUSE	9431	54751	371	19	19	Dihydrolipoyl dehydrogenase, mitochondrial
DMD_MOUSE	13567	427676	587	64	64	Dystrophin
DNJA2_MOUSE	591	46344	40	7	7	DnaJ homolog subfamily A member 2
DNJA3_MOUSE	282	53095	20	3	3	DnaJ homolog subfamily A member 3, mitochondrial
DNJA4_MOUSE	219	45500	10	1	1	DnaJ homolog subfamily A member 4
DNJC8_MOUSE	25	29794	2	1	1	DnaJ homolog subfamily C member 8
DNM1L_MOUSE	803	83120	40	8	8	Dynamin-1-like protein
DOCK7_MOUSE	23	243167	1	1	1	Dedicator of cytokinesis protein 7
DPEP1_MOUSE	85	46149	4	1	1	Dipeptidase 1

DPOG1_MOUSE	31	137811	1	1	1	DNA polymerase subunit gamma-1
DPYL2_MOUSE	194	62638	11	3	3	Dihydropyrimidinase-related protein 2
DRC6_MOUSE	21	92423	3	1	1	Dynein regulatory complex subunit 6
DRS7C_MOUSE	3877	34958	124	11	11	Dehydrogenase/reductase SDR family member 7C
DTNA_MOUSE	626	84812	30	6	6	Dystrobrevin alpha
DUPD1_MOUSE	23	24405	2	2	2	Dual specificity phosphatase DUPD1
DUS3_MOUSE	667	20687	36	3	3	Dual specificity protein phosphatase 3
DX39B_MOUSE	274	49460	27	3	3	Spliceosome RNA helicase Ddx39b
DYHC1_MOUSE	542	534447	54	9	9	Cytoplasmic dynein 1 heavy chain 1
DYL1_MOUSE	239	10530	20	1	1	Dynein light chain 1, cytoplasmic
DYSF_MOUSE	6701	240040	300	45	45	Dysferlin
E41L2_MOUSE	48	110386	2	1	1	Band 4.1-like protein 2
ECE2_MOUSE	40	86861	1	1	1	Endothelin-converting enzyme 2
ECH1_MOUSE	399	36437	26	5	5	Delta(3,5)-Delta(2,4)-dienoyl-CoA isomerase, mitochondrial
ECHA_MOUSE	32627	83302	1198	40	40	Trifunctional enzyme subunit alpha, mitochondrial
ECHB_MOUSE	13579	51639	548	20	20	Trifunctional enzyme subunit beta, mitochondrial
ECHD2_MOUSE	89	32174	6	1	1	Enoyl-CoA hydratase domain-containing protein 2, mitochondrial
ECHM_MOUSE	787	31853	62	8	8	Enoyl-CoA hydratase, mitochondrial
ECI1_MOUSE	2793	32515	123	6	6	Enoyl-CoA delta isomerase 1, mitochondrial
ECI2_MOUSE	33	43639	3	1	1	Enoyl-CoA delta isomerase 2, mitochondrial
ECM29_MOUSE	178	205398	18	7	7	Proteasome adapter and scaffold protein ECM29
EDAR_MOUSE	26	49657	1	1	1	Tumor necrosis factor receptor superfamily member EDAR
EF1A1_MOUSE	9689	50424	370	14	5	Elongation factor 1-alpha 1
EF1A2_MOUSE	14482	50764	675	21	12	Elongation factor 1-alpha 2
EF1B_MOUSE	33	24849	4	1	1	Elongation factor 1-beta
EF1D_MOUSE	1191	31388	65	7	7	Elongation factor 1-delta
EF1G_MOUSE	1799	50371	148	14	14	Elongation factor 1-gamma
EF2_MOUSE	9105	96222	444	29	29	Elongation factor 2
EFTU_MOUSE	3516	49876	171	13	13	Elongation factor Tu, mitochondrial
EH1L1_MOUSE	272	185634	19	3	3	EH domain-binding protein 1-like protein 1
EHD1_MOUSE	128	60622	7	1	1	EH domain-containing protein 1
EHD2_MOUSE	867	61307	51	9	9	EH domain-containing protein 2
EI2BA_MOUSE	41	34023	2	1	1	Translation initiation factor eIF-2B subunit alpha
EI3JA_MOUSE	22	29440	2	2	2	Eukaryotic translation initiation factor 3 subunit J-A
EIF3A_MOUSE	121	162237	29	4	4	Eukaryotic translation initiation factor 3 subunit A
EIF3C_MOUSE	272	106149	14	1	1	Eukaryotic translation initiation factor 3 subunit C
EIF3D_MOUSE	30	64576	2	1	1	Eukaryotic translation initiation factor 3 subunit D
EIF3E_MOUSE	51	52587	2	1	1	Eukaryotic translation initiation factor 3 subunit E
EIF3F_MOUSE	52	38074	8	2	2	Eukaryotic translation initiation factor 3 subunit F
EIF3L_MOUSE	70	66798	4	2	2	Eukaryotic translation initiation factor 3 subunit L
EIF3M_MOUSE	77	42946	5	1	1	Eukaryotic translation initiation factor 3 subunit M
ELAV1_MOUSE	38	36317	1	1	1	ELAV-like protein 1
ELMO1_MOUSE	18	84623	1	1	1	Engulfment and cell motility protein 1
ELOB_MOUSE	23	13219	2	2	2	Elongin-B
ENOA_MOUSE	62377	47453	2162	23	16	Alpha-enolase
ENOB_MOUSE	95508	47337	3265	32	24	Beta-enolase
ENOG_MOUSE	40315	47609	1483	10	2	Gamma-enolase
ENPL_MOUSE	1496	92703	38	5	3	Endoplasmin
ENPP4_MOUSE	29	51920	3	1	1	Bis(5'-adenosyl)-triphosphatase enpp4
ENTP2_MOUSE	24	54912	2	1	1	Ectonucleoside triphosphate diphosphohydrolase 2
EPDR1_MOUSE	27	25868	2	2	2	Mammalian ependymin-related protein 1
EPHB4_MOUSE	18	110318	1	1	1	Ephrin type-B receptor 4
ERBB3_MOUSE	25	150825	4	1	1	Receptor tyrosine-protein kinase erbB-3
ES1_MOUSE	882	28415	64	10	10	ES1 protein homolog, mitochondrial
ESIP1_MOUSE	18	36248	1	1	1	Epithelial-stromal interaction protein 1
ESTD_MOUSE	946	31870	44	7	7	S-formylglutathione hydrolase
ETFA_MOUSE	8097	35330	329	15	15	Electron transfer flavoprotein subunit alpha, mitochondrial
ETFB_MOUSE	6577	27834	284	12	12	Electron transfer flavoprotein subunit beta
ETFD_MOUSE	1951	68903	138	18	18	Electron transfer flavoprotein-ubiquinone oxidoreductase, mitochondrial
EX3L1_MOUSE	41	81838	8	1	1	Exocyst complex component 3-like protein
EXOC4_MOUSE	86	111273	16	1	1	Exocyst complex component 4
F111A_MOUSE	23	71044	2	1	1	Protein FAM111A
F162A_MOUSE	89	17884	9	2	2	Protein FAM162A
F16B2_MOUSE	20	83316	1	1	1	Protein FAM160B2
F16P2_MOUSE	661	37209	26	5	5	Fructose-1,6-bisphosphatase isozyme 2
F210A_MOUSE	17	31637	1	1	1	Protein FAM210A
FA35A_MOUSE	25	100453	1	1	1	Protein FAM35A
FA49B_MOUSE	22	37038	1	1	1	Protein FAM49B
FA98A_MOUSE	43	55477	3	2	2	Protein FAM98A
FABP4_MOUSE	2233	14755	102	7	7	Fatty acid-binding protein, adipocyte
FABPH_MOUSE	2999	14810	159	7	7	Fatty acid-binding protein, heart
FACE1_MOUSE	73	54985	8	1	1	CAAX prenyl protease 1 homolog
FAHD2_MOUSE	45	35067	3	1	1	Fumarylacetoacetate hydrolase domain-containing protein 2A
FAS_MOUSE	209	274994	11	2	2	Fatty acid synthase
FBF1_MOUSE	25	130400	1	1	1	Fas-binding factor 1
FBN1_MOUSE	115	332668	17	7	7	Fibrillin-1
FBRL_MOUSE	18	34400	2	2	2	rRNA 2'-O-methyltransferase fibrillarin
FBX21_MOUSE	30	72982	3	1	1	F-box only protein 21
FBX42_MOUSE	19	78469	1	1	1	F-box only protein 42
FETUA_MOUSE	610	38100	45	3	3	Alpha-2-HS-glycoprotein
FGF16_MOUSE	66	23877	12	1	1	Fibroblast growth factor 16
FHAD1_MOUSE	48	164538	1	1	1	Forkhead-associated domain-containing protein 1
FHL1_MOUSE	9705	33806	555	16	16	Four and a half LIM domains protein 1

FHL3_MOUSE	2147	33769	72	8	8	Four and a half LIM domains protein 3
FHOD3_MOUSE	34	176630	2	1	1	FH1/FH2 domain-containing protein 3
FIBA_MOUSE	253	88117	15	5	5	Fibrinogen alpha chain
FIBB_MOUSE	608	55402	47	10	10	Fibrinogen beta chain
FIBG_MOUSE	927	50044	51	7	7	Fibrinogen gamma chain
FINC_MOUSE	1020	276017	49	17	17	Fibronectin
FIS1_MOUSE	367	17055	24	3	3	Mitochondrial fission 1 protein
FITM1_MOUSE	1200	32715	77	6	6	Fat storage-inducing transmembrane protein 1
FKB1A_MOUSE	658	11972	35	2	2	Peptidyl-prolyl cis-trans isomerase FKBP1A
FKBP3_MOUSE	60	25189	2	1	1	Peptidyl-prolyl cis-trans isomerase FKBP3
FLNA_MOUSE	2710	283897	163	10	4	Filamin-A
FLNB_MOUSE	4345	280103	182	9	2	Filamin-B
FLNC_MOUSE	52658	293560	2334	108	99	Filamin-C
FMN1_MOUSE	20	164791	1	1	1	Formin-1
FMO1_MOUSE	49	60446	3	1	1	Dimethylaniline monooxygenase [N-oxide-forming] 1
FMOD_MOUSE	9629	43427	396	9	9	Fibromodulin
FN3K_MOUSE	359	35238	16	2	2	Fructosamine-3-kinase
FRIH_MOUSE	641	21224	50	7	7	Ferritin heavy chain
FRIL1_MOUSE	896	20847	33	4	4	Ferritin light chain 1
FSD2_MOUSE	40	79985	2	1	1	Fibronectin type III and SPRY domain-containing protein 2
FUMH_MOUSE	6203	54550	315	18	18	Fumarate hydratase, mitochondrial
FUND2_MOUSE	119	16611	9	1	1	FUN14 domain-containing protein 2
FXR1_MOUSE	1373	76517	58	5	5	Fragile X mental retardation syndrome-related protein 1
G3BP1_MOUSE	32	51854	1	1	1	Ras GTPase-activating protein-binding protein 1
G3P_MOUSE	72851	36072	2721	26	26	Glyceraldehyde-3-phosphate dehydrogenase
G45IP_MOUSE	57	25861	6	1	1	Growth arrest and DNA damage-inducible proteins-interacting protein 1
G6PI_MOUSE	7825	62955	433	28	28	Glucose-6-phosphate isomerase
GARS_MOUSE	67	82624	4	1	1	Glycine--tRNA ligase
GBB1_MOUSE	473	38151	31	4	1	Guanine nucleotide-binding protein G(I)/G(S)/G(T) subunit beta-1
GBB2_MOUSE	476	38048	32	4	1	Guanine nucleotide-binding protein G(I)/G(S)/G(T) subunit beta-2
GCAA_MOUSE	360	36936	24	4	4	Ig gamma-2A chain C region, A allele
GCDH_MOUSE	24	49088	1	1	1	Glutaryl-CoA dehydrogenase, mitochondrial
GDIA_MOUSE	759	51059	37	4	1	Rab GDP dissociation inhibitor alpha
GDIB_MOUSE	2464	51018	105	9	6	Rab GDP dissociation inhibitor beta
GDIR1_MOUSE	278	23450	16	4	4	Rho GDP-dissociation inhibitor 1
GELS_MOUSE	269	86287	27	7	7	Gelsolin
GEPH_MOUSE	135	83971	5	2	2	Gephyrin
GFPT1_MOUSE	17	79287	1	1	1	Glutamine--fructose-6-phosphate aminotransferase [isomerizing] 1
GLGB_MOUSE	57	80769	4	3	3	1,4-alpha-glucan-branching enzyme
GLNA_MOUSE	19	42834	1	1	1	Glutamine synthetase
GLO2_MOUSE	48	34519	2	1	1	Hydroxyacylglutathione hydrolase, mitochondrial
GLOD4_MOUSE	170	33581	11	1	1	Glyoxalase domain-containing protein 4
GLRX1_MOUSE	32	12148	1	1	1	Glutaredoxin-1
GLYG_MOUSE	2863	37606	146	10	10	Glycogenin-1
GMPR1_MOUSE	494	37971	25	3	3	GMP reductase 1
GNAL_MOUSE	204	44794	7	1	1	Guanine nucleotide-binding protein G(olf) subunit alpha
GPC1_MOUSE	70	62404	6	4	4	Glypican-1
GPDA_MOUSE	8091	38176	417	23	23	Glycerol-3-phosphate dehydrogenase [NAD(+)], cytoplasmic
GPDM_MOUSE	5792	81416	298	26	26	Glycerol-3-phosphate dehydrogenase, mitochondrial
GPSM2_MOUSE	18	76285	1	1	1	G-protein-signaling modulator 2
GPX3_MOUSE	77	25720	8	1	1	Glutathione peroxidase 3
GPX4_MOUSE	466	22925	20	2	2	Phospholipid hydroperoxide glutathione peroxidase
GRCR2_MOUSE	18	29096	1	1	1	Glutaredoxin domain-containing cysteine-rich protein 2
GRHPR_MOUSE	115	35706	9	3	3	Glyoxylate reductase/hydroxypyruvate reductase
GRP75_MOUSE	3471	73701	167	19	19	Stress-70 protein, mitochondrial
GSTK1_MOUSE	160	25801	4	2	2	Glutathione S-transferase kappa 1
GSTM1_MOUSE	3551	26067	132	13	10	Glutathione S-transferase Mu 1
GSTM2_MOUSE	2497	25871	82	6	3	Glutathione S-transferase Mu 2
GSTM5_MOUSE	1102	27016	49	4	3	Glutathione S-transferase Mu 5
GSTP1_MOUSE	3161	23765	155	8	8	Glutathione S-transferase P 1
GTR4_MOUSE	30	54948	1	1	1	Solute carrier family 2, facilitated glucose transporter member 4
GYS1_MOUSE	4528	84672	245	18	18	Glycogen [starch] synthase, muscle
H10_MOUSE	132	20848	10	3	3	Histone H1.0
H12_MOUSE	254	21254	17	4	1	Histone H1.2
H14_MOUSE	263	21964	17	4	1	Histone H1.4
H2A2A_MOUSE	4335	14087	206	7	4	Histone H2A type 2-A
H2AV_MOUSE	1496	13501	87	4	2	Histone H2A.V
H2AX_MOUSE	1711	15133	111	6	3	Histone H2AX
H2AY_MOUSE	536	39882	28	3	3	Core histone macro-H2A.1
H2B1B_MOUSE	5560	13944	211	8	8	Histone H2B type 1-B
H31_MOUSE	689	15509	50	4	1	Histone H3.1
H32_MOUSE	1781	15436	80	4	1	Histone H3.2
H3C_MOUSE	4426	15363	146	4	1	Histone H3.3C
H4_MOUSE	7345	11360	317	7	7	Histone H4
HACD1_MOUSE	20	32477	1	1	1	Very-long-chain (3R)-3-hydroxyacyl-CoA dehydratase 1
HBA_MOUSE	8638	15133	559	9	9	Hemoglobin subunit alpha
HBB1_MOUSE	27119	15944	955	16	8	Hemoglobin subunit beta-1
HBB2_MOUSE	14116	15982	678	15	7	Hemoglobin subunit beta-2
HCD2_MOUSE	2638	27516	103	7	7	3-hydroxyacyl-CoA dehydrogenase type-2
HCDH_MOUSE	3084	34613	128	9	9	Hydroxyacyl-coenzyme A dehydrogenase, mitochondrial
HDHD2_MOUSE	116	28883	11	2	2	Haloacid dehalogenase-like hydrolase domain-containing protein 2
HEM2_MOUSE	28	36456	2	1	1	Delta-aminolevulinic acid dehydratase
HEMH_MOUSE	170	47670	9	2	2	Ferrochelatase, mitochondrial

HEMO_MOUSE	938	52026	83	12	12	Hemopexin
HERC2_MOUSE	28	533966	1	1	1	E3 ubiquitin-protein ligase HERC2
HGFA_MOUSE	19	72860	1	1	1	Hepatocyte growth factor activator
HHATL_MOUSE	5389	57021	252	12	12	Protein-cysteine N-palmitoyltransferase HHAT-like protein
HIBCH_MOUSE	71	43295	6	3	3	3-hydroxyisobutyryl-CoA hydrolase, mitochondrial
HINT1_MOUSE	133	13882	12	3	3	Histidine triad nucleotide-binding protein 1
HINT2_MOUSE	29	17366	2	1	1	Histidine triad nucleotide-binding protein 2, mitochondrial
HMGB1_MOUSE	30	25049	2	1	1	High mobility group protein B1
HMMR_MOUSE	20	92242	1	1	1	Hyaluronan mediated motility receptor
HNRH1_MOUSE	119	49454	4	2	2	Heterogeneous nuclear ribonucleoprotein H
HNRPD_MOUSE	53	38501	7	2	2	Heterogeneous nuclear ribonucleoprotein D0
HNRPK_MOUSE	2418	51230	101	6	6	Heterogeneous nuclear ribonucleoprotein K
HNRPQ_MOUSE	77	69818	5	2	2	Heterogeneous nuclear ribonucleoprotein Q
HNRPU_MOUSE	125	88661	8	4	4	Heterogeneous nuclear ribonucleoprotein U
HRG_MOUSE	32	60095	2	1	1	Histidine-rich glycoprotein
HS12B_MOUSE	115	76812	5	1	1	Heat shock 70 kDa protein 12B
HS71A_MOUSE	3129	70321	140	7	2	Heat shock 70 kDa protein 1A
HS90A_MOUSE	3626	85134	157	10	2	Heat shock protein HSP 90-alpha
HS90B_MOUSE	8991	83571	480	27	17	Heat shock protein HSP 90-beta
HSDL2_MOUSE	37	54516	2	2	2	Hydroxysteroid dehydrogenase-like protein 2
HSP74_MOUSE	21	94872	1	1	1	Heat shock 70 kDa protein 4
HSP7C_MOUSE	27870	71055	1251	37	31	Heat shock cognate 71 kDa protein
HSPB1_MOUSE	2116	23057	139	10	10	Heat shock protein beta-1
HSPB2_MOUSE	73	20419	4	2	2	Heat shock protein beta-2
HSPB6_MOUSE	2094	17567	122	8	8	Heat shock protein beta-6
HSPB7_MOUSE	791	18680	34	4	4	Heat shock protein beta-7
HSPB8_MOUSE	87	21691	6	2	2	Heat shock protein beta-8
HVM12_MOUSE	24	13089	1	1	1	Ig heavy chain V region MOPC 104E
HXK2_MOUSE	3317	103951	206	25	25	Hexokinase-2
HYEP_MOUSE	30	52714	1	1	1	Epoxide hydrolase 1
HYES_MOUSE	44	63045	2	1	1	Bifunctional epoxide hydrolase 2
I20RA_MOUSE	19	62680	1	1	1	Interleukin-20 receptor subunit alpha
I5P2_MOUSE	22	114002	3	1	1	Type II inositol 1,4,5-trisphosphate 5-phosphatase
IDH3A_MOUSE	9831	40069	448	16	16	Isocitrate dehydrogenase [NAD] subunit alpha, mitochondrial
IDHC_MOUSE	230	47044	27	4	3	Isocitrate dehydrogenase [NADP] cytoplasmic
IDHG1_MOUSE	4048	43157	266	12	12	Isocitrate dehydrogenase [NAD] subunit gamma 1, mitochondrial
IDHP_MOUSE	7085	51330	429	23	22	Isocitrate dehydrogenase [NADP], mitochondrial
IF2A_MOUSE	175	36371	10	2	2	Eukaryotic translation initiation factor 2 subunit 1
IF2G_MOUSE	161	51603	11	2	2	Eukaryotic translation initiation factor 2 subunit 3, X-linked
IF2P_MOUSE	51	137988	3	1	1	Eukaryotic translation initiation factor 5B
IF4A1_MOUSE	1244	46353	72	9	3	Eukaryotic initiation factor 4A-I
IF4A2_MOUSE	1346	46601	77	9	3	Eukaryotic initiation factor 4A-II
IF4B_MOUSE	42	68970	1	1	1	Eukaryotic translation initiation factor 4B
IF4G1_MOUSE	205	176823	16	3	3	Eukaryotic translation initiation factor 4 gamma 1
IF4G2_MOUSE	29	102554	1	1	1	Eukaryotic translation initiation factor 4 gamma 2
IF5A1_MOUSE	2010	17049	121	7	7	Eukaryotic translation initiation factor 5A-1
IGG2B_MOUSE	81	44972	6	3	3	Ig gamma-2B chain C region
IGH1M_MOUSE	325	44043	17	5	5	Ig gamma-1 chain C region, membrane-bound form
IGHM_MOUSE	32	50625	2	1	1	Immunoglobulin heavy constant mu
IGKC_MOUSE	213	12098	20	5	5	Immunoglobulin kappa constant
ILK_MOUSE	43	51853	3	1	1	Integrin-linked protein kinase
IMA3_MOUSE	351	58400	16	1	1	Importin subunit alpha-3
IMA4_MOUSE	158	58193	14	3	3	Importin subunit alpha-4
IMB1_MOUSE	476	98377	23	4	4	Importin subunit beta-1
INMT_MOUSE	111	30068	4	1	1	Indolethylamine N-methyltransferase
INO1_MOUSE	33	61520	1	1	1	Inositol-3-phosphate synthase 1
INP4B_MOUSE	18	105378	1	1	1	Type II inositol 3,4-bisphosphate 4-phosphatase
INP5K_MOUSE	32	54295	1	1	1	Inositol polyphosphate 5-phosphatase K
IPO5_MOUSE	357	124993	22	4	4	Importin-5
IPO7_MOUSE	50	120721	2	2	2	Importin-7
IQGA2_MOUSE	20	181100	1	1	1	Ras GTPase-activating-like protein IQGAP2
IQUB_MOUSE	22	92756	1	1	1	IQ and ubiquitin-like domain-containing protein
ISC2A_MOUSE	260	22745	18	4	4	Isochorismatase domain-containing protein 2A
ITA7_MOUSE	66	130502	7	3	3	Integrin alpha-7
ITB1_MOUSE	97	91424	11	4	4	Integrin beta-1
ITBP2_MOUSE	51	39541	2	1	1	Integrin beta-1-binding protein 2
ITIH4_MOUSE	37	104765	2	1	1	Inter alpha-trypsin inhibitor, heavy chain 4
IVD_MOUSE	750	46695	46	9	9	Isovaleryl-CoA dehydrogenase, mitochondrial
JAM3_MOUSE	84	35386	9	1	1	Junctional adhesion molecule C
JPH1_MOUSE	330	72374	38	13	13	Junctophilin-1
JPH2_MOUSE	852	74818	66	11	11	Junctophilin-2
JSPR1_MOUSE	2936	36242	148	10	10	Junctional sarcoplasmic reticulum protein 1
K1C15_MOUSE	160	49278	12	4	1	Keratin, type I cytoskeletal 15
K1C17_MOUSE	99	48417	8	3	1	Keratin, type I cytoskeletal 17
K1H1_MOUSE	8429	48911	301	20	2	Keratin, type I cuticular Ha1
K22O_MOUSE	187	63319	16	4	1	Keratin, type II cytoskeletal 2 oral
KAD1_MOUSE	20801	21640	696	15	15	Adenylate kinase isoenzyme 1
KAD2_MOUSE	71	26737	4	2	2	Adenylate kinase 2, mitochondrial
KANK4_MOUSE	62	111077	4	1	1	KN motif and ankyrin repeat domain-containing protein 4
KAP0_MOUSE	34	43443	3	2	2	cAMP-dependent protein kinase type I-alpha regulatory subunit
KAP2_MOUSE	1181	45703	68	8	8	cAMP-dependent protein kinase type II-alpha regulatory subunit
KAPCA_MOUSE	182	40659	6	2	2	cAMP-dependent protein kinase catalytic subunit alpha
KAT2B_MOUSE	59	92680	9	1	1	Histone acetyltransferase KAT2B

KCC2A_MOUSE	4594	54651	213	11	8	Calcium/calmodulin-dependent protein kinase type II subunit alpha
KCC2D_MOUSE	788	56961	46	7	3	Calcium/calmodulin-dependent protein kinase type II subunit delta
KCC2G_MOUSE	1398	60254	94	11	8	Calcium/calmodulin-dependent protein kinase type II subunit gamma
KCNK3_MOUSE	21	45723	1	1	1	Potassium channel subfamily K member 3
KCRB_MOUSE	334	42971	37	3	2	Creatine kinase B-type
KCRM_MOUSE	139811	43246	5228	40	39	Creatine kinase M-type
KCRS_MOUSE	37940	47899	1517	22	22	Creatine kinase S-type, mitochondrial
KCTD1_MOUSE	28	29671	1	1	1	BTB/POZ domain-containing protein KCTD1
KERA_MOUSE	1107	40777	64	8	8	Keratocan
KIF15_MOUSE	114	161104	17	1	1	Kinesin-like protein KIF15
KISHA_MOUSE	20	8353	1	1	1	Protein kish-A
KISHB_MOUSE	18	8474	1	1	1	Protein kish-B
KLH40_MOUSE	5869	70400	236	21	21	Kelch-like protein 40
KLH41_MOUSE	17920	68945	790	31	31	Kelch-like protein 41
KNG1_MOUSE	19	74140	1	1	1	Kininogen-1
KPB1_MOUSE	4198	139877	200	24	24	Phosphorylase b kinase regulatory subunit alpha, skeletal muscle isoform
KPBB_MOUSE	2864	124951	128	21	21	Phosphorylase b kinase regulatory subunit beta
KPRA_MOUSE	48	39692	4	2	2	Phosphoribosyl pyrophosphate synthase-associated protein 1
KPYM_MOUSE	52332	58378	1957	34	34	Pyruvate kinase PKM
KR195_MOUSE	123	6798	4	1	1	Keratin-associated protein 19-5
KRA65_MOUSE	76	8509	4	1	1	Keratin-associated protein 6-5
KRT34_MOUSE	7669	46185	292	23	4	Keratin, type I cuticular Ha4
KRT85_MOUSE	3466	57377	222	20	5	Keratin, type II cuticular Hb5
KRT86_MOUSE	7924	55098	381	32	10	Keratin, type II cuticular Hb6
KRT87_MOUSE	6250	56190	326	29	7	Keratin, type II cuticular 87
KT33A_MOUSE	8613	47704	307	20	4	Keratin, type I cuticular Ha3-I
KT33B_MOUSE	6596	47488	254	21	5	Keratin, type I cuticular Ha3-II
L2HDH_MOUSE	40	51551	6	1	1	L-2-hydroxyglutarate dehydrogenase, mitochondrial
LAMA2_MOUSE	14216	352831	771	66	66	Laminin subunit alpha-2
LAMA4_MOUSE	292	204372	20	3	3	Laminin subunit alpha-4
LAMA5_MOUSE	589	416165	24	5	5	Laminin subunit alpha-5
LAMB1_MOUSE	3746	204203	187	21	21	Laminin subunit beta-1
LAMB2_MOUSE	4923	203579	295	41	41	Laminin subunit beta-2
LAMC1_MOUSE	8919	182830	434	33	33	Laminin subunit gamma-1
LAMP1_MOUSE	166	44293	8	1	1	Lysosome-associated membrane glycoprotein 1
LAP2A_MOUSE	50	75807	2	1	1	Lamina-associated polypeptide 2, isoforms alpha/zeta
LCAP_MOUSE	38	117799	4	1	1	Leucyl-cystinyl aminopeptidase
LDB3_MOUSE	21498	77581	997	23	23	LIM domain-binding protein 3
LDHA_MOUSE	26752	36817	1084	24	23	L-lactate dehydrogenase A chain
LDHB_MOUSE	6361	36834	258	15	14	L-lactate dehydrogenase B chain
LEG1_MOUSE	1078	15198	78	6	6	Galectin-1
LETM1_MOUSE	221	83621	21	6	6	Mitochondrial proton/calcium exchanger protein
LGUL_MOUSE	2103	20967	98	6	6	Lactoylglutathione lyase
LLPH_MOUSE	26	15495	1	1	1	Protein LLP homolog
LMAN1_MOUSE	18	58038	2	2	2	Protein ERGIC-53
LMCD1_MOUSE	38	42167	3	3	3	LIM and cysteine-rich domains protein 1
LMNA_MOUSE	10322	74478	508	37	35	Prelamin-A/C
LMNB1_MOUSE	1179	66973	89	11	8	Lamin-B1
LMNB2_MOUSE	416	67391	42	9	6	Lamin-B2
LMOD3_MOUSE	91	65762	5	1	1	Leiomodin-3
LNEBL_MOUSE	22	31492	1	1	1	LIM zinc-binding domain-containing Nebulette
LNP_MOUSE	100	47755	6	1	1	Endoplasmic reticulum junction formation protein lunapark
LONM_MOUSE	269	106347	24	9	9	Lon protease homolog, mitochondrial
LPPRC_MOUSE	1895	157770	115	14	14	Leucine-rich PPR motif-containing protein, mitochondrial
LRBA_MOUSE	32	319828	4	1	1	Lipopolysaccharide-responsive and beige-like anchor protein
LRC20_MOUSE	121	20910	12	2	2	Leucine-rich repeat-containing protein 20
LRC39_MOUSE	23	39321	2	1	1	Leucine-rich repeat-containing protein 39
LRC8C_MOUSE	25	93338	1	1	1	Volume-regulated anion channel subunit LRRC8C
LRRK1_MOUSE	18	228405	1	1	1	Leucine-rich repeat serine/threonine-protein kinase 1
LUM_MOUSE	6121	38640	335	12	12	Lumican
LX12E_MOUSE	32	76092	1	1	1	Arachidonate 12-lipoxygenase, epidermal-type
LY6C1_MOUSE	32	14867	2	1	1	Lymphocyte antigen 6C1
LYAG_MOUSE	52	106921	2	1	1	Lysosomal alpha-glucosidase
LYPA1_MOUSE	24	25014	2	2	2	Acyl-protein thioesterase 1
LZTL1_MOUSE	19	34809	1	1	1	Leucine zipper transcription factor-like protein 1
M2OM_MOUSE	7520	34304	347	15	15	Mitochondrial 2-oxoglutarate/malate carrier protein
M4K4_MOUSE	22	141257	1	1	1	Mitogen-activated protein kinase kinase kinase kinase 4
MAAI_MOUSE	36	24431	1	1	1	Maleylacetoacetate isomerase
MACD1_MOUSE	6182	35843	211	10	10	O-acetyl-ADP-ribose deacetylase MACROD1
MACF1_MOUSE	45	836893	7	2	2	Microtubule-actin cross-linking factor 1
MAON_MOUSE	1156	67683	42	3	3	NADP-dependent malic enzyme, mitochondrial
MAOX_MOUSE	487	64540	32	7	7	NADP-dependent malic enzyme
MAP1B_MOUSE	25	271343	1	1	1	Microtubule-associated protein 1B
MAP1S_MOUSE	33	103902	1	1	1	Microtubule-associated protein 1S
MAVS_MOUSE	56	53822	6	3	3	Mitochondrial antiviral-signaling protein
MBB1A_MOUSE	19	152854	1	1	1	Myb-binding protein 1A
MBP_MOUSE	1518	27151	112	6	6	Myelin basic protein
MCA3_MOUSE	30	19903	1	1	1	Eukaryotic translation elongation factor 1 epsilon-1
MCAF2_MOUSE	33	51513	2	1	1	Activating transcription factor 7-interacting protein 2
MCAT_MOUSE	236	33347	21	4	4	Mitochondrial carnitine/acylcarnitine carrier protein
MCCA_MOUSE	74	79692	5	2	2	Methylcrotonoyl-CoA carboxylase subunit alpha, mitochondrial
MCCB_MOUSE	174	61910	10	2	2	Methylcrotonoyl-CoA carboxylase beta chain, mitochondrial
MCM5_MOUSE	41	83145	7	1	1	DNA replication licensing factor MCM5

MCPT4_MOUSE	287	27528	24	5	5	Mast cell protease 4
MCTS1_MOUSE	29	20770	1	1	1	Malignant T-cell-amplified sequence 1
MCU_MOUSE	238	39999	16	4	4	Calcium uniporter protein, mitochondrial
MDHC_MOUSE	10504	36659	422	15	15	Malate dehydrogenase, cytoplasmic
MDHM_MOUSE	34989	36045	1280	19	19	Malate dehydrogenase, mitochondrial
MECR_MOUSE	27	40545	2	2	2	Enoyl-[acyl-carrier-protein] reductase, mitochondrial
MED25_MOUSE	28	78577	4	1	1	Mediator of RNA polymerase II transcription subunit 25
MET15_MOUSE	17	45482	1	1	1	Probable methyltransferase-like protein 15
MFAP4_MOUSE	94	29261	4	2	2	Microfibril-associated glycoprotein 4
MFGM_MOUSE	23	52348	1	1	1	Lactadherin
MFN2_MOUSE	19	86874	1	1	1	Mitofusin-2
MGDP1_MOUSE	38	18628	2	2	2	Magnesium-dependent phosphatase 1
MGLL_MOUSE	53	33708	2	1	1	Monoglyceride lipase
MGST3_MOUSE	1857	17118	43	4	4	Microsomal glutathione S-transferase 3
MIC10_MOUSE	18	8675	1	1	1	MIC complex subunit Mic10
MIC13_MOUSE	1465	13422	43	1	1	MIC complex subunit MIC13
MIC19_MOUSE	2834	26546	118	11	11	MIC complex subunit Mic19
MIC26_MOUSE	553	22704	41	5	5	MIC complex subunit Mic26
MIC27_MOUSE	118	29356	9	3	3	MIC complex subunit Mic27
MIC60_MOUSE	7864	84247	414	35	35	MIC complex subunit Mic60
MICU3_MOUSE	31	60058	2	1	1	Calcium uptake protein 3, mitochondrial
MID49_MOUSE	26	50049	1	1	1	Mitochondrial dynamics protein MID49
MIF_MOUSE	657	12667	44	2	2	Macrophage migration inhibitory factor
MIME_MOUSE	7233	34333	213	10	10	Mimecan
MK01_MOUSE	106	41648	10	2	2	Mitogen-activated protein kinase 1
ML12B_MOUSE	22	19824	1	1	1	Myosin regulatory light chain 12B
MLIP_MOUSE	64	29505	2	1	1	Muscular LMNA-interacting protein
MLP3A_MOUSE	52	14320	4	1	1	Microtubule-associated proteins 1A/1B light chain 3A
MLRS_MOUSE	72232	19057	3037	22	22	Myosin regulatory light chain 2, skeletal muscle isoform
MLRV_MOUSE	5204	18852	300	13	13	Myosin regulatory light chain 2, ventricular/cardiac muscle isoform
MMP17_MOUSE	29	64635	1	1	1	Matrix metalloproteinase-17
MMP20_MOUSE	31	54510	4	1	1	Matrix metalloproteinase-20
MMSA_MOUSE	1148	58335	78	12	12	Methylmalonate-semialdehyde dehydrogenase [acylating], mitochondrial
MOAP1_MOUSE	26	39664	1	1	1	Modulator of apoptosis 1
MOT1_MOUSE	24	53860	1	1	1	Monocarboxylate transporter 1
MOT4_MOUSE	80	50968	6	2	2	Monocarboxylate transporter 4
MP2K3_MOUSE	31	39613	1	1	1	Dual specificity mitogen-activated protein kinase kinase 3
MP2K4_MOUSE	45	44542	1	1	1	Dual specificity mitogen-activated protein kinase kinase 4
MPC1_MOUSE	2327	12617	91	3	3	Mitochondrial pyruvate carrier 1
MPC2_MOUSE	408	14334	28	4	4	Mitochondrial pyruvate carrier 2
MPCP_MOUSE	7131	40063	353	16	16	Phosphate carrier protein, mitochondrial
MPI_MOUSE	18	47229	1	1	1	Mannose-6-phosphate isomerase
MPPA_MOUSE	1215	58755	24	1	1	Mitochondrial-processing peptidase subunit alpha
MPPB_MOUSE	350	55378	22	2	1	Mitochondrial-processing peptidase subunit beta
MRAP2_MOUSE	18	23778	2	1	1	Melanocortin-2 receptor accessory protein 2
MRGB3_MOUSE	62	36341	7	1	1	Mas-related G-protein coupled receptor member B3
MSPD1_MOUSE	28	24401	3	2	2	Motile sperm domain-containing protein 1
MTAP2_MOUSE	23	199409	1	1	1	Microtubule-associated protein 2
MTCH2_MOUSE	1188	34104	90	7	7	Mitochondrial carrier homolog 2
MTFP1_MOUSE	403	18360	22	2	2	Mitochondrial fission process protein 1
MTMR9_MOUSE	26	63609	1	1	1	Myotubularin-related protein 9
MTX1_MOUSE	99	35886	8	1	1	Metaxin-1
MTX2_MOUSE	134	30081	8	3	3	Metaxin-2
MUG1_MOUSE	57	166618	4	1	1	Murinoglobulin-1
MVP_MOUSE	236	96150	17	8	8	Major vault protein
MY18A_MOUSE	510	233637	32	6	6	Unconventional myosin-XVIIIa
MYBPH_MOUSE	14113	53065	582	19	19	Myosin-binding protein H
MYBPP_MOUSE	99	106785	13	1	1	MYCBP-associated protein
MYG_MOUSE	23310	17116	937	15	15	Myoglobin
MYH1_MOUSE	697229	224116	23704	211	47	Myosin-1
MYH10_MOUSE	4791	229824	231	4	1	Myosin-10
MYH11_MOUSE	5088	227743	252	4	1	Myosin-11
MYH3_MOUSE	212948	224736	8638	86	12	Myosin-3
MYH4_MOUSE	1449536	223632	43466	241	72	Myosin-4
MYH7_MOUSE	257369	223539	9252	151	97	Myosin-7
MYH7B_MOUSE	32339	222501	1298	25	8	Myosin-7B
MYH8_MOUSE	578992	223653	19275	153	14	Myosin-8
MYH9_MOUSE	6058	227429	303	12	8	Myosin-9
MYL1_MOUSE	82533	20695	2754	22	18	Myosin light chain 1/3, skeletal muscle isoform
MYL3_MOUSE	31631	22521	1151	17	13	Myosin light chain 3
MYL4_MOUSE	4099	21260	127	3	1	Myosin light chain 4
MYL6B_MOUSE	589	22849	33	7	7	Myosin light chain 6B
MYL9_MOUSE	20	19898	1	1	1	Myosin regulatory light polypeptide 9
MYLK2_MOUSE	385	66633	48	11	11	Myosin light chain kinase 2, skeletal/cardiac muscle
MYO1C_MOUSE	218	122723	9	2	2	Unconventional myosin-Ic
MYOC_MOUSE	84	55622	6	3	3	Myocilin
MYOM1_MOUSE	110842	186546	4933	112	112	Myomesin-1
MYOM3_MOUSE	12940	162761	563	57	57	Myomesin-3
MYOTI_MOUSE	11916	55738	761	28	28	Myotilin
MYOZ1_MOUSE	25659	31438	960	16	16	Myozenin-1
MYOZ2_MOUSE	1355	29800	95	9	9	Myozenin-2
MYOZ3_MOUSE	4982	27022	181	10	10	Myozenin-3
MYP0_MOUSE	4989	27775	225	8	8	Myelin protein P0

MYPC2_MOUSE	145775	128128	5614	94	94	Myosin-binding protein C, fast-type
MYPN_MOUSE	1394	145222	102	16	15	Myopalladin
MYPT2_MOUSE	26	109326	4	1	1	Protein phosphatase 1 regulatory subunit 12B
NACAM_MOUSE	3244	221277	217	38	38	Nascent polypeptide-associated complex subunit alpha, muscle-specific form
NAGAB_MOUSE	25	47717	2	2	2	Alpha-N-acetylgalactosaminidase
NAR1_MOUSE	876	36331	25	2	2	GPI-linked NAD(P)(+)--arginine ADP-ribosyltransferase 1
NAR3_MOUSE	371	42330	23	6	6	Ecto-ADP-ribosyltransferase 3
NB5R1_MOUSE	386	34284	31	7	7	NADH-cytochrome b5 reductase 1
NB5R3_MOUSE	1085	34334	72	11	11	NADH-cytochrome b5 reductase 3
NCAM1_MOUSE	22	120152	2	1	1	Neural cell adhesion molecule 1
NCKP1_MOUSE	19	130012	2	1	1	Nck-associated protein 1
NCLN_MOUSE	189	63097	10	1	1	Nicalin
NDE1_MOUSE	72	38556	19	1	1	Nuclear distribution protein nudE homolog 1
NDF2_MOUSE	20	41867	1	1	1	Neurogenic differentiation factor 2
NDF6_MOUSE	18	39075	1	1	1	Neurogenic differentiation factor 6
NDKB_MOUSE	3210	17466	208	8	8	Nucleoside diphosphate kinase B
NDRG2_MOUSE	4020	41105	192	10	10	Protein NDRG2
NDUA1_MOUSE	205	8190	8	1	1	NADH dehydrogenase [ubiquinone] 1 alpha subcomplex subunit 1
NDUA2_MOUSE	436	11023	26	5	5	NADH dehydrogenase [ubiquinone] 1 alpha subcomplex subunit 2
NDUA3_MOUSE	25	9325	1	1	1	NADH dehydrogenase [ubiquinone] 1 alpha subcomplex subunit 3
NDUA4_MOUSE	5550	9321	249	8	8	Cytochrome c oxidase subunit NDUFA4
NDUA5_MOUSE	1978	13408	94	4	4	NADH dehydrogenase [ubiquinone] 1 alpha subcomplex subunit 5
NDUA6_MOUSE	3172	15273	143	3	3	NADH dehydrogenase [ubiquinone] 1 alpha subcomplex subunit 6
NDUA7_MOUSE	380	12625	24	2	2	NADH dehydrogenase [ubiquinone] 1 alpha subcomplex subunit 7
NDUA8_MOUSE	2894	20435	168	11	11	NADH dehydrogenase [ubiquinone] 1 alpha subcomplex subunit 8
NDUA9_MOUSE	8176	42612	305	18	18	NADH dehydrogenase [ubiquinone] 1 alpha subcomplex subunit 9, mitochondrial
NDUAA_MOUSE	9724	40863	412	18	18	NADH dehydrogenase [ubiquinone] 1 alpha subcomplex subunit 10, mitochondrial
NDUAB_MOUSE	223	15258	19	3	3	NADH dehydrogenase [ubiquinone] 1 alpha subcomplex subunit 11
NDUAC_MOUSE	1463	17133	89	5	5	NADH dehydrogenase [ubiquinone] 1 alpha subcomplex subunit 12
NDUAD_MOUSE	802	16849	36	4	4	NADH dehydrogenase [ubiquinone] 1 alpha subcomplex subunit 13
NDUB1_MOUSE	930	7007	63	3	3	NADH dehydrogenase [ubiquinone] 1 beta subcomplex subunit 1
NDUB2_MOUSE	28	12017	3	2	2	NADH dehydrogenase [ubiquinone] 1 beta subcomplex subunit 2, mitochondrial
NDUB3_MOUSE	54	11685	7	3	3	NADH dehydrogenase [ubiquinone] 1 beta subcomplex subunit 3
NDUB4_MOUSE	1276	15072	59	5	5	NADH dehydrogenase [ubiquinone] 1 beta subcomplex subunit 4
NDUB5_MOUSE	1318	21753	73	6	6	NADH dehydrogenase [ubiquinone] 1 beta subcomplex subunit 5, mitochondrial
NDUB6_MOUSE	346	15505	40	5	5	NADH dehydrogenase [ubiquinone] 1 beta subcomplex subunit 6
NDUB7_MOUSE	755	16548	68	5	5	NADH dehydrogenase [ubiquinone] 1 beta subcomplex subunit 7
NDUB8_MOUSE	1124	21919	53	3	3	NADH dehydrogenase [ubiquinone] 1 beta subcomplex subunit 8, mitochondrial
NDUB9_MOUSE	642	22255	87	5	5	NADH dehydrogenase [ubiquinone] 1 beta subcomplex subunit 9
NDUBA_MOUSE	3892	21296	220	11	11	NADH dehydrogenase [ubiquinone] 1 beta subcomplex subunit 10
NDUBB_MOUSE	317	17490	25	1	1	NADH dehydrogenase [ubiquinone] 1 beta subcomplex subunit 11, mitochondrial
NDUC2_MOUSE	1039	14211	70	4	4	NADH dehydrogenase [ubiquinone] 1 subunit C2
NDUF4_MOUSE	46	20070	1	1	1	NADH dehydrogenase [ubiquinone] 1 alpha subcomplex assembly factor 4
NDUS1_MOUSE	15866	80752	712	36	36	NADH-ubiquinone oxidoreductase 75 kDa subunit, mitochondrial
NDUS2_MOUSE	4497	52991	245	16	16	NADH dehydrogenase [ubiquinone] iron-sulfur protein 2, mitochondrial
NDUS3_MOUSE	10391	30302	315	15	15	NADH dehydrogenase [ubiquinone] iron-sulfur protein 3, mitochondrial
NDUS4_MOUSE	374	19829	30	6	6	NADH dehydrogenase [ubiquinone] iron-sulfur protein 4, mitochondrial
NDUS5_MOUSE	1006	12867	35	3	3	NADH dehydrogenase [ubiquinone] iron-sulfur protein 5
NDUS6_MOUSE	119	13183	11	3	3	NADH dehydrogenase [ubiquinone] iron-sulfur protein 6, mitochondrial
NDUS7_MOUSE	3331	24952	118	5	5	NADH dehydrogenase [ubiquinone] iron-sulfur protein 7, mitochondrial
NDUS8_MOUSE	2685	24479	120	7	7	NADH dehydrogenase [ubiquinone] iron-sulfur protein 8, mitochondrial
NDUV1_MOUSE	7657	51486	405	21	21	NADH dehydrogenase [ubiquinone] flavoprotein 1, mitochondrial
NDUV2_MOUSE	1720	27610	90	6	6	NADH dehydrogenase [ubiquinone] flavoprotein 2, mitochondrial
NEBL_MOUSE	37	52339	3	1	1	Nebulette
NEDD8_MOUSE	298	8967	14	1	1	NEDD8
NEMO_MOUSE	32	48455	4	1	1	NF-kappa-B essential modulator
NEXN_MOUSE	87	72349	5	2	2	Nexilin
NFH_MOUSE	1188	117209	73	9	7	Neurofilament heavy polypeptide
NFL_MOUSE	3040	61528	198	22	19	Neurofilament light polypeptide
NFM_MOUSE	1937	95973	121	20	17	Neurofilament medium polypeptide
NID1_MOUSE	8272	139302	393	29	28	Nidogen-1
NID2_MOUSE	389	156610	34	9	8	Nidogen-2
NIPS2_MOUSE	2405	33083	144	11	11	Protein NipSnap homolog 2
NLRP6_MOUSE	174	98766	17	1	1	NACHT, LRR and PYD domains-containing protein 6
NLRX1_MOUSE	63	108789	5	1	1	NLR family member X1
NLTP_MOUSE	21	59715	1	1	1	Non-specific lipid-transfer protein
NNTM_MOUSE	1593	114449	106	18	18	NAD(P) transhydrogenase, mitochondrial
NOL3_MOUSE	177	24781	9	2	2	Nucleolar protein 3
NOMO1_MOUSE	20	134362	1	1	1	Nodal modulator 1
NONO_MOUSE	35	54620	4	2	2	Non-POU domain-containing octamer-binding protein
NP1L4_MOUSE	322	42824	20	2	2	Nucleosome assembly protein 1-like 4
NPM_MOUSE	73	32711	3	1	1	Nucleophosmin
NPTN_MOUSE	64	44688	5	2	2	Neuroplastin
NSF_MOUSE	75	83131	3	1	1	Vesicle-fusing ATPase
NSF1C_MOUSE	28	40685	2	1	1	NSFL1 cofactor p47
NTF2_MOUSE	18	14640	1	1	1	Nuclear transport factor 2
NTPCR_MOUSE	19	20825	1	1	1	Cancer-related nucleoside-triphosphatase homolog
NTRK1_MOUSE	23	88880	2	1	1	High affinity nerve growth factor receptor
NU1M_MOUSE	64	36092	6	1	1	NADH-ubiquinone oxidoreductase chain 1
NU3M_MOUSE	18	13267	1	1	1	NADH-ubiquinone oxidoreductase chain 3
NU4M_MOUSE	670	52018	39	4	4	NADH-ubiquinone oxidoreductase chain 4
NU5M_MOUSE	765	68771	30	2	2	NADH-ubiquinone oxidoreductase chain 5
NUCB2_MOUSE	31	50387	1	1	1	Nucleobindin-2

NUCG_MOUSE	48	32285	2	2	2	Endonuclease G, mitochondrial
NUCL_MOUSE	319	76734	25	4	4	Nucleolin
NUDT6_MOUSE	34	35517	1	1	1	Nucleoside diphosphate-linked moiety X motif 6
OAT_MOUSE	266	48723	13	5	5	Ornithine aminotransferase, mitochondrial
OBSCN_MOUSE	33569	979281	1892	167	167	Obscurin
OCAD2_MOUSE	958	17257	28	2	2	OCIA domain-containing protein 2
ODB2_MOUSE	57	53555	4	1	1	Lipoamide acyltransferase component of branched-chain alpha-keto acid dehydrogenase complex, mitochondrial
ODBB_MOUSE	31	43651	1	1	1	2-oxoisovalerate dehydrogenase subunit beta, mitochondrial
ODO1_MOUSE	21503	117572	1015	49	49	2-oxoglutarate dehydrogenase, mitochondrial
ODO2_MOUSE	7312	49306	306	11	11	Dihydrolipoyllysine-residue succinyltransferase component of 2-oxoglutarate dehydrogenase complex, mitochondrial
ODP2_MOUSE	11838	68469	548	22	22	Dihydrolipoyllysine-residue acetyltransferase component of pyruvate dehydrogenase complex, mitochondrial
ODPA_MOUSE	14732	43888	565	23	23	Pyruvate dehydrogenase E1 component subunit alpha, somatic form, mitochondrial
ODPB_MOUSE	12166	39254	576	14	14	Pyruvate dehydrogenase E1 component subunit beta, mitochondrial
ODPX_MOUSE	1198	54250	69	7	7	Pyruvate dehydrogenase protein X component, mitochondrial
OLA1_MOUSE	1254	44929	40	2	2	Obg-like ATPase 1
OPA1_MOUSE	367	111783	23	8	8	Dynamin-like 120 kDa protein, mitochondrial
OPA3_MOUSE	167	20382	17	2	2	Optic atrophy 3 protein homolog
ORN_MOUSE	20	26950	1	1	1	Oligoribonuclease, mitochondrial
OST48_MOUSE	277	49225	25	2	2	Dolichyl-diphosphooligosaccharide--protein glycosyltransferase 48 kDa subunit
OTUB1_MOUSE	70	31478	3	1	1	Ubiquitin thioesterase OTUB1
OXSR1_MOUSE	160	58462	15	3	3	Serine/threonine-protein kinase R1
PA2G4_MOUSE	30	44013	1	1	1	Proliferation-associated protein 2G4
PABP1_MOUSE	369	70854	19	2	2	Polyadenylate-binding protein 1
PACN3_MOUSE	1702	48897	94	7	7	Protein kinase C and casein kinase II substrate protein 3
PADI2_MOUSE	778	76942	53	7	7	Protein-arginine deiminase type-2
PAIRB_MOUSE	61	44801	2	1	1	Plasminogen activator inhibitor 1 RNA-binding protein
PALLD_MOUSE	58	153576	4	2	1	Palladin
PAR3L_MOUSE	26	133268	1	1	1	Partitioning defective 3 homolog B
PARD3_MOUSE	18	149668	2	1	1	Partitioning defective 3 homolog
PARK7_MOUSE	3874	20236	188	12	12	Protein/nucleic acid deglycase DJ-1
PARP9_MOUSE	81	97566	24	2	2	Poly [ADP-ribose] polymerase 9
PARVB_MOUSE	149	41700	12	1	1	Beta-parvin
PBIP1_MOUSE	5943	81514	257	18	18	Pre-B-cell leukemia transcription factor-interacting protein 1
PCBP1_MOUSE	230	37987	13	5	4	Poly(rC)-binding protein 1
PCBP2_MOUSE	198	38597	11	3	2	Poly(rC)-binding protein 2
PCCA_MOUSE	66	80498	5	3	3	Propionyl-CoA carboxylase alpha chain, mitochondrial
PCCB_MOUSE	397	58999	19	3	3	Propionyl-CoA carboxylase beta chain, mitochondrial
PDIA1_MOUSE	5074	57422	237	20	20	Protein disulfide-isomerase
PDIA3_MOUSE	353	57099	24	6	6	Protein disulfide-isomerase A3
PDIA6_MOUSE	98	48469	9	3	3	Protein disulfide-isomerase A6
PDK2_MOUSE	976	46239	42	5	5	[Pyruvate dehydrogenase (acetyl-transferring)] kinase isozyme 2, mitochondrial
PDK4_MOUSE	451	46909	21	4	4	[Pyruvate dehydrogenase (acetyl-transferring)] kinase isozyme 4, mitochondrial
PDLI1_MOUSE	24	36208	1	1	1	PDZ and LIM domain protein 1
PDLI3_MOUSE	6888	34734	262	10	10	PDZ and LIM domain protein 3
PDLI5_MOUSE	9413	64514	381	9	9	PDZ and LIM domain protein 5
PDLI7_MOUSE	5054	51170	180	7	7	PDZ and LIM domain protein 7
PDP1_MOUSE	167	61712	15	5	5	[Pyruvate dehydrogenase [acetyl-transferring]]-phosphatase 1, mitochondrial
PDPR_MOUSE	357	100079	21	2	2	Pyruvate dehydrogenase phosphatase regulatory subunit, mitochondrial
PEBP1_MOUSE	1344	20988	65	6	6	Phosphatidylethanolamine-binding protein 1
PEDF_MOUSE	141	46262	7	2	2	Pigment epithelium-derived factor
PEPD_MOUSE	27	55962	1	1	1	Xaa-Pro dipeptidase
PERI_MOUSE	1371	54349	98	8	4	Peripherin
PFKAM_MOUSE	55700	86070	2121	41	41	ATP-dependent 6-phosphofructokinase, muscle type
PGAM1_MOUSE	2243	28928	93	5	1	Phosphoglycerate mutase 1
PGAM2_MOUSE	23967	28980	1044	19	15	Phosphoglycerate mutase 2
PGBM_MOUSE	5679	407847	300	40	40	Basement membrane-specific heparan sulfate proteoglycan core protein
PGES2_MOUSE	336	43581	14	2	2	Prostaglandin E synthase 2
PGFS_MOUSE	27	22055	2	2	2	Prostamide/prostaglandin F synthase
PGK1_MOUSE	19748	44921	811	28	28	Phosphoglycerate kinase 1
PGM1_MOUSE	24936	61665	1103	35	35	Phosphoglucomutase-1
PGM5_MOUSE	725	62751	73	3	3	Phosphoglucomutase-like protein 5
PGP_MOUSE	228	34975	9	1	1	Glycerol-3-phosphate phosphatase
PGS1_MOUSE	1333	42069	76	11	10	Biglycan
PGS2_MOUSE	11358	40126	542	15	14	Decorin
PHB_MOUSE	6707	29859	260	14	14	Prohibitin
PHB2_MOUSE	7217	33276	279	17	17	Prohibitin-2
PHF8_MOUSE	35	114508	2	1	1	Histone lysine demethylase PHF8
PHKG1_MOUSE	283	45387	23	5	5	Phosphorylase b kinase gamma catalytic chain, skeletal muscle/heart isoform
PIGT_MOUSE	36	66347	5	2	2	GPI transamidase component PIG-T
PIMT_MOUSE	387	24676	25	4	4	Protein-L-isoaspartate(D-aspartate) O-methyltransferase
PIPNA_MOUSE	379	32101	11	1	1	Phosphatidylinositol transfer protein alpha isoform
PK3CD_MOUSE	32	121118	5	1	1	Phosphatidylinositol 4,5-bisphosphate 3-kinase catalytic subunit delta isoform
PLAK_MOUSE	31	82490	1	1	1	Junction plakoglobin
PLAP_MOUSE	21	88591	1	1	1	Phospholipase A-2-activating protein
PLCC_MOUSE	48	43952	3	2	2	1-acyl-sn-glycerol-3-phosphate acyltransferase gamma
PLCD4_MOUSE	213	93547	10	1	1	1-phosphatidylinositol 4,5-bisphosphate phosphodiesterase delta-4
PLCG1_MOUSE	20	150999	2	1	1	1-phosphatidylinositol 4,5-bisphosphate phosphodiesterase gamma-1
PLD1_MOUSE	31	124689	1	1	1	Phospholipase D1
PLEC_MOUSE	73778	535800	3490	238	238	Plectin
PLIN4_MOUSE	1336	140297	84	13	13	Perilipin-4
PLM_MOUSE	384	10544	21	2	2	Phospholemman
PLMN_MOUSE	28	93486	3	1	1	Plasminogen
PLPHP_MOUSE	29	30201	1	1	1	Pyridoxal phosphate homeostasis protein

PLPL2_MOUSE	44	54478	14	1	1	Patatin-like phospholipase domain-containing protein 2
PLSI_MOUSE	26	70763	1	1	1	Plastin-1
PLXB3_MOUSE	20	211540	1	1	1	Plexin-B3
PMFBP_MOUSE	30	120467	1	1	1	Polyamine-modulated factor 1-binding protein 1
PNKD_MOUSE	38	43503	1	1	1	Probable hydrolase PNKD
PO3F2_MOUSE	30	47348	1	1	1	POU domain, class 3, transcription factor 2
PON2_MOUSE	213	39764	11	2	2	Serum paraoxonase/arylesterase 2
PON3_MOUSE	24	39554	2	2	2	Serum paraoxonase/lactonase 3
POSTN_MOUSE	1043	93769	40	5	5	Periostin
PP1A_MOUSE	1360	38257	65	9	1	Serine/threonine-protein phosphatase PP1-alpha catalytic subunit
PP1B_MOUSE	1893	37961	101	11	3	Serine/threonine-protein phosphatase PP1-beta catalytic subunit
PP1R7_MOUSE	41	41380	4	1	1	Protein phosphatase 1 regulatory subunit 7
PP2AA_MOUSE	53	36156	7	3	3	Serine/threonine-protein phosphatase 2A catalytic subunit alpha isoform
PP2BA_MOUSE	91	59291	10	2	2	Serine/threonine-protein phosphatase 2B catalytic subunit alpha isoform
PPAC_MOUSE	155	18636	11	3	3	Low molecular weight phosphotyrosine protein phosphatase
PPIA_MOUSE	1418	18131	74	6	6	Peptidyl-prolyl cis-trans isomerase A
PPIB_MOUSE	502	23699	29	5	5	Peptidyl-prolyl cis-trans isomerase B
PPR3A_MOUSE	662	122443	46	15	15	Protein phosphatase 1 regulatory subunit 3A
PRAF3_MOUSE	790	21543	49	4	4	PRA1 family protein 3
PRAX_MOUSE	1260	147823	83	28	28	Periaxin
PRD16_MOUSE	26	142765	2	1	1	PR domain zinc finger protein 16
PRDX1_MOUSE	2027	22390	185	11	11	Peroxiredoxin-1
PRDX2_MOUSE	3257	21936	148	9	9	Peroxiredoxin-2
PRDX3_MOUSE	3368	28337	194	9	9	Thioredoxin-dependent peroxide reductase, mitochondrial
PRDX5_MOUSE	3471	22226	102	6	6	Peroxiredoxin-5, mitochondrial
PRDX6_MOUSE	1757	24969	87	8	8	Peroxiredoxin-6
PRELP_MOUSE	7988	43607	347	14	14	Prolargin
PRLR_MOUSE	31	68996	2	1	1	Prolactin receptor
PROF1_MOUSE	551	15119	42	4	4	Profilin-1
PROF2_MOUSE	60	15364	5	2	2	Profilin-2
PROP_MOUSE	25	52802	1	1	1	Properdin
PROX1_MOUSE	29	83701	3	1	1	Prospero homeobox protein 1
PRPS1_MOUSE	6967	35325	275	14	14	Ribose-phosphate pyrophosphokinase 1
PRR33_MOUSE	396	28673	23	2	2	Proline-rich protein 33
PRS10_MOUSE	68	44430	3	1	1	26S proteasome regulatory subunit 10B
PRS4_MOUSE	75	49325	5	2	2	26S proteasome regulatory subunit 4
PRS6A_MOUSE	39	49803	2	2	2	26S proteasome regulatory subunit 6A
PRS6B_MOUSE	111	47493	7	4	4	26S proteasome regulatory subunit 6B
PRS7_MOUSE	93	49016	7	3	3	26S proteasome regulatory subunit 7
PRS8_MOUSE	316	45768	24	4	4	26S proteasome regulatory subunit 8
PRVA_MOUSE	6436	11923	246	9	9	Parvalbumin alpha
PSA_MOUSE	28	103944	1	1	1	Puromycin-sensitive aminopeptidase
PSA1_MOUSE	413	29813	48	5	5	Proteasome subunit alpha type-1
PSA2_MOUSE	725	26024	32	4	4	Proteasome subunit alpha type-2
PSA3_MOUSE	470	28615	18	4	4	Proteasome subunit alpha type-3
PSA4_MOUSE	176	29737	18	4	4	Proteasome subunit alpha type-4
PSA5_MOUSE	169	26565	8	2	2	Proteasome subunit alpha type-5
PSA6_MOUSE	206	27811	16	3	3	Proteasome subunit alpha type-6
PSA7_MOUSE	125	28009	9	4	4	Proteasome subunit alpha type-7
PSB1_MOUSE	526	26583	17	3	3	Proteasome subunit beta type-1
PSB2_MOUSE	420	23063	18	3	3	Proteasome subunit beta type-2
PSB3_MOUSE	674	23235	24	3	3	Proteasome subunit beta type-3
PSB5_MOUSE	158	28685	9	5	5	Proteasome subunit beta type-5
PSB6_MOUSE	157	25591	10	2	2	Proteasome subunit beta type-6
PSB7_MOUSE	40	30214	3	2	2	Proteasome subunit beta type-7
PSD1_MOUSE	195	110360	23	1	1	PH and SEC7 domain-containing protein 1
PSD11_MOUSE	241	47692	24	5	5	26S proteasome non-ATPase regulatory subunit 11
PSD12_MOUSE	218	53261	9	3	3	26S proteasome non-ATPase regulatory subunit 12
PSD13_MOUSE	637	43124	30	2	2	26S proteasome non-ATPase regulatory subunit 13
PSDE_MOUSE	143	34726	13	3	3	26S proteasome non-ATPase regulatory subunit 14
PSMD1_MOUSE	26	106689	2	1	1	26S proteasome non-ATPase regulatory subunit 1
PSMD2_MOUSE	480	100937	33	6	6	26S proteasome non-ATPase regulatory subunit 2
PSMD7_MOUSE	965	36574	31	4	4	26S proteasome non-ATPase regulatory subunit 7
PSMD8_MOUSE	109	40190	9	2	2	26S proteasome non-ATPase regulatory subunit 8
PTER_MOUSE	27	39535	1	1	1	Phosphotriesterase-related protein
PTH2_MOUSE	261	19742	19	2	2	Peptidyl-tRNA hydrolase 2, mitochondrial
PTPRB_MOUSE	36	225838	4	1	1	Receptor-type tyrosine-protein phosphatase beta
PTPRF_MOUSE	35	212439	1	1	1	Receptor-type tyrosine-protein phosphatase F
PUR2_MOUSE	70	108633	2	2	2	Trifunctional purine biosynthetic protein adenosine-3
PUR6_MOUSE	40	47717	2	2	2	Multifunctional protein ADE2
PUR8_MOUSE	583	55572	32	6	6	Adenylosuccinate lyase
PUR9_MOUSE	111	64690	8	1	1	Bifunctional purine biosynthesis protein PURH
PURA_MOUSE	617	34976	33	5	5	Transcriptional activator protein Pur-alpha
PURA1_MOUSE	8952	50508	410	22	21	Adenylosuccinate synthetase isozyme 1
PURA2_MOUSE	209	50389	13	2	1	Adenylosuccinate synthetase isozyme 2
PXMP2_MOUSE	48	22251	3	1	1	Peroxisomal membrane protein 2
PYC_MOUSE	711	130344	30	5	5	Pyruvate carboxylase, mitochondrial
PYGB_MOUSE	38821	97353	1327	20	3	Glycogen phosphorylase, brain form
PYGM_MOUSE	144694	97681	5053	76	59	Glycogen phosphorylase, muscle form
PYRG1_MOUSE	28	67324	1	1	1	CTP synthase 1
PZP_MOUSE	969	167116	61	11	11	Pregnancy zone protein
QCR1_MOUSE	19166	53446	905	21	20	Cytochrome b-c1 complex subunit 1, mitochondrial
QCR10_MOUSE	907	6534	47	1	1	Cytochrome b-c1 complex subunit 10

QCR2_MOUSE	24893	48262	969	22	22	Cytochrome b-c1 complex subunit 2, mitochondrial
QCR6_MOUSE	614	10713	17	3	3	Cytochrome b-c1 complex subunit 6, mitochondrial
QCR7_MOUSE	2034	13519	167	7	7	Cytochrome b-c1 complex subunit 7
QCR8_MOUSE	1456	9762	88	5	5	Cytochrome b-c1 complex subunit 8
QCR9_MOUSE	1431	7441	75	3	3	Cytochrome b-c1 complex subunit 9
RA51B_MOUSE	21	38755	3	2	2	DNA repair protein RAD51 homolog 2
RAB10_MOUSE	2721	22755	109	5	3	Ras-related protein Rab-10
RAB18_MOUSE	68	23306	5	4	4	Ras-related protein Rab-18
RAB1A_MOUSE	1446	22891	72	5	3	Ras-related protein Rab-1A
RAB21_MOUSE	36	24490	4	1	1	Ras-related protein Rab-21
RAB2A_MOUSE	159	23704	16	5	5	Ras-related protein Rab-2A
RAB35_MOUSE	1420	23296	68	3	2	Ras-related protein Rab-35
RAB5A_MOUSE	70	23812	5	3	3	Ras-related protein Rab-5A
RAB7A_MOUSE	183	23760	19	5	5	Ras-related protein Rab-7a
RAC1_MOUSE	80	21835	11	3	2	Ras-related C3 botulinum toxin substrate 1
RACK1_MOUSE	1184	35511	50	9	9	Receptor of activated protein C kinase 1
RAD1_MOUSE	22	32274	1	1	1	Cell cycle checkpoint protein RAD1
RADI_MOUSE	123	68614	11	4	4	Radixin
RAN_MOUSE	300	24579	21	4	4	GTP-binding nuclear protein Ran
RAP1A_MOUSE	1696	21316	60	5	5	Ras-related protein Rap-1A
RARA_MOUSE	27	51729	1	1	1	Retinoic acid receptor alpha
RASH_MOUSE	38	21627	3	1	1	GTPase HRas
RB11B_MOUSE	160	24589	13	7	7	Ras-related protein Rab-11B
RBBP5_MOUSE	32	59745	1	1	1	Retinoblastoma-binding protein 5
RBM3_MOUSE	49	16595	4	1	1	RNA-binding protein 3
RBM44_MOUSE	21	113978	2	1	1	RNA-binding protein 44
RCN1_MOUSE	464	38090	29	1	1	Reticulocalbin-1
REEP5_MOUSE	413	21265	23	1	1	Receptor expression-enhancing protein 5
RFX1_MOUSE	25	103985	1	1	1	MHC class II regulatory factor RFX1
RHG25_MOUSE	21	73679	1	1	1	Rho GTPase-activating protein 25
RHG26_MOUSE	28	92754	1	1	1	Rho GTPase-activating protein 26
RHOA_MOUSE	471	22110	32	5	1	Transforming protein RhoA
RHOC_MOUSE	180	22334	18	5	1	Rho-related GTP-binding protein RhoC
RIN3_MOUSE	94	108122	21	1	1	Ras and Rab interactor 3
RINI_MOUSE	46	51495	2	2	2	Ribonuclease inhibitor
RL10A_MOUSE	44	25072	5	2	2	60S ribosomal protein L10a
RL10L_MOUSE	164	24998	11	2	2	60S ribosomal protein L10-like
RL11_MOUSE	201	20468	25	3	3	60S ribosomal protein L11
RL12_MOUSE	784	17965	35	4	4	60S ribosomal protein L12
RL13_MOUSE	76	24348	3	2	2	60S ribosomal protein L13
RL17_MOUSE	72	21637	4	1	1	60S ribosomal protein L17
RL18_MOUSE	234	21688	15	3	3	60S ribosomal protein L18
RL18A_MOUSE	27	21004	1	1	1	60S ribosomal protein L18a
RL19_MOUSE	50	23565	2	1	1	60S ribosomal protein L19
RL22_MOUSE	213	14807	19	2	2	60S ribosomal protein L22
RL23_MOUSE	173	14970	16	2	2	60S ribosomal protein L23
RL23A_MOUSE	74	17684	3	2	2	60S ribosomal protein L23a
RL26_MOUSE	64	17248	3	2	2	60S ribosomal protein L26
RL27_MOUSE	64	15788	2	1	1	60S ribosomal protein L27
RL27A_MOUSE	59	16709	5	2	2	60S ribosomal protein L27a
RL3_MOUSE	82	46366	4	1	1	60S ribosomal protein L3
RL30_MOUSE	770	12947	36	3	3	60S ribosomal protein L30
RL31_MOUSE	65	14454	3	1	1	60S ribosomal protein L31
RL35_MOUSE	21	14544	2	1	1	60S ribosomal protein L35
RL36_MOUSE	72	12265	8	1	1	60S ribosomal protein L36
RL38_MOUSE	26	8256	1	1	1	60S ribosomal protein L38
RL4_MOUSE	305	47409	25	2	2	60S ribosomal protein L4
RL5_MOUSE	166	34607	14	2	2	60S ribosomal protein L5
RL7_MOUSE	487	31457	25	2	2	60S ribosomal protein L7
RL7A_MOUSE	93	30129	5	3	3	60S ribosomal protein L7a
RL8_MOUSE	135	28235	6	2	2	60S ribosomal protein L8
RL9_MOUSE	1628	21982	62	5	5	60S ribosomal protein L9
RLA0_MOUSE	1922	34366	115	9	9	60S acidic ribosomal protein P0
RLA1_MOUSE	541	11582	36	2	2	60S acidic ribosomal protein P1
RLA2_MOUSE	314	11644	24	4	4	60S acidic ribosomal protein P2
RM12_MOUSE	41	21809	1	1	1	39S ribosomal protein L12, mitochondrial
RN103_MOUSE	29	80165	3	1	1	E3 ubiquitin-protein ligase RNF103
RN208_MOUSE	63	29283	10	1	1	RING finger protein 208
ROA2_MOUSE	981	37437	47	7	7	Heterogeneous nuclear ribonucleoproteins A2/B1
ROA3_MOUSE	407	39856	22	5	5	Heterogeneous nuclear ribonucleoprotein A3
ROAA_MOUSE	20	30926	1	1	1	Heterogeneous nuclear ribonucleoprotein A/B
RPN1_MOUSE	419	68657	33	7	7	Dolichyl-diphosphooligosaccharide--protein glycosyltransferase subunit 1
RPN2_MOUSE	666	69134	32	3	3	Dolichyl-diphosphooligosaccharide--protein glycosyltransferase subunit 2
RRAGD_MOUSE	23	51656	1	1	1	Ras-related GTP-binding protein D
RRAS_MOUSE	135	23920	10	3	2	Ras-related protein R-Ras
RRAS2_MOUSE	122	23613	10	2	1	Ras-related protein R-Ras2
RS10_MOUSE	1500	18904	62	5	5	40S ribosomal protein S10
RS11_MOUSE	59	18590	10	3	3	40S ribosomal protein S11
RS12_MOUSE	26	14858	1	1	1	40S ribosomal protein S12
RS13_MOUSE	317	17212	26	3	3	40S ribosomal protein S13
RS14_MOUSE	463	16434	28	3	3	40S ribosomal protein S14
RS15_MOUSE	771	17029	31	3	3	40S ribosomal protein S15
RS15A_MOUSE	576	14944	31	4	4	40S ribosomal protein S15a

RS16_MOUSE	651	16549	52	5	5	40S ribosomal protein S16
RS17_MOUSE	92	15571	6	1	1	40S ribosomal protein S17
RS18_MOUSE	321	17708	24	7	7	40S ribosomal protein S18
RS19_MOUSE	264	16076	19	4	4	40S ribosomal protein S19
RS2_MOUSE	632	31497	29	4	4	40S ribosomal protein S2
RS20_MOUSE	293	13478	27	3	3	40S ribosomal protein S20
RS23_MOUSE	27	15969	2	1	1	40S ribosomal protein S23
RS24_MOUSE	133	15413	5	1	1	40S ribosomal protein S24
RS25_MOUSE	293	13791	20	4	4	40S ribosomal protein S25
RS26_MOUSE	62	13292	4	1	1	40S ribosomal protein S26
RS27A_MOUSE	2236	18282	105	4	4	Ubiquitin-40S ribosomal protein S27a
RS27L_MOUSE	40	9813	2	1	1	40S ribosomal protein S27-like
RS28_MOUSE	451	7893	27	3	3	40S ribosomal protein S28
RS3_MOUSE	1633	26828	121	12	12	40S ribosomal protein S3
RS30_MOUSE	63	6644	6	1	1	40S ribosomal protein S30
RS3A_MOUSE	129	30094	11	4	4	40S ribosomal protein S3a
RS4X_MOUSE	763	29807	45	5	5	40S ribosomal protein S4, X isoform
RS5_MOUSE	622	23046	42	5	5	40S ribosomal protein S5
RS6_MOUSE	50	28834	4	1	1	40S ribosomal protein S6
RS7_MOUSE	1119	22113	63	8	8	40S ribosomal protein S7
RS8_MOUSE	66	24475	6	3	3	40S ribosomal protein S8
RS9_MOUSE	86	22635	8	2	2	40S ribosomal protein S9
RSH6A_MOUSE	17	80493	1	1	1	Radial spoke head protein 6 homolog A
RSMB_MOUSE	23	23811	1	1	1	Small nuclear ribonucleoprotein-associated protein B
RSSA_MOUSE	3147	32931	126	7	7	40S ribosomal protein SA
RSU1_MOUSE	259	31531	23	4	4	Ras suppressor protein 1
RT30_MOUSE	228	50364	13	1	1	28S ribosomal protein S30, mitochondrial
RT34_MOUSE	19	25868	1	1	1	28S ribosomal protein S34, mitochondrial
RT36_MOUSE	34	11094	2	2	2	28S ribosomal protein S36, mitochondrial
RT4I1_MOUSE	115	43686	9	2	2	Reticulon-4-interacting protein 1, mitochondrial
RTCB_MOUSE	89	55727	4	2	2	tRNA-splicing ligase RtcB homolog
RTN1_MOUSE	28	83863	2	1	1	Reticulon-1
RTN2_MOUSE	9116	51543	234	7	7	Reticulon-2
RTN4_MOUSE	3563	127048	182	6	6	Reticulon-4
RTRAF_MOUSE	268	28249	5	1	1	RNA transcription, translation and transport factor protein
RYR1_MOUSE	54692	570381	2551	191	191	Ryanodine receptor 1
S10AA_MOUSE	18	11293	2	1	1	Protein S100-A10
S2551_MOUSE	18	33896	1	1	1	Solute carrier family 25 member 51
S27A1_MOUSE	27	72200	1	1	1	Long-chain fatty acid transport protein 1
S41A3_MOUSE	19	53793	1	1	1	Solute carrier family 41 member 3
S61A2_MOUSE	37	52670	2	1	1	Protein transport protein Sec61 subunit alpha isoform 2
SAC1_MOUSE	56	67471	2	1	1	Phosphatidylinositide phosphatase SAC1
SAC2_MOUSE	59	128782	6	1	1	Phosphatidylinositide phosphatase SAC2
SAHH_MOUSE	102	48170	9	3	3	Adenosylhomocysteinase
SALL4_MOUSE	19	114635	1	1	1	Sal-like protein 4
SAM50_MOUSE	2482	52230	127	12	12	Sorting and assembly machinery component 50 homolog
SAP_MOUSE	61	63263	5	1	1	Prosaposin
SAPC2_MOUSE	164	43209	26	1	1	Suppressor APC domain-containing protein 2
SAR1A_MOUSE	1269	22471	55	3	2	GTP-binding protein SAR1a
SAR1B_MOUSE	3224	22482	163	9	8	GTP-binding protein SAR1b
SBNO2_MOUSE	33	150455	1	1	1	Protein strawberry notch homolog 2
SC5AA_MOUSE	25	65348	1	1	1	Sodium/glucose cotransporter 5
SCN4B_MOUSE	28	25461	3	1	1	Sodium channel subunit beta-4
SCOT1_MOUSE	1899	56352	84	10	10	Succinyl-CoA:3-ketoacid coenzyme A transferase 1, mitochondrial
SCPDL_MOUSE	53	47669	3	2	2	Saccharopine dehydrogenase-like oxidoreductase
SCRN3_MOUSE	223	48030	13	3	3	Secernin-3
SDCG8_MOUSE	44	83726	2	1	1	Serologically defined colon cancer antigen 8 homolog
SDHA_MOUSE	14460	73623	686	32	32	Succinate dehydrogenase [ubiquinone] flavoprotein subunit, mitochondrial
SDHB_MOUSE	7707	32591	402	13	13	Succinate dehydrogenase [ubiquinone] iron-sulfur subunit, mitochondrial
SERPH_MOUSE	178	46618	11	3	3	Serpin H1
SFI1_MOUSE	63	145596	7	1	1	Protein SFI1 homolog
SFPQ_MOUSE	199	75508	11	1	1	Splicing factor, proline- and glutamine-rich
SFXN3_MOUSE	35	35669	1	1	1	Sideroflexin-3
SGCA_MOUSE	706	43659	53	8	8	Alpha-sarcoglycan
SGCB_MOUSE	51	35193	6	1	1	Beta-sarcoglycan
SGCD_MOUSE	171	32455	8	2	2	Delta-sarcoglycan
SGCG_MOUSE	137	32347	8	2	2	Gamma-sarcoglycan
SH3BG_MOUSE	1864	23146	135	10	10	SH3 domain-binding glutamic acid-rich protein
SIR2_MOUSE	19	43856	1	1	1	NAD-dependent protein deacetylase sirtuin-2
SKP1_MOUSE	84	18831	6	1	1	S-phase kinase-associated protein 1
SLIRP_MOUSE	19	12654	1	1	1	SRA stem-loop-interacting RNA-binding protein, mitochondrial
SLMAP_MOUSE	43	97729	2	2	2	Sarcolemmal membrane-associated protein
SMC6_MOUSE	30	128201	11	1	1	Structural maintenance of chromosomes protein 6
SMG7_MOUSE	17	127389	1	1	1	Protein SMG7
SMPX_MOUSE	75	9247	3	1	1	Small muscular protein
SMTL2_MOUSE	251	49778	12	3	3	Smoothelin-like protein 2
SMYD1_MOUSE	10336	57428	474	22	22	Histone-lysine N-methyltransferase Smyd1
SMYD2_MOUSE	70	50618	3	1	1	N-lysine methyltransferase SMYD2
SNAA_MOUSE	21	33624	1	1	1	Alpha-soluble NSF attachment protein
SND1_MOUSE	23	102709	1	1	1	Staphylococcal nuclease domain-containing protein 1
SNTA1_MOUSE	3113	54088	168	10	10	Alpha-1-syntrophin
SNX19_MOUSE	37	111107	1	1	1	Sorting nexin-19
SODC_MOUSE	1111	16104	48	2	2	Superoxide dismutase [Cu-Zn]

SODM_MOUSE	2710	24816	122	7	7	Superoxide dismutase [Mn], mitochondrial
SOGA1_MOUSE	40	160627	1	1	1	Protein SOGA1
SORC1_MOUSE	31	130690	2	1	1	VPS10 domain-containing receptor SorCS1
SPA3K_MOUSE	3447	47021	188	16	16	Serine protease inhibitor A3K
SPB6_MOUSE	1684	42913	102	12	12	Serpin B6
SPCS_MOUSE	80	56032	7	1	1	O-phosphoseryl-tRNA(Sec) selenium transferase
SPEG_MOUSE	914	356975	89	25	25	Striated muscle-specific serine/threonine-protein kinase
SPI2_MOUSE	33	45861	1	1	1	Serpin I2
SPICE_MOUSE	18	95929	1	1	1	Spindle and centriole-associated protein 1
SPN90_MOUSE	61	79207	4	1	1	NCK-interacting protein with SH3 domain
SPO11_MOUSE	56	44940	6	1	1	Meiotic recombination protein SPO11
SPRE_MOUSE	157	28208	13	3	3	Sepiapterin reductase
SPRY4_MOUSE	57	23374	5	1	1	SPRY domain-containing protein 4
SPSB3_MOUSE	20	40208	1	1	1	SPRY domain-containing SOCS box protein 3
SPTA1_MOUSE	26	280833	2	2	2	Spectrin alpha chain, erythrocytic 1
SPTB1_MOUSE	888	245897	52	10	10	Spectrin beta chain, erythrocytic
SPTB2_MOUSE	1141	274908	69	12	12	Spectrin beta chain, non-erythrocytic 1
SPTN1_MOUSE	2539	285221	98	18	18	Spectrin alpha chain, non-erythrocytic 1
SQOR_MOUSE	28	50706	1	1	1	Sulfide:quinone oxidoreductase, mitochondrial
SRBS1_MOUSE	43	143952	1	1	1	Sorbin and SH3 domain-containing protein 1
SRCA_MOUSE	53463	99522	1923	42	42	Sarcalumenin
SRPRA_MOUSE	22	70035	1	1	1	Signal recognition particle receptor subunit alpha
SRPRB_MOUSE	22	29732	1	1	1	Signal recognition particle receptor subunit beta
SRSF4_MOUSE	42	56061	2	1	1	Serine/arginine-rich splicing factor 4
SSNA1_MOUSE	34	13720	1	1	1	Sjoegren syndrome nuclear autoantigen 1 homolog
SSPN_MOUSE	27	24640	1	1	1	Sarcospan
SSPO_MOUSE	20	565916	1	1	1	SCO-spondin
SSRD_MOUSE	38	19153	3	1	1	Translocon-associated protein subunit delta
STAC3_MOUSE	321	41317	31	8	8	SH3 and cysteine-rich domain-containing protein 3
STAT3_MOUSE	21	88796	1	1	1	Signal transducer and activator of transcription 3
STBD1_MOUSE	416	36219	25	5	5	Starch-binding domain-containing protein 1
STIM1_MOUSE	88	77803	11	3	3	Stromal interaction molecule 1
STIP1_MOUSE	69	63170	7	3	3	Stress-induced-phosphoprotein 1
STML2_MOUSE	25	38475	1	1	1	Stomatin-like protein 2, mitochondrial
SUCA_MOUSE	3223	36474	189	8	8	Succinate--CoA ligase [ADP/GDP-forming] subunit alpha, mitochondrial
SUCB1_MOUSE	9452	50424	406	21	21	Succinate--CoA ligase [ADP-forming] subunit beta, mitochondrial
SUCB2_MOUSE	47	47096	4	3	3	Succinate--CoA ligase [GDP-forming] subunit beta, mitochondrial
SUOX_MOUSE	26	61231	1	1	1	Sulfite oxidase, mitochondrial
SURF4_MOUSE	682	30589	21	2	2	Surfeit locus protein 4
SYAC_MOUSE	24	107697	2	1	1	Alanine--tRNA ligase, cytoplasmic
SYDC_MOUSE	180	57567	17	4	4	Aspartate--tRNA ligase, cytoplasmic
SYEP_MOUSE	461	171739	27	3	3	Bifunctional glutamate/proline--tRNA ligase
SYFB_MOUSE	36	66340	5	1	1	Phenylalanine--tRNA ligase beta subunit
SYIM_MOUSE	324	113873	16	2	2	Isoleucine--tRNA ligase, mitochondrial
SYNE2_MOUSE	80	787997	7	1	1	Nesprin-2
SYNEM_MOUSE	5966	173276	306	38	38	Synemin
SYNP2_MOUSE	5107	116968	220	13	13	Synaptopodin-2
SYNPO_MOUSE	108	99947	4	3	3	Synaptopodin
SYP2L_MOUSE	2782	103175	161	14	14	Synaptopodin 2-like protein
SYPL1_MOUSE	83	29223	7	1	1	Synaptophysin-like protein 1
SYPL2_MOUSE	2754	29605	88	4	4	Synaptophysin-like protein 2
SYT16_MOUSE	444	71894	40	1	1	Synaptotagmin-16
SYTC2_MOUSE	40	92173	2	1	1	Probable threonine--tRNA ligase 2, cytoplasmic
SYVC_MOUSE	224	141439	12	3	3	Valine--tRNA ligase
SYWC_MOUSE	26	54893	1	1	1	Tryptophan--tRNA ligase, cytoplasmic
SYYC_MOUSE	79	59410	5	3	3	Tyrosine--tRNA ligase, cytoplasmic
T126A_MOUSE	92	21640	5	2	2	Transmembrane protein 126A
TACT_MOUSE	17	67888	1	1	1	T-cell surface protein tactile
TAGL_MOUSE	107	22618	8	3	3	Transgelin
TALDO_MOUSE	91	37534	3	1	1	Transaldolase
TAP2_MOUSE	19	77852	1	1	1	Antigen peptide transporter 2
TBA1A_MOUSE	7760	50788	359	21	6	Tubulin alpha-1A chain
TBA4A_MOUSE	12697	50634	492	22	6	Tubulin alpha-4A chain
TBA8_MOUSE	6499	50704	225	14	2	Tubulin alpha-8 chain
TBB2B_MOUSE	7926	50377	287	18	1	Tubulin beta-2B chain
TBB3_MOUSE	4322	50842	171	13	1	Tubulin beta-3 chain
TBB4B_MOUSE	11050	50255	451	24	4	Tubulin beta-4B chain
TBB5_MOUSE	7578	50095	291	22	3	Tubulin beta-5 chain
TBB6_MOUSE	4269	50514	160	12	2	Tubulin beta-6 chain
TCPA_MOUSE	74	60867	5	3	3	T-complex protein 1 subunit alpha
TCPB_MOUSE	208	57783	23	3	3	T-complex protein 1 subunit beta
TCPD_MOUSE	463	58543	24	4	4	T-complex protein 1 subunit delta
TCPE_MOUSE	103	60042	7	2	2	T-complex protein 1 subunit epsilon
TCPG_MOUSE	48	61162	2	2	2	T-complex protein 1 subunit gamma
TCPH_MOUSE	472	60127	43	8	8	T-complex protein 1 subunit eta
TCPQ_MOUSE	78	60088	6	3	3	T-complex protein 1 subunit theta
TCPZ_MOUSE	1703	58424	57	5	5	T-complex protein 1 subunit zeta
TCTP_MOUSE	1720	19564	98	6	6	Translationally-controlled tumor protein
TDR12_MOUSE	25	139876	1	1	1	Putative ATP-dependent RNA helicase TDRD12
TEAD3_MOUSE	18	49078	1	1	1	Transcriptional enhancer factor TEF-5
TEC_MOUSE	23	74291	2	1	1	Tyrosine-protein kinase Tec
TECR_MOUSE	781	36466	36	5	5	Very-long-chain enoyl-CoA reductase
TEKT2_MOUSE	18	50736	1	1	1	Tektin-2

TELT_MOUSE	1107	19294	79	6	6	Telethonin
TENA_MOUSE	40	237304	5	3	3	Tenascin
TERA_MOUSE	7175	89950	396	31	31	Transitional endoplasmic reticulum ATPase
TET1_MOUSE	41	222431	5	1	1	Methylcytosine dioxygenase TET1
TGM2_MOUSE	506	78153	38	9	9	Protein-glutamine gamma-glutamyltransferase 2
THIKA_MOUSE	68	44382	3	1	1	3-ketoacyl-CoA thiolase A, peroxisomal
THIL_MOUSE	6260	45129	307	13	13	Acetyl-CoA acetyltransferase, mitochondrial
THIM_MOUSE	6351	42260	309	17	17	3-ketoacyl-CoA thiolase, mitochondrial
THIO_MOUSE	275	12010	12	1	1	Thioredoxin
THS7B_MOUSE	23	186774	1	1	1	Thrombospondin type-1 domain-containing protein 7B
THTM_MOUSE	555	33305	23	4	4	3-mercaptopyruvate sulfurtransferase
TIGAR_MOUSE	134	29571	10	2	2	Fructose-2,6-bisphosphatase TIGAR
TIM13_MOUSE	73	10679	4	1	1	Mitochondrial import inner membrane translocase subunit Tim13
TIM50_MOUSE	577	39980	22	2	2	Mitochondrial import inner membrane translocase subunit TIM50
TINAL_MOUSE	28	53998	1	1	1	Tubulointerstitial nephritis antigen-like
TITIN_MOUSE	1856619	3933362	80795	2058	2057	Titin
TLN1_MOUSE	760	271820	37	11	10	Talin-1
TLN2_MOUSE	587	255743	36	7	6	Talin-2
TLR11_MOUSE	28	107231	1	1	1	Toll-like receptor 11
TM109_MOUSE	135	26346	11	3	3	Transmembrane protein 109
TM145_MOUSE	23	83390	1	1	1	Transmembrane protein 145
TM214_MOUSE	24	77407	2	1	1	Transmembrane protein 214
TM256_MOUSE	21	11762	1	1	1	Transmembrane protein 256
TM38A_MOUSE	3980	33686	159	10	10	Trimeric intracellular cation channel type A
TM38B_MOUSE	23	32904	1	1	1	Trimeric intracellular cation channel type B
TM9S1_MOUSE	31	69454	4	1	1	Transmembrane 9 superfamily member 1
TMCO1_MOUSE	138	21389	8	1	1	Calcium load-activated calcium channel
TMEDA_MOUSE	47	25066	6	1	1	Transmembrane emp24 domain-containing protein 10
TMF1_MOUSE	19	122242	1	1	1	TATA element modulatory factor
TMM11_MOUSE	102	21754	11	1	1	Transmembrane protein 11, mitochondrial
TMM65_MOUSE	29	25187	1	1	1	Transmembrane protein 65
TMOD1_MOUSE	2533	40555	169	10	9	Tropomodulin-1
TMOD4_MOUSE	7346	39579	345	17	16	Tropomodulin-4
TMX2_MOUSE	47	34320	1	1	1	Thioredoxin-related transmembrane protein 2
TMX3_MOUSE	22	52157	1	1	1	Protein disulfide-isomerase TMX3
TMX4_MOUSE	59	37507	2	1	1	Thioredoxin-related transmembrane protein 4
TNMD_MOUSE	23	37822	1	1	1	Tenomodulin
TNNC1_MOUSE	442	18523	19	1	1	Troponin C, slow skeletal and cardiac muscles
TNNC2_MOUSE	21498	18155	803	12	11	Troponin C, skeletal muscle
TNNI1_MOUSE	2445	21856	96	7	7	Troponin I, slow skeletal muscle
TNNI2_MOUSE	34424	21515	1417	12	12	Troponin I, fast skeletal muscle
TNNT1_MOUSE	2416	31325	113	10	8	Troponin T, slow skeletal muscle
TNNT3_MOUSE	58863	32221	2012	20	18	Troponin T, fast skeletal muscle
TNS2_MOUSE	19	153856	1	1	1	Tensin-2
TOM40_MOUSE	750	38270	23	1	1	Mitochondrial import receptor subunit TOM40 homolog
TOM70_MOUSE	92	68288	8	1	1	Mitochondrial import receptor subunit TOM70
TOP1M_MOUSE	38	69701	2	2	2	DNA topoisomerase I, mitochondrial
TPIS_MOUSE	22155	32684	869	16	16	Triosephosphate isomerase
TPM1_MOUSE	46179	32718	1891	42	22	Tropomyosin alpha-1 chain
TPM2_MOUSE	33341	32931	1380	36	24	Tropomyosin beta chain
TPM3_MOUSE	18026	33031	818	28	12	Tropomyosin alpha-3 chain
TPP1_MOUSE	103	61760	7	2	2	Tripeptidyl-peptidase 1
TPP2_MOUSE	18	141330	1	1	1	Tripeptidyl-peptidase 2
TPX2_MOUSE	20	86297	1	1	1	Targeting protein for Xklp2
TR109_MOUSE	24	37068	1	1	1	Taste receptor type 2 member 109
TRAP1_MOUSE	416	80501	11	2	2	Heat shock protein 75 kDa, mitochondrial
TRDN_MOUSE	1956	77967	116	11	11	Triadin
TRFE_MOUSE	8787	78841	480	37	37	Serotransferrin
TRI46_MOUSE	19	85203	1	1	1	Tripartite motif-containing protein 46
TRI59_MOUSE	29	48120	1	1	1	Tripartite motif-containing protein 59
TRI72_MOUSE	8874	53695	342	18	18	Tripartite motif-containing protein 72
TRPC1_MOUSE	24	92237	1	1	1	Short transient receptor potential channel 1
TRPC2_MOUSE	39	131645	4	1	1	Short transient receptor potential channel 2
TRXR1_MOUSE	49	68380	2	2	2	Thioredoxin reductase 1, cytoplasmic
TSN7_MOUSE	27	28153	1	1	1	Tetraspanin-7
TSN8_MOUSE	25	26249	1	1	1	Tetraspanin-8
TSP4_MOUSE	486	108979	37	14	13	Thrombospondin-4
TTHY_MOUSE	141	15880	9	3	3	Transthyretin
TUFT1_MOUSE	45	44720	5	1	1	Tuftelin
TWF2_MOUSE	195	39674	15	1	1	Twinfilin-2
TXNL1_MOUSE	31	32616	1	1	1	Thioredoxin-like protein 1
TXTP_MOUSE	31	34252	2	1	1	Tricarboxylate transport protein, mitochondrial
TYDP1_MOUSE	29	69046	1	1	1	Tyrosyl-DNA phosphodiesterase 1
UB2D2_MOUSE	289	16953	18	2	2	Ubiquitin-conjugating enzyme E2 D2
UB2L3_MOUSE	739	18021	22	2	2	Ubiquitin-conjugating enzyme E2 L3
UB2V1_MOUSE	266	16458	15	4	4	Ubiquitin-conjugating enzyme E2 variant 1
UBA1_MOUSE	1531	118931	79	10	10	Ubiquitin-like modifier-activating enzyme 1
UBE2N_MOUSE	696	17184	63	5	5	Ubiquitin-conjugating enzyme E2 N
UBP15_MOUSE	86	113508	5	1	1	Ubiquitin carboxyl-terminal hydrolase 15
UBP5_MOUSE	168	96685	11	2	2	Ubiquitin carboxyl-terminal hydrolase 5
UBR3_MOUSE	31	216211	1	1	1	E3 ubiquitin-protein ligase UBR3
UCP3_MOUSE	159	34288	8	2	2	Mitochondrial uncoupling protein 3
UCRI_MOUSE	5330	29634	319	13	13	Cytochrome b-c1 complex subunit Rieske, mitochondrial

UGGG1_MOUSE	182	177007	21	3	3	UDP-glucose:glycoprotein glucosyltransferase 1
UGPA_MOUSE	5705	57115	258	22	22	UTP--glucose-1-phosphate uridylyltransferase
UHRF1_MOUSE	57	89673	9	1	1	E3 ubiquitin-protein ligase UHRF1
UN45B_MOUSE	1390	104884	64	8	8	Protein unc-45 homolog B
UNC79_MOUSE	21	295057	1	1	1	Protein unc-79 homolog
UROL1_MOUSE	19	147794	1	1	1	Uromodulin-like 1
USMG5_MOUSE	1296	6434	56	3	3	Up-regulated during skeletal muscle growth protein 5
USO1_MOUSE	37	108000	1	1	1	General vesicular transport factor p115
USP9X_MOUSE	41	294061	3	1	1	Probable ubiquitin carboxyl-terminal hydrolase FAF-X
VAMP1_MOUSE	88	12996	3	1	1	Vesicle-associated membrane protein 1
VANG1_MOUSE	20	60311	1	1	1	Vang-like protein 1
VAPA_MOUSE	1454	28065	81	7	6	Vesicle-associated membrane protein-associated protein A
VAPB_MOUSE	2184	27157	113	6	5	Vesicle-associated membrane protein-associated protein B
VAT1_MOUSE	198	43297	8	1	1	Synaptic vesicle membrane protein VAT-1 homolog
VDAC1_MOUSE	40277	32502	1339	19	19	Voltage-dependent anion-selective channel protein 1
VDAC2_MOUSE	6893	32340	333	14	14	Voltage-dependent anion-selective channel protein 2
VDAC3_MOUSE	11344	31076	463	12	12	Voltage-dependent anion-selective channel protein 3
VIGLN_MOUSE	39	142225	1	1	1	Vigilin
VIME_MOUSE	9622	53712	500	32	24	Vimentin
VINC_MOUSE	1303	117215	99	20	20	Vinculin
VP13A_MOUSE	18	360940	1	1	1	Vacuolar protein sorting-associated protein 13A
VP13B_MOUSE	19	448979	1	1	1	Vacuolar protein sorting-associated protein 13B
VP13C_MOUSE	35	422162	3	1	1	Vacuolar protein sorting-associated protein 13C
VPP2_MOUSE	35	99279	1	1	1	V-type proton ATPase 116 kDa subunit a isoform 2
VSIG8_MOUSE	18	45242	1	1	1	V-set and immunoglobulin domain-containing protein 8
VTDB_MOUSE	1525	55162	70	8	8	Vitamin D-binding protein
VWA1_MOUSE	28	44795	2	2	2	von Willebrand factor A domain-containing protein 1
WDR1_MOUSE	519	67049	42	9	9	WD repeat-containing protein 1
WFS1_MOUSE	40	101597	3	2	2	Wolframin
WN10A_MOUSE	110	47793	17	1	1	Protein Wnt-10a
XIRP1_MOUSE	291	124096	14	2	2	Xin actin-binding repeat-containing protein 1
XPO2_MOUSE	286	111183	43	2	2	Exportin-2
XPO5_MOUSE	221	139050	42	1	1	Exportin-5
XXLT1_MOUSE	218	44266	32	1	1	Xyloside xylosyltransferase 1
YBOX3_MOUSE	129	38790	8	3	3	Y-box-binding protein 3
YJEN3_MOUSE	32	27455	1	1	1	YjeF N-terminal domain-containing protein 3
ZBED3_MOUSE	26	25878	1	1	1	Zinc finger BED domain-containing protein 3
ZFY27_MOUSE	28	47483	3	1	1	Protrudin
ZHX2_MOUSE	18	92772	1	1	1	Zinc fingers and homeoboxes protein 2
ZN692_MOUSE	31	60228	2	1	1	Zinc finger protein 692
ZSC21_MOUSE	27	64170	1	1	1	Zinc finger and SCAN domain-containing protein 21
**GuHCl extract**						
1433E_MOUSE	1107	29326	75	7	4	14-3-3 protein epsilon
1433G_MOUSE	1116	28456	84	9	6	14-3-3 protein gamma
1433S_MOUSE	669	27803	50	4	1	14-3-3 protein sigma
2ABA_MOUSE	43	52173	2	1	1	Serine/threonine-protein phosphatase 2A 55 kDa regulatory subunit B alpha isoform
3HIDH_MOUSE	52	35816	4	1	1	3-hydroxyisobutyrate dehydrogenase, mitochondrial
68MP_MOUSE	329	6694	21	1	1	6.8 kDa mitochondrial proteolipid
A1AT3_MOUSE	465	45966	38	9	9	Alpha-1-antitrypsin 1-3
AAPK2_MOUSE	163	62610	14	2	2	5'-AMP-activated protein kinase catalytic subunit alpha-2
AATC_MOUSE	2146	46504	118	13	13	Aspartate aminotransferase, cytoplasmic
AATM_MOUSE	6174	47780	271	20	20	Aspartate aminotransferase, mitochondrial
ABCC9_MOUSE	41	175495	2	2	2	ATP-binding cassette sub-family C member 9
ABCF2_MOUSE	48	72306	2	1	1	ATP-binding cassette sub-family F member 2
ABEC2_MOUSE	2111	25872	108	8	8	C->U-editing enzyme APOBEC-2
ACAD8_MOUSE	85	45789	3	1	1	Isobutyryl-CoA dehydrogenase, mitochondrial
ACADL_MOUSE	1496	48277	69	8	8	Long-chain specific acyl-CoA dehydrogenase, mitochondrial
ACADM_MOUSE	740	46908	40	8	8	Medium-chain specific acyl-CoA dehydrogenase, mitochondrial
ACADV_MOUSE	3915	71230	172	17	17	Very long-chain specific acyl-CoA dehydrogenase, mitochondrial
ACDSB_MOUSE	573	48300	12	2	2	Short/branched chain specific acyl-CoA dehydrogenase, mitochondrial
ACON_MOUSE	11909	86151	568	33	33	Aconitate hydratase, mitochondrial
ACSL1_MOUSE	3129	78928	175	15	15	Long-chain-fatty-acid--CoA ligase 1
ACSL3_MOUSE	21	81467	1	1	1	Long-chain-fatty-acid--CoA ligase 3
ACTB_MOUSE	37166	42052	1789	23	8	Actin, cytoplasmic 1
ACTBL_MOUSE	21084	42319	828	13	5	Beta-actin-like protein 2
ACTH_MOUSE	84999	42249	3733	31	4	Actin, gamma-enteric smooth muscle
ACTN1_MOUSE	16708	103631	736	24	3	Alpha-actinin-1
ACTN2_MOUSE	57003	104339	2302	62	40	Alpha-actinin-2
ACTN3_MOUSE	76733	103605	3041	74	57	Alpha-actinin-3
ACTN4_MOUSE	14087	105368	645	20	3	Alpha-actinin-4
ACTS_MOUSE	119329	42366	4804	35	7	Actin, alpha skeletal muscle
ADA22_MOUSE	276	102331	20	1	1	Disintegrin and metalloproteinase domain-containing protein 22
ADIPO_MOUSE	50	26906	2	2	2	Adiponectin
ADT1_MOUSE	19311	33111	971	18	9	ADP/ATP translocase 1
ADT2_MOUSE	10111	33138	498	10	1	ADP/ATP translocase 2
ADT4_MOUSE	2627	35521	126	4	1	ADP/ATP translocase 4
AGAP1_MOUSE	32	95322	5	1	1	Arf-GAP with GTPase, ANK repeat and PH domain-containing protein 1
AGRIN_MOUSE	30	215386	5	1	1	Agrin
AIFM1_MOUSE	294	66952	21	6	6	Apoptosis-inducing factor 1, mitochondrial
AKAP9_MOUSE	29	439138	4	1	1	A-kinase anchor protein 9
AL1A1_MOUSE	23	55060	1	1	1	Retinal dehydrogenase 1
AL3A1_MOUSE	54	50848	2	2	2	Aldehyde dehydrogenase, dimeric NADP-preferring
AL4A1_MOUSE	43	62258	4	2	2	Delta-1-pyrroline-5-carboxylate dehydrogenase, mitochondrial

ALBU_MOUSE	13854	70700	633	32	32	Serum albumin
ALDH2_MOUSE	150	57015	13	6	6	Aldehyde dehydrogenase, mitochondrial
ALDOA_MOUSE	27169	39787	1204	31	7	Fructose-bisphosphate aldolase A
ALDOC_MOUSE	4932	39769	320	7	3	Fructose-bisphosphate aldolase C
ALDR_MOUSE	850	36052	38	6	6	Aldose reductase
ALRF2_MOUSE	68	23773	5	1	1	Aly/REF export factor 2
AMPD1_MOUSE	4957	86734	278	22	22	AMP deaminase 1
ANGL7_MOUSE	25	39502	1	1	1	Angiopoietin-related protein 7
ANK1_MOUSE	172	205355	14	2	2	Ankyrin-1
ANKR2_MOUSE	340	36855	9	3	3	Ankyrin repeat domain-containing protein 2
ANX11_MOUSE	317	54387	19	5	5	Annexin A11
ANXA2_MOUSE	459	38937	25	6	6	Annexin A2
ANXA6_MOUSE	1126	76294	53	11	11	Annexin A6
APC4_MOUSE	21	92448	1	1	1	Anaphase-promoting complex subunit 4
APOA1_MOUSE	385	30597	20	4	4	Apolipoprotein A-I
APOH_MOUSE	29	39904	1	1	1	Beta-2-glycoprotein 1
AQP1_MOUSE	529	29003	27	4	4	Aquaporin-1
ARAP3_MOUSE	36	171573	10	1	1	Arf-GAP with Rho-GAP domain, ANK repeat and PH domain-containing protein 3
ARF1_MOUSE	45	20741	1	1	1	ADP-ribosylation factor 1
ARFG2_MOUSE	34	56905	1	1	1	ADP-ribosylation factor GTPase-activating protein 2
ARHGH_MOUSE	20	223757	1	1	1	Rho guanine nucleotide exchange factor 17
ARP2_MOUSE	31	45017	2	1	1	Actin-related protein 2
ARP3B_MOUSE	250	48062	8	1	1	Actin-related protein 3B
ASPH_MOUSE	179	83390	10	1	1	Aspartyl/asparaginyl beta-hydroxylase
ASPN_MOUSE	2137	42944	116	13	13	Asporin
AT1A1_MOUSE	1194	114221	80	12	3	Sodium/potassium-transporting ATPase subunit alpha-1
AT1A2_MOUSE	3831	113457	235	25	16	Sodium/potassium-transporting ATPase subunit alpha-2
AT1B1_MOUSE	221	35571	8	2	2	Sodium/potassium-transporting ATPase subunit beta-1
AT1B2_MOUSE	203	33722	9	1	1	Sodium/potassium-transporting ATPase subunit beta-2
AT2A1_MOUSE	157268	110723	5544	67	43	Sarcoplasmic/endoplasmic reticulum calcium ATPase 1
AT2A2_MOUSE	53400	116437	1926	34	14	Sarcoplasmic/endoplasmic reticulum calcium ATPase 2
AT2A3_MOUSE	25785	114991	938	17	2	Sarcoplasmic/endoplasmic reticulum calcium ATPase 3
AT5F1_MOUSE	2471	29044	139	7	7	ATP synthase F(0) complex subunit B1, mitochondrial
AT5G1_MOUSE	28	14362	2	1	1	ATP synthase F(0) complex subunit C1, mitochondrial
ATAD1_MOUSE	27	41060	1	1	1	ATPase family AAA domain-containing protein 1
ATG9B_MOUSE	39	102762	1	1	1	Autophagy-related protein 9B
ATP5E_MOUSE	197	5891	11	2	2	ATP synthase subunit epsilon, mitochondrial
ATP5H_MOUSE	821	18795	49	4	4	ATP synthase subunit d, mitochondrial
ATP5I_MOUSE	421	8230	14	1	1	ATP synthase subunit e, mitochondrial
ATP5L_MOUSE	556	11417	24	1	1	ATP synthase subunit g, mitochondrial
ATP8_MOUSE	29	7761	3	1	1	ATP synthase protein 8
ATPA_MOUSE	29016	59830	934	27	27	ATP synthase subunit alpha, mitochondrial
ATPB_MOUSE	29929	56265	1128	27	27	ATP synthase subunit beta, mitochondrial
ATPD_MOUSE	671	17589	24	2	2	ATP synthase subunit delta, mitochondrial
ATPG_MOUSE	3493	32979	191	11	11	ATP synthase subunit gamma, mitochondrial
ATPK_MOUSE	1274	10394	49	2	2	ATP synthase subunit f, mitochondrial
ATPO_MOUSE	4065	23406	132	10	10	ATP synthase subunit O, mitochondrial
B2L13_MOUSE	106	46862	6	1	1	Bcl-2-like protein 13
B3AT_MOUSE	77	103412	3	1	1	Band 3 anion transport protein
B3GN3_MOUSE	32	43072	1	1	1	N-acetyllactosaminide beta-1,3-N-acetylglucosaminyltransferase 3
BASI_MOUSE	473	42874	19	3	3	Basigin
BDH_MOUSE	106	38617	7	2	2	D-beta-hydroxybutyrate dehydrogenase, mitochondrial
BGH3_MOUSE	174	75177	13	6	6	Transforming growth factor-beta-induced protein ig-h3
BIN1_MOUSE	1032	64658	61	8	8	Myc box-dependent-interacting protein 1
BIP_MOUSE	1753	72492	104	11	9	Endoplasmic reticulum chaperone BiP
BRSK2_MOUSE	24	82309	2	1	1	Serine/threonine-protein kinase BRSK2
C11B2_MOUSE	28	57507	1	1	1	Cytochrome P450 11B2, mitochondrial
C1QBP_MOUSE	43	31336	2	1	1	Complement component 1 Q subcomponent-binding protein, mitochondrial
C560_MOUSE	279	18598	22	3	3	Succinate dehydrogenase cytochrome b560 subunit, mitochondrial
CA2D1_MOUSE	1196	125692	56	11	11	Voltage-dependent calcium channel subunit alpha-2/delta-1
CAC1A_MOUSE	47	269244	4	1	1	Voltage-dependent P/Q-type calcium channel subunit alpha-1A
CAC1S_MOUSE	2493	215402	134	15	15	Voltage-dependent L-type calcium channel subunit alpha-1S
CACB1_MOUSE	582	65731	31	6	6	Voltage-dependent L-type calcium channel subunit beta-1
CACP_MOUSE	288	71250	24	8	8	Carnitine O-acetyltransferase
CAD13_MOUSE	130	78536	10	3	3	Cadherin-13
CAH3_MOUSE	4312	29633	220	13	13	Carbonic anhydrase 3
CALM1_MOUSE	73	16827	8	4	3	Calmodulin-1
CALU_MOUSE	41	37155	1	1	1	Calumenin
CALX_MOUSE	36	67635	1	1	1	Calnexin
CAND2_MOUSE	635	137086	29	3	3	Cullin-associated NEDD8-dissociated protein 2
CAP1_MOUSE	47	51875	2	1	1	Adenylyl cyclase-associated protein 1
CAP2_MOUSE	107	53114	8	2	2	Adenylyl cyclase-associated protein 2
CAPZB_MOUSE	1100	31611	69	8	8	F-actin-capping protein subunit beta
CASQ1_MOUSE	15423	46349	733	18	18	Calsequestrin-1
CASQ2_MOUSE	416	48203	15	3	3	Calsequestrin-2
CATB_MOUSE	21	38168	1	1	1	Cathepsin B
CATD_MOUSE	24	45381	1	1	1	Cathepsin D
CAV1_MOUSE	366	20697	20	2	2	Caveolin-1
CAVN1_MOUSE	1269	43927	51	4	4	Caveolae-associated protein 1
CAVN4_MOUSE	48	41040	6	2	2	Caveolae-associated protein 4
CAZA2_MOUSE	2070	33118	89	7	7	F-actin-capping protein subunit alpha-2
CCD13_MOUSE	39	79815	6	2	2	Coiled-coil domain-containing protein 13
CCG1_MOUSE	209	25617	13	1	1	Voltage-dependent calcium channel gamma-1 subunit

CCG6_MOUSE	51	28697	2	1	1	Voltage-dependent calcium channel gamma-6 subunit
CD151_MOUSE	32	29083	1	1	1	CD151 antigen
CD158_MOUSE	30	127318	2	1	1	Coiled-coil domain-containing protein 158
CD36_MOUSE	432	53234	20	3	3	Platelet glycoprotein 4
CDIPT_MOUSE	59	23925	5	1	1	CDP-diacylglycerol--inositol 3-phosphatidyltransferase
CE162_MOUSE	37	161156	1	1	1	Centrosomal protein of 162 kDa
CENPV_MOUSE	94	27980	8	2	2	Centromere protein V
CG057_MOUSE	35	32932	2	1	1	Uncharacterized protein C7orf57 homolog
CH60_MOUSE	1375	61088	74	11	11	60 kDa heat shock protein, mitochondrial
CHADL_MOUSE	42	82450	3	1	1	Chondroadherin-like protein
CHCH2_MOUSE	24	15879	1	1	1	Coiled-coil-helix-coiled-coil-helix domain-containing protein 2
CHERP_MOUSE	93	106501	19	1	1	Calcium homeostasis endoplasmic reticulum protein
CILP1_MOUSE	1246	134530	55	7	7	Cartilage intermediate layer protein 1
CILP2_MOUSE	1903	128078	91	12	12	Cartilage intermediate layer protein 2
CING_MOUSE	37	136593	2	1	1	Cingulin
CIPC_MOUSE	20	46633	1	1	1	CLOCK-interacting pacemaker
CISD1_MOUSE	337	12260	21	3	3	CDGSH iron-sulfur domain-containing protein 1
CISY_MOUSE	5665	51988	228	12	12	Citrate synthase, mitochondrial
CLH1_MOUSE	515	193202	26	8	8	Clathrin heavy chain 1
CMA1_MOUSE	294	27968	18	4	4	Chymase
CMBL_MOUSE	31	28226	1	1	1	Carboxymethylenebutenolidase homolog
CMC1_MOUSE	10998	74922	478	30	30	Calcium-binding mitochondrial carrier protein Aralar1
CMYA5_MOUSE	35	414327	3	2	2	Cardiomyopathy-associated protein 5
CN37_MOUSE	89	47493	4	2	2	2',3'-cyclic-nucleotide 3'-phosphodiesterase
CO1A1_MOUSE	8505	138974	450	16	16	Collagen alpha-1(I) chain
CO1A2_MOUSE	12051	129992	432	13	13	Collagen alpha-2(I) chain
CO2A1_MOUSE	137	142970	12	4	4	Collagen alpha-1(II) chain
CO3_MOUSE	21	187905	2	1	1	Complement C3
CO3A1_MOUSE	286	140112	22	3	3	Collagen alpha-1(III) chain
CO4A1_MOUSE	5880	161719	233	8	8	Collagen alpha-1(IV) chain
CO4A2_MOUSE	4063	168417	179	11	11	Collagen alpha-2(IV) chain
CO5A1_MOUSE	198	184248	19	2	2	Collagen alpha-1(V) chain
CO5A2_MOUSE	68	145955	4	3	3	Collagen alpha-2(V) chain
CO6A1_MOUSE	9634	109562	446	21	21	Collagen alpha-1(VI) chain
CO6A2_MOUSE	6924	111406	373	22	22	Collagen alpha-2(VI) chain
CO6A6_MOUSE	38	247937	2	2	2	Collagen alpha-6(VI) chain
COBL_MOUSE	37	144518	2	1	1	Protein cordon-bleu
COCA1_MOUSE	6805	341202	406	51	51	Collagen alpha-1(XII) chain
COEA1_MOUSE	39	194089	1	1	1	Collagen alpha-1(XIV) chain
COF2_MOUSE	245	18812	24	3	3	Cofilin-2
COFA1_MOUSE	989	140841	75	8	8	Collagen alpha-1(XV) chain
COIA1_MOUSE	40	183085	2	1	1	Collagen alpha-1(XVIII) chain
COMP_MOUSE	1184	84912	74	15	13	Cartilage oligomeric matrix protein
COQ5_MOUSE	41	37711	1	1	1	2-methoxy-6-polyprenyl-1,4-benzoquinol methylase, mitochondrial
COQ8A_MOUSE	660	72154	29	5	5	Atypical kinase COQ8A, mitochondrial
COQ9_MOUSE	319	35232	21	3	3	Ubiquinone biosynthesis protein COQ9, mitochondrial
COR1B_MOUSE	46	54505	3	1	1	Coronin-1B
CORO6_MOUSE	70	53022	7	1	1	Coronin-6
COX1_MOUSE	119	56929	12	2	2	Cytochrome c oxidase subunit 1
COX2_MOUSE	3886	26130	182	5	5	Cytochrome c oxidase subunit 2
COX20_MOUSE	30	13383	1	1	1	Cytochrome c oxidase assembly protein COX20, mitochondrial
COX41_MOUSE	4627	19575	231	7	7	Cytochrome c oxidase subunit 4 isoform 1, mitochondrial
COX5A_MOUSE	155	16319	4	1	1	Cytochrome c oxidase subunit 5A, mitochondrial
COX6C_MOUSE	377	8464	44	2	2	Cytochrome c oxidase subunit 6C
COX7C_MOUSE	46	7328	4	1	1	Cytochrome c oxidase subunit 7C, mitochondrial
CP27A_MOUSE	35	60910	1	1	1	Sterol 26-hydroxylase, mitochondrial
CP2A4_MOUSE	23	56859	1	1	1	Cytochrome P450 2A4
CPT1B_MOUSE	3814	89073	219	23	23	Carnitine O-palmitoyltransferase 1, muscle isoform
CPT2_MOUSE	657	74504	23	4	4	Carnitine O-palmitoyltransferase 2, mitochondrial
CQ10B_MOUSE	63	27764	12	1	1	Coenzyme Q-binding protein COQ10 homolog B, mitochondrial
CRYAB_MOUSE	440	20056	26	7	7	Alpha-crystallin B chain
CSRP3_MOUSE	39	21793	1	1	1	Cysteine and glycine-rich protein 3
CTBL1_MOUSE	30	65395	1	1	1	Beta-catenin-like protein 1
CTL2_MOUSE	59	81539	2	1	1	Choline transporter-like protein 2
CTR9_MOUSE	21	134181	1	1	1	RNA polymerase-associated protein CTR9 homolog
CX6B1_MOUSE	538	10293	28	4	4	Cytochrome c oxidase subunit 6B1
CX7A1_MOUSE	365	9095	24	2	2	Cytochrome c oxidase subunit 7A1, mitochondrial
CX7A2_MOUSE	102	9285	3	1	1	Cytochrome c oxidase subunit 7A2, mitochondrial
CY1_MOUSE	3270	35533	141	8	8	Cytochrome c1, heme protein, mitochondrial
CYB_MOUSE	408	43352	20	1	1	Cytochrome b
CYB5B_MOUSE	30	16365	1	1	1	Cytochrome b5 type B
CYC_MOUSE	361	11712	34	2	2	Cytochrome c, somatic
D39U1_MOUSE	42	33145	2	2	2	Epimerase family protein SDR39U1
DAD1_MOUSE	25	12660	1	1	1	Dolichyl-diphosphooligosaccharide--protein glycosyltransferase subunit DAD1
DCTN2_MOUSE	40	44204	1	1	1	Dynactin subunit 2
DDB1_MOUSE	31	128027	1	1	1	DNA damage-binding protein 1
DDX17_MOUSE	28	72981	6	1	1	Probable ATP-dependent RNA helicase DDX17
DDX3L_MOUSE	53	73494	1	1	1	Putative ATP-dependent RNA helicase Pl10
DECR_MOUSE	729	36476	44	7	7	2,4-dienoyl-CoA reductase, mitochondrial
DERM_MOUSE	587	24549	30	3	3	Dermatopontin
DESM_MOUSE	20840	53522	749	33	28	Desmin
DHRS3_MOUSE	27	34030	1	1	1	Short-chain dehydrogenase/reductase 3
DHSD_MOUSE	29	17288	1	1	1	Succinate dehydrogenase [ubiquinone] cytochrome b small subunit, mitochondrial

DICER_MOUSE	136	219420	11	1	1	Endoribonuclease Dicer
DIEXF_MOUSE	24	89162	1	1	1	Digestive organ expansion factor homolog
DJB11_MOUSE	38	40815	1	1	1	DnaJ homolog subfamily B member 11
DJC11_MOUSE	55	63536	3	2	2	DnaJ homolog subfamily C member 11
DKC1_MOUSE	56	57821	2	1	1	H/ACA ribonucleoprotein complex subunit 4
DLDH_MOUSE	1966	54751	102	8	8	Dihydrolipoyl dehydrogenase, mitochondrial
DMD_MOUSE	1867	427676	79	13	13	Dystrophin
DMPK_MOUSE	20	70241	1	1	1	Myotonin-protein kinase
DNJA2_MOUSE	33	46344	2	1	1	DnaJ homolog subfamily A member 2
DNJA3_MOUSE	66	53095	6	2	2	DnaJ homolog subfamily A member 3, mitochondrial
DNJC8_MOUSE	24	29794	1	1	1	DnaJ homolog subfamily C member 8
DNM1L_MOUSE	55	83120	2	1	1	Dynamin-1-like protein
DNM3B_MOUSE	23	98592	1	1	1	DNA (cytosine-5)-methyltransferase 3B
DPYL2_MOUSE	31	62638	1	1	1	Dihydropyrimidinase-related protein 2
DRS7C_MOUSE	1776	34958	74	10	10	Dehydrogenase/reductase SDR family member 7C
DTNA_MOUSE	240	84812	12	2	2	Dystrobrevin alpha
DUPD1_MOUSE	38	24405	3	1	1	Dual specificity phosphatase DUPD1
DUS3_MOUSE	70	20687	6	1	1	Dual specificity protein phosphatase 3
DX39A_MOUSE	29	49549	2	1	1	ATP-dependent RNA helicase DDX39A
DYHC1_MOUSE	536	534447	28	8	8	Cytoplasmic dynein 1 heavy chain 1
DYL1_MOUSE	266	10530	10	1	1	Dynein light chain 1, cytoplasmic
DYSF_MOUSE	2695	240040	115	21	21	Dysferlin
ECHA_MOUSE	8838	83302	354	30	30	Trifunctional enzyme subunit alpha, mitochondrial
ECHB_MOUSE	5142	51639	230	14	14	Trifunctional enzyme subunit beta, mitochondrial
ECI1_MOUSE	254	32515	15	2	2	Enoyl-CoA delta isomerase 1, mitochondrial
ECM29_MOUSE	50	205398	3	1	1	Proteasome adapter and scaffold protein ECM29
EF1A1_MOUSE	3438	50424	144	10	2	Elongation factor 1-alpha 1
EF1A2_MOUSE	5695	50764	255	14	6	Elongation factor 1-alpha 2
EF1D_MOUSE	43	31388	5	1	1	Elongation factor 1-delta
EF1G_MOUSE	737	50371	61	9	9	Elongation factor 1-gamma
EF2_MOUSE	1886	96222	111	14	14	Elongation factor 2
EFTU_MOUSE	470	49876	21	5	5	Elongation factor Tu, mitochondrial
EH1L1_MOUSE	79	185634	5	1	1	EH domain-binding protein 1-like protein 1
EHD2_MOUSE	1133	61307	49	8	8	EH domain-containing protein 2
ENOA_MOUSE	18427	47453	605	14	8	Alpha-enolase
ENOB_MOUSE	29179	47337	1041	25	18	Beta-enolase
ENOG_MOUSE	13462	47609	448	9	2	Gamma-enolase
ENTP2_MOUSE	165	54912	7	2	2	Ectonucleoside triphosphate diphosphohydrolase 2
ERCC5_MOUSE	35	131603	5	1	1	DNA repair protein complementing XP-G cells homolog
ES1_MOUSE	142	28415	15	3	3	ES1 protein homolog, mitochondrial
ESTD_MOUSE	325	31870	12	2	2	S-formylglutathione hydrolase
ESYT1_MOUSE	160	121707	5	2	2	Extended synaptotagmin-1
ETFA_MOUSE	859	35330	45	8	8	Electron transfer flavoprotein subunit alpha, mitochondrial
ETFB_MOUSE	1319	27834	46	8	8	Electron transfer flavoprotein subunit beta
ETFD_MOUSE	928	68903	69	9	9	Electron transfer flavoprotein-ubiquinone oxidoreductase, mitochondrial
EX3L1_MOUSE	29	81838	1	1	1	Exocyst complex component 3-like protein
EXOC4_MOUSE	40	111273	6	1	1	Exocyst complex component 4
EZRI_MOUSE	29	69478	2	1	1	Ezrin
F162A_MOUSE	100	17884	10	4	4	Protein FAM162A
F170B_MOUSE	21	44366	1	1	1	Protein FAM170B
FABP4_MOUSE	399	14755	21	2	2	Fatty acid-binding protein, adipocyte
FABPH_MOUSE	665	14810	42	4	4	Fatty acid-binding protein, heart
FBN1_MOUSE	22176	332668	1240	92	88	Fibrillin-1
FBN2_MOUSE	592	334300	56	7	3	Fibrillin-2
FBRL_MOUSE	52	34400	2	1	1	rRNA 2'-O-methyltransferase fibrillarin
FETUA_MOUSE	61	38100	4	3	3	Alpha-2-HS-glycoprotein
FGF16_MOUSE	38	23877	7	1	1	Fibroblast growth factor 16
FHL1_MOUSE	3510	33806	202	16	16	Four and a half LIM domains protein 1
FHL3_MOUSE	166	33769	12	5	5	Four and a half LIM domains protein 3
FIBA_MOUSE	52	88117	3	2	2	Fibrinogen alpha chain
FIBB_MOUSE	63	55402	8	4	4	Fibrinogen beta chain
FIBG_MOUSE	328	50044	18	3	3	Fibrinogen gamma chain
FILA2_MOUSE	87	253504	12	2	2	Filaggrin-2
FINC_MOUSE	121	276017	10	6	6	Fibronectin
FITM1_MOUSE	2016	32715	101	7	7	Fat storage-inducing transmembrane protein 1
FLNA_MOUSE	1309	283897	70	7	2	Filamin-A
FLNC_MOUSE	16737	293560	835	74	69	Filamin-C
FMOD_MOUSE	5623	43427	254	9	9	Fibromodulin
FN3K_MOUSE	111	35238	7	1	1	Fructosamine-3-kinase
FRIH_MOUSE	41	21224	2	2	2	Ferritin heavy chain
FRIL1_MOUSE	93	20847	3	1	1	Ferritin light chain 1
FSD2_MOUSE	34	79985	1	1	1	Fibronectin type III and SPRY domain-containing protein 2
FUMH_MOUSE	901	54550	53	6	6	Fumarate hydratase, mitochondrial
FXR1_MOUSE	533	76517	20	2	2	Fragile X mental retardation syndrome-related protein 1
G3P_MOUSE	28492	36072	1119	19	19	Glyceraldehyde-3-phosphate dehydrogenase
G45IP_MOUSE	24	25861	2	1	1	Growth arrest and DNA damage-inducible proteins-interacting protein 1
G6PI_MOUSE	2068	62955	143	16	16	Glucose-6-phosphate isomerase
GA2L1_MOUSE	36	72937	5	1	1	GAS2-like protein 1
GABP2_MOUSE	38	45630	5	1	1	GA-binding protein subunit beta-2
GBB1_MOUSE	134	38151	9	2	2	Guanine nucleotide-binding protein G(I)/G(S)/G(T) subunit beta-1
GCAA_MOUSE	43	36936	2	2	2	Ig gamma-2A chain C region, A allele
GDIB_MOUSE	281	51018	10	2	2	Rab GDP dissociation inhibitor beta
GDIR1_MOUSE	58	23450	4	1	1	Rho GDP-dissociation inhibitor 1

GELS_MOUSE	24	86287	3	3	3	Gelsolin
GEPH_MOUSE	25	83971	1	1	1	Gephyrin
GFRA4_MOUSE	25	29283	1	1	1	GDNF family receptor alpha-4
GLYG_MOUSE	412	37606	17	4	4	Glycogenin-1
GNAL_MOUSE	57	44794	2	1	1	Guanine nucleotide-binding protein G(olf) subunit alpha
GNPI2_MOUSE	20	31292	1	1	1	Glucosamine-6-phosphate isomerase 2
GOT1B_MOUSE	78	15411	3	1	1	Vesicle transport protein GOT1B
GPDA_MOUSE	1459	38176	72	9	9	Glycerol-3-phosphate dehydrogenase [NAD(+)], cytoplasmic
GPDM_MOUSE	874	81416	48	12	12	Glycerol-3-phosphate dehydrogenase, mitochondrial
GPX4_MOUSE	79	22925	6	1	1	Phospholipid hydroperoxide glutathione peroxidase
GRIK1_MOUSE	35	95768	1	1	1	Glutamate receptor ionotropic, kainate 1
GRP75_MOUSE	510	73701	37	8	8	Stress-70 protein, mitochondrial
GSHB_MOUSE	56	52442	3	1	1	Glutathione synthetase
GSTM1_MOUSE	661	26067	26	3	1	Glutathione S-transferase Mu 1
GSTM2_MOUSE	496	25871	19	3	1	Glutathione S-transferase Mu 2
GSTM5_MOUSE	196	27016	7	2	1	Glutathione S-transferase Mu 5
GSTP1_MOUSE	94	23765	5	3	3	Glutathione S-transferase P 1
GTR4_MOUSE	97	54948	6	2	2	Solute carrier family 2, facilitated glucose transporter member 4
GYS1_MOUSE	2534	84672	111	9	9	Glycogen [starch] synthase, muscle
H10_MOUSE	1968	20848	102	5	5	Histone H1.0
H12_MOUSE	1987	21254	135	11	5	Histone H1.2
H14_MOUSE	2198	21964	121	8	2	Histone H1.4
H2A2B_MOUSE	2427	14005	98	5	2	Histone H2A type 2-B
H2AJ_MOUSE	1816	14037	107	6	2	Histone H2A.J
H2AV_MOUSE	968	13501	66	4	2	Histone H2A.V
H2AY_MOUSE	533	39882	27	4	4	Core histone macro-H2A.1
H2B1B_MOUSE	4478	13944	158	8	8	Histone H2B type 1-B
H31_MOUSE	1270	15509	81	6	1	Histone H3.1
H32_MOUSE	2378	15436	104	6	1	Histone H3.2
H3C_MOUSE	3039	15363	118	8	3	Histone H3.3C
H4_MOUSE	3661	11360	184	8	8	Histone H4
HBA_MOUSE	867	15133	63	7	7	Hemoglobin subunit alpha
HBB1_MOUSE	3986	15944	187	11	5	Hemoglobin subunit beta-1
HBB2_MOUSE	1966	15982	128	9	3	Hemoglobin subunit beta-2
HCD2_MOUSE	213	27516	14	4	4	3-hydroxyacyl-CoA dehydrogenase type-2
HCDH_MOUSE	631	34613	28	3	3	Hydroxyacyl-coenzyme A dehydrogenase, mitochondrial
HEMO_MOUSE	137	52026	10	5	5	Hemopexin
HHATL_MOUSE	4867	57021	252	14	14	Protein-cysteine N-palmitoyltransferase HHAT-like protein
HIP1_MOUSE	31	116100	3	1	1	Huntingtin-interacting protein 1
HMGB1_MOUSE	26	25049	1	1	1	High mobility group protein B1
HNRPK_MOUSE	226	51230	16	4	4	Heterogeneous nuclear ribonucleoprotein K
HNRPQ_MOUSE	25	69818	1	1	1	Heterogeneous nuclear ribonucleoprotein Q
HNRPU_MOUSE	241	88661	15	3	3	Heterogeneous nuclear ribonucleoprotein U
HP1B3_MOUSE	93	61057	3	2	2	Heterochromatin protein 1-binding protein 3
HS90B_MOUSE	2406	83571	156	18	18	Heat shock protein HSP 90-beta
HSP7C_MOUSE	6217	71055	307	23	21	Heat shock cognate 71 kDa protein
HSPB1_MOUSE	450	23057	33	7	7	Heat shock protein beta-1
HSPB6_MOUSE	179	17567	15	3	3	Heat shock protein beta-6
HSPB7_MOUSE	188	18680	7	2	2	Heat shock protein beta-7
HSPB8_MOUSE	33	21691	1	1	1	Heat shock protein beta-8
HXK2_MOUSE	784	103951	47	10	10	Hexokinase-2
HYDIN_MOUSE	31	586685	2	1	1	Hydrocephalus-inducing protein
IDH3A_MOUSE	2632	40069	139	12	12	Isocitrate dehydrogenase [NAD] subunit alpha, mitochondrial
IDHG1_MOUSE	983	43157	76	9	9	Isocitrate dehydrogenase [NAD] subunit gamma 1, mitochondrial
IDHP_MOUSE	1505	51330	112	14	14	Isocitrate dehydrogenase [NADP], mitochondrial
IF4A2_MOUSE	77	46601	5	4	4	Eukaryotic initiation factor 4A-II
IF5A1_MOUSE	276	17049	17	3	3	Eukaryotic translation initiation factor 5A-1
IFIH1_MOUSE	21	117209	1	1	1	Interferon-induced helicase C domain-containing protein 1
IGFN1_MOUSE	27	306493	1	1	1	Immunoglobulin-like and fibronectin type III domain-containing protein 1
IGH1M_MOUSE	44	44043	3	1	1	Ig gamma-1 chain C region, membrane-bound form
IGKC_MOUSE	36	12098	2	1	1	Immunoglobulin kappa constant
IMB1_MOUSE	199	98377	9	1	1	Importin subunit beta-1
IQUB_MOUSE	25	92756	1	1	1	IQ and ubiquitin-like domain-containing protein
IRGM1_MOUSE	49	47092	5	2	2	Immunity-related GTPase family M protein 1
ITIH1_MOUSE	51	101460	3	2	2	Inter-alpha-trypsin inhibitor heavy chain H1
ITIH2_MOUSE	34	106261	1	1	1	Inter-alpha-trypsin inhibitor heavy chain H2
ITIH3_MOUSE	85	99638	3	3	3	Inter-alpha-trypsin inhibitor heavy chain H3
ITIH5_MOUSE	44	107139	2	1	1	Inter-alpha-trypsin inhibitor heavy chain H5
IVD_MOUSE	39	46695	2	1	1	Isovaleryl-CoA dehydrogenase, mitochondrial
JAM3_MOUSE	45	35386	7	1	1	Junctional adhesion molecule C
JPH1_MOUSE	249	72374	23	8	8	Junctophilin-1
JPH2_MOUSE	812	74818	54	9	9	Junctophilin-2
JSPR1_MOUSE	1731	36242	80	8	8	Junctional sarcoplasmic reticulum protein 1
K1C10_MOUSE	279	57906	14	6	2	Keratin, type I cytoskeletal 10
K1C15_MOUSE	148	49278	12	5	1	Keratin, type I cytoskeletal 15
K1C17_MOUSE	179	48417	10	4	1	Keratin, type I cytoskeletal 17
K1C19_MOUSE	132	44515	11	4	1	Keratin, type I cytoskeletal 19
K22O_MOUSE	140	63319	8	3	1	Keratin, type II cytoskeletal 2 oral
K2C4_MOUSE	135	56590	8	3	1	Keratin, type II cytoskeletal 4
KAD1_MOUSE	5295	21640	214	12	12	Adenylate kinase isoenzyme 1
KAT2B_MOUSE	53	92680	4	1	1	Histone acetyltransferase KAT2B
KCC2A_MOUSE	2386	54651	117	6	5	Calcium/calmodulin-dependent protein kinase type II subunit alpha
KCC2G_MOUSE	248	60254	19	7	6	Calcium/calmodulin-dependent protein kinase type II subunit gamma

KCJ11_MOUSE	95	43933	2	1	1	ATP-sensitive inward rectifier potassium channel 11
KCNA5_MOUSE	20	67222	1	1	1	Potassium voltage-gated channel subfamily A member 5
KCNV2_MOUSE	20	65332	1	1	1	Potassium voltage-gated channel subfamily V member 2
KCRM_MOUSE	55087	43246	2033	29	10	Creatine kinase M-type
KCRS_MOUSE	15307	47899	641	21	21	Creatine kinase S-type, mitochondrial
KERA_MOUSE	459	40777	29	6	6	Keratocan
KIF14_MOUSE	20	188439	1	1	1	Kinesin-like protein KIF14
KIF15_MOUSE	23	161104	2	1	1	Kinesin-like protein KIF15
KIF19_MOUSE	39	112061	2	1	1	Kinesin-like protein KIF19
KINH_MOUSE	23	110225	1	1	1	Kinesin-1 heavy chain
KLH40_MOUSE	2845	70400	118	11	11	Kelch-like protein 40
KLH41_MOUSE	3697	68945	176	16	16	Kelch-like protein 41
KNG1_MOUSE	32	74140	1	1	1	Kininogen-1
KPB1_MOUSE	1350	139877	84	11	11	Phosphorylase b kinase regulatory subunit alpha, skeletal muscle isoform
KPBB_MOUSE	1408	124951	56	11	11	Phosphorylase b kinase regulatory subunit beta
KPYM_MOUSE	17956	58378	725	29	29	Pyruvate kinase PKM
KR192_MOUSE	21	14128	1	1	1	Keratin-associated protein 19-2
KRT34_MOUSE	2968	46185	131	13	2	Keratin, type I cuticular Ha4
KRT81_MOUSE	2231	54653	122	21	4	Keratin, type II cuticular Hb1
KRT84_MOUSE	35	65798	2	2	1	Keratin, type II cuticular Hb4
KRT85_MOUSE	1450	57377	86	13	1	Keratin, type II cuticular Hb5
KRT87_MOUSE	2048	56190	115	19	2	Keratin, type II cuticular 87
KT33A_MOUSE	3100	47704	132	13	3	Keratin, type I cuticular Ha3-I
KT33B_MOUSE	2319	47488	98	9	1	Keratin, type I cuticular Ha3-II
LAMA2_MOUSE	3190	352831	200	39	39	Laminin subunit alpha-2
LAMA3_MOUSE	26	375058	1	1	1	Laminin subunit alpha-3
LAMA4_MOUSE	26	204372	1	1	1	Laminin subunit alpha-4
LAMA5_MOUSE	510	416165	20	4	4	Laminin subunit alpha-5
LAMB1_MOUSE	917	204203	36	9	8	Laminin subunit beta-1
LAMB2_MOUSE	3558	203579	225	31	30	Laminin subunit beta-2
LAMC1_MOUSE	2324	182830	132	25	25	Laminin subunit gamma-1
LAP2A_MOUSE	63	75807	4	2	2	Lamina-associated polypeptide 2, isoforms alpha/zeta
LDB3_MOUSE	7318	77581	388	19	19	LIM domain-binding protein 3
LDHA_MOUSE	7684	36817	350	17	16	L-lactate dehydrogenase A chain
LDHB_MOUSE	1421	36834	50	5	4	L-lactate dehydrogenase B chain
LEG1_MOUSE	220	15198	13	3	3	Galectin-1
LETM1_MOUSE	36	83621	3	1	1	Mitochondrial proton/calcium exchanger protein
LGUL_MOUSE	119	20967	11	2	2	Lactoylglutathione lyase
LMCD1_MOUSE	21	42167	1	1	1	LIM and cysteine-rich domains protein 1
LMNA_MOUSE	3421	74478	182	27	26	Prelamin-A/C
LMNB1_MOUSE	117	66973	9	3	2	Lamin-B1
LNP_MOUSE	66	47755	2	2	2	Endoplasmic reticulum junction formation protein lunapark
LPPRC_MOUSE	21	157770	1	1	1	Leucine-rich PPR motif-containing protein, mitochondrial
LRC59_MOUSE	28	35312	1	1	1	Leucine-rich repeat-containing protein 59
LRP4_MOUSE	36	217121	2	1	1	Low-density lipoprotein receptor-related protein 4
LUM_MOUSE	498	38640	37	8	8	Lumican
LYRIC_MOUSE	109	63922	6	2	2	Protein LYRIC
M2OM_MOUSE	2977	34304	162	13	13	Mitochondrial 2-oxoglutarate/malate carrier protein
MA2A2_MOUSE	47	131251	4	3	3	Alpha-mannosidase 2x
MACD1_MOUSE	1329	35843	35	5	5	O-acetyl-ADP-ribose deacetylase MACROD1
MAON_MOUSE	167	67683	9	2	2	NADP-dependent malic enzyme, mitochondrial
MAP4_MOUSE	147	117927	9	4	4	Microtubule-associated protein 4
MAVS_MOUSE	30	53822	3	3	3	Mitochondrial antiviral-signaling protein
MBOA7_MOUSE	25	54085	1	1	1	Lysophospholipid acyltransferase 7
MBP_MOUSE	1340	27151	125	4	4	Myelin basic protein
MCAT_MOUSE	112	33347	8	2	2	Mitochondrial carnitine/acylcarnitine carrier protein
MCCA_MOUSE	34	79692	2	2	2	Methylcrotonoyl-CoA carboxylase subunit alpha, mitochondrial
MCPT4_MOUSE	477	27528	26	5	5	Mast cell protease 4
MCU_MOUSE	96	39999	6	2	2	Calcium uniporter protein, mitochondrial
MDHC_MOUSE	2526	36659	112	9	9	Malate dehydrogenase, cytoplasmic
MDHM_MOUSE	11219	36045	442	17	17	Malate dehydrogenase, mitochondrial
MECP2_MOUSE	25	52447	1	1	1	Methyl-CpG-binding protein 2
MELPH_MOUSE	21	65811	2	1	1	Melanophilin
MFAP2_MOUSE	159	21363	14	1	1	Microfibrillar-associated protein 2
MFAP4_MOUSE	66	29261	1	1	1	Microfibril-associated glycoprotein 4
MFAP5_MOUSE	133	18982	9	1	1	Microfibrillar-associated protein 5
MGST3_MOUSE	1582	17118	37	3	3	Microsomal glutathione S-transferase 3
MIC13_MOUSE	31	13422	1	1	1	MIC complex subunit MIC13
MIC19_MOUSE	1235	26546	30	3	3	MIC complex subunit Mic19
MIC26_MOUSE	375	22704	21	3	3	MIC complex subunit Mic26
MIC60_MOUSE	1983	84247	109	14	14	MIC complex subunit Mic60
MIF_MOUSE	161	12667	15	1	1	Macrophage migration inhibitory factor
MIME_MOUSE	3712	34333	102	9	9	Mimecan
MLRS_MOUSE	17345	19057	805	18	18	Myosin regulatory light chain 2, skeletal muscle isoform
MLRV_MOUSE	480	18852	49	6	6	Myosin regulatory light chain 2, ventricular/cardiac muscle isoform
MMSA_MOUSE	41	58335	3	1	1	Methylmalonate-semialdehyde dehydrogenase [acylating], mitochondrial
MO2R3_MOUSE	38	34116	3	1	1	Cell surface glycoprotein CD200 receptor 3
MOT4_MOUSE	164	50968	12	2	2	Monocarboxylate transporter 4
MPC1_MOUSE	1130	12617	44	2	2	Mitochondrial pyruvate carrier 1
MPC2_MOUSE	363	14334	24	4	4	Mitochondrial pyruvate carrier 2
MPCP_MOUSE	4251	40063	226	12	12	Phosphate carrier protein, mitochondrial
MPPA_MOUSE	685	58755	15	1	1	Mitochondrial-processing peptidase subunit alpha
MRO2A_MOUSE	27	192112	2	1	1	Maestro heat-like repeat-containing protein family member 2A

MTCH2_MOUSE	929	34104	61	7	7	Mitochondrial carrier homolog 2
MTMR6_MOUSE	20	71914	1	1	1	Myotubularin-related protein 6
MTMRB_MOUSE	33	79145	1	1	1	Myotubularin-related protein 11
MY18A_MOUSE	182	233637	7	3	3	Unconventional myosin-XVIIIa
MYBPH_MOUSE	3374	53065	154	11	11	Myosin-binding protein H
MYBPP_MOUSE	62	106785	5	1	1	MYCBP-associated protein
MYG_MOUSE	1849	17116	101	7	7	Myoglobin
MYH1_MOUSE	347989	224116	12871	188	40	Myosin-1
MYH11_MOUSE	5185	227743	242	20	15	Myosin-11
MYH3_MOUSE	100593	224736	4469	72	7	Myosin-3
MYH4_MOUSE	638227	223632	20760	205	56	Myosin-4
MYH7_MOUSE	123374	223539	4951	135	84	Myosin-7
MYH7B_MOUSE	17267	222501	786	19	1	Myosin-7B
MYH8_MOUSE	294055	223653	10718	140	14	Myosin-8
MYH9_MOUSE	3685	227429	176	11	7	Myosin-9
MYL1_MOUSE	23014	20695	923	18	14	Myosin light chain 1/3, skeletal muscle isoform
MYL3_MOUSE	9234	22521	436	12	8	Myosin light chain 3
MYL4_MOUSE	1202	21260	46	3	1	Myosin light chain 4
MYLK2_MOUSE	295	66633	29	4	4	Myosin light chain kinase 2, skeletal/cardiac muscle
MYO1C_MOUSE	300	122723	14	3	2	Unconventional myosin-Ic
MYOC_MOUSE	36	55622	2	2	2	Myocilin
MYOM1_MOUSE	26858	186546	1353	83	83	Myomesin-1
MYOM3_MOUSE	2315	162761	100	23	23	Myomesin-3
MYOTI_MOUSE	3092	55738	214	17	17	Myotilin
MYOZ1_MOUSE	10609	31438	457	13	13	Myozenin-1
MYOZ2_MOUSE	407	29800	23	4	4	Myozenin-2
MYOZ3_MOUSE	1371	27022	70	6	6	Myozenin-3
MYP0_MOUSE	7481	27775	361	9	9	Myelin protein P0
MYPC2_MOUSE	55850	128128	2336	70	70	Myosin-binding protein C, fast-type
MYPN_MOUSE	47	145222	5	3	3	Myopalladin
NACAM_MOUSE	266	221277	21	9	9	Nascent polypeptide-associated complex subunit alpha, muscle-specific form
NAR1_MOUSE	49	36331	2	1	1	GPI-linked NAD(P)(+)--arginine ADP-ribosyltransferase 1
NB5R1_MOUSE	102	34284	7	3	3	NADH-cytochrome b5 reductase 1
NB5R3_MOUSE	103	34334	7	4	4	NADH-cytochrome b5 reductase 3
NCAM1_MOUSE	22	120152	1	1	1	Neural cell adhesion molecule 1
NCLN_MOUSE	82	63097	6	2	2	Nicalin
NDE1_MOUSE	31	38556	3	1	1	Nuclear distribution protein nudE homolog 1
NDKB_MOUSE	768	17466	56	4	4	Nucleoside diphosphate kinase B
NDRG2_MOUSE	1338	41105	50	5	5	Protein NDRG2
NDUA1_MOUSE	48	8190	1	1	1	NADH dehydrogenase [ubiquinone] 1 alpha subcomplex subunit 1
NDUA2_MOUSE	49	11023	2	1	1	NADH dehydrogenase [ubiquinone] 1 alpha subcomplex subunit 2
NDUA3_MOUSE	45	9325	2	1	1	NADH dehydrogenase [ubiquinone] 1 alpha subcomplex subunit 3
NDUA4_MOUSE	1033	9321	55	5	5	Cytochrome c oxidase subunit NDUFA4
NDUA5_MOUSE	21	13408	1	1	1	NADH dehydrogenase [ubiquinone] 1 alpha subcomplex subunit 5
NDUA6_MOUSE	60	15273	9	3	3	NADH dehydrogenase [ubiquinone] 1 alpha subcomplex subunit 6
NDUA7_MOUSE	42	12625	4	1	1	NADH dehydrogenase [ubiquinone] 1 alpha subcomplex subunit 7
NDUA8_MOUSE	103	20435	9	2	2	NADH dehydrogenase [ubiquinone] 1 alpha subcomplex subunit 8
NDUA9_MOUSE	5884	42612	222	16	16	NADH dehydrogenase [ubiquinone] 1 alpha subcomplex subunit 9, mitochondrial
NDUAA_MOUSE	2873	40863	121	10	10	NADH dehydrogenase [ubiquinone] 1 alpha subcomplex subunit 10, mitochondrial
NDUAC_MOUSE	399	17133	30	3	3	NADH dehydrogenase [ubiquinone] 1 alpha subcomplex subunit 12
NDUAD_MOUSE	801	16849	39	5	5	NADH dehydrogenase [ubiquinone] 1 alpha subcomplex subunit 13
NDUB2_MOUSE	58	12017	4	1	1	NADH dehydrogenase [ubiquinone] 1 beta subcomplex subunit 2, mitochondrial
NDUB4_MOUSE	89	15072	5	2	2	NADH dehydrogenase [ubiquinone] 1 beta subcomplex subunit 4
NDUB5_MOUSE	455	21753	29	5	5	NADH dehydrogenase [ubiquinone] 1 beta subcomplex subunit 5, mitochondrial
NDUB6_MOUSE	199	15505	23	3	3	NADH dehydrogenase [ubiquinone] 1 beta subcomplex subunit 6
NDUB7_MOUSE	78	16548	6	1	1	NADH dehydrogenase [ubiquinone] 1 beta subcomplex subunit 7
NDUB8_MOUSE	401	21919	26	2	2	NADH dehydrogenase [ubiquinone] 1 beta subcomplex subunit 8, mitochondrial
NDUB9_MOUSE	53	22255	8	3	3	NADH dehydrogenase [ubiquinone] 1 beta subcomplex subunit 9
NDUBA_MOUSE	75	21296	7	2	2	NADH dehydrogenase [ubiquinone] 1 beta subcomplex subunit 10
NDUBB_MOUSE	197	17490	17	1	1	NADH dehydrogenase [ubiquinone] 1 beta subcomplex subunit 11, mitochondrial
NDUC2_MOUSE	984	14211	59	3	3	NADH dehydrogenase [ubiquinone] 1 subunit C2
NDUS1_MOUSE	4164	80752	229	21	21	NADH-ubiquinone oxidoreductase 75 kDa subunit, mitochondrial
NDUS2_MOUSE	581	52991	39	10	10	NADH dehydrogenase [ubiquinone] iron-sulfur protein 2, mitochondrial
NDUS3_MOUSE	5399	30302	158	8	8	NADH dehydrogenase [ubiquinone] iron-sulfur protein 3, mitochondrial
NDUS4_MOUSE	57	19829	5	1	1	NADH dehydrogenase [ubiquinone] iron-sulfur protein 4, mitochondrial
NDUS5_MOUSE	54	12867	1	1	1	NADH dehydrogenase [ubiquinone] iron-sulfur protein 5
NDUS7_MOUSE	1233	24952	49	3	3	NADH dehydrogenase [ubiquinone] iron-sulfur protein 7, mitochondrial
NDUS8_MOUSE	863	24479	30	3	3	NADH dehydrogenase [ubiquinone] iron-sulfur protein 8, mitochondrial
NDUV1_MOUSE	1503	51486	97	14	14	NADH dehydrogenase [ubiquinone] flavoprotein 1, mitochondrial
NDUV2_MOUSE	291	27610	28	3	3	NADH dehydrogenase [ubiquinone] flavoprotein 2, mitochondrial
NED4L_MOUSE	50	115746	4	1	1	E3 ubiquitin-protein ligase NEDD4-like
NEK8_MOUSE	23	76414	1	1	1	Serine/threonine-protein kinase Nek8
NEMO_MOUSE	21	48455	1	1	1	NF-kappa-B essential modulator
NEPRO_MOUSE	29	64266	1	1	1	Nucleolus and neural progenitor protein
NFH_MOUSE	503	117209	32	6	5	Neurofilament heavy polypeptide
NFL_MOUSE	963	61528	84	12	10	Neurofilament light polypeptide
NFM_MOUSE	892	95973	54	9	7	Neurofilament medium polypeptide
NID1_MOUSE	2736	139302	140	17	16	Nidogen-1
NID2_MOUSE	172	156610	16	6	5	Nidogen-2
NIPS2_MOUSE	319	33083	23	4	4	Protein NipSnap homolog 2
NLRP6_MOUSE	206	98766	23	1	1	NACHT, LRR and PYD domains-containing protein 6
NNTM_MOUSE	69	114449	6	4	4	NAD(P) transhydrogenase, mitochondrial
NOG2_MOUSE	132	83579	21	1	1	Nucleolar GTP-binding protein 2

NOL3_MOUSE	36	24781	1	1	1	Nucleolar protein 3
NOL7_MOUSE	106	29009	6	1	1	Nucleolar protein 7
NONO_MOUSE	40	54620	1	1	1	Non-POU domain-containing octamer-binding protein
NOP56_MOUSE	33	64880	1	1	1	Nucleolar protein 56
NPM_MOUSE	31	32711	1	1	1	Nucleophosmin
NU1M_MOUSE	44	36092	2	1	1	NADH-ubiquinone oxidoreductase chain 1
NU4M_MOUSE	304	52018	19	2	2	NADH-ubiquinone oxidoreductase chain 4
NU5M_MOUSE	533	68771	18	2	2	NADH-ubiquinone oxidoreductase chain 5
NUCL_MOUSE	38	76734	3	2	2	Nucleolin
NUDT6_MOUSE	26	35517	1	1	1	Nucleoside diphosphate-linked moiety X motif 6
OAT_MOUSE	30	48723	1	1	1	Ornithine aminotransferase, mitochondrial
OBSCN_MOUSE	5749	979281	314	63	63	Obscurin
OCAD1_MOUSE	24	27821	1	1	1	OCIA domain-containing protein 1
ODO1_MOUSE	7125	117572	323	29	29	2-oxoglutarate dehydrogenase, mitochondrial
ODO2_MOUSE	1789	49306	86	4	4	Dihydrolipoyllysine-residue succinyltransferase component of 2-oxoglutarate dehydrogenase complex, mitochondrial
ODP2_MOUSE	2574	68469	144	11	11	Dihydrolipoyllysine-residue acetyltransferase component of pyruvate dehydrogenase complex, mitochondrial
ODPA_MOUSE	6060	43888	227	13	13	Pyruvate dehydrogenase E1 component subunit alpha, somatic form, mitochondrial
ODPB_MOUSE	3549	39254	191	12	12	Pyruvate dehydrogenase E1 component subunit beta, mitochondrial
ODPX_MOUSE	379	54250	16	1	1	Pyruvate dehydrogenase protein X component, mitochondrial
OLA1_MOUSE	72	44929	3	1	1	Obg-like ATPase 1
OST48_MOUSE	126	49225	5	1	1	Dolichyl-diphosphooligosaccharide--protein glycosyltransferase 48 kDa subunit
PACN3_MOUSE	192	48897	17	7	7	Protein kinase C and casein kinase II substrate protein 3
PADI2_MOUSE	190	76942	11	2	2	Protein-arginine deiminase type-2
PAIRB_MOUSE	77	44801	6	3	3	Plasminogen activator inhibitor 1 RNA-binding protein
PARK7_MOUSE	233	20236	18	3	3	Protein/nucleic acid deglycase DJ-1
PARVB_MOUSE	25	41700	1	1	1	Beta-parvin
PBIP1_MOUSE	1150	81514	42	9	9	Pre-B-cell leukemia transcription factor-interacting protein 1
PDIA1_MOUSE	700	57422	40	5	5	Protein disulfide-isomerase
PDK2_MOUSE	209	46239	12	1	1	[Pyruvate dehydrogenase (acetyl-transferring)] kinase isozyme 2, mitochondrial
PDLI3_MOUSE	1749	34734	66	9	9	PDZ and LIM domain protein 3
PDLI5_MOUSE	2842	64514	149	8	8	PDZ and LIM domain protein 5
PDLI7_MOUSE	1413	51170	75	6	6	PDZ and LIM domain protein 7
PDPR_MOUSE	39	100079	1	1	1	Pyruvate dehydrogenase phosphatase regulatory subunit, mitochondrial
PEBP1_MOUSE	144	20988	8	3	3	Phosphatidylethanolamine-binding protein 1
PEDF_MOUSE	129	46262	4	1	1	Pigment epithelium-derived factor
PFKAM_MOUSE	28375	86070	1119	40	40	ATP-dependent 6-phosphofructokinase, muscle type
PGAM2_MOUSE	7873	28980	348	15	15	Phosphoglycerate mutase 2
PGBM_MOUSE	17861	407847	896	88	88	Basement membrane-specific heparan sulfate proteoglycan core protein
PGK1_MOUSE	4380	44921	191	15	15	Phosphoglycerate kinase 1
PGM1_MOUSE	6584	61665	381	27	27	Phosphoglucomutase-1
PGM5_MOUSE	268	62751	34	3	3	Phosphoglucomutase-like protein 5
PGS1_MOUSE	2400	42069	122	12	11	Biglycan
PGS2_MOUSE	5064	40126	295	14	13	Decorin
PHB_MOUSE	403	29859	21	8	8	Prohibitin
PHB2_MOUSE	827	33276	39	8	8	Prohibitin-2
PHF8_MOUSE	22	114508	1	1	1	Histone lysine demethylase PHF8
PHKG1_MOUSE	509	45387	48	5	5	Phosphorylase b kinase gamma catalytic chain, skeletal muscle/heart isoform
PHLB2_MOUSE	35	142253	1	1	1	Pleckstrin homology-like domain family B member 2
PIEZ1_MOUSE	27	295063	1	1	1	Piezo-type mechanosensitive ion channel component 1
PKHS1_MOUSE	27	53728	1	1	1	Pleckstrin homology domain-containing family S member 1
PLCA_MOUSE	99	32031	3	1	1	1-acyl-sn-glycerol-3-phosphate acyltransferase alpha
PLCC_MOUSE	36	43952	3	2	2	1-acyl-sn-glycerol-3-phosphate acyltransferase gamma
PLCD4_MOUSE	21	93547	1	1	1	1-phosphatidylinositol 4,5-bisphosphate phosphodiesterase delta-4
PLD1_MOUSE	31	124689	1	1	1	Phospholipase D1
PLEC_MOUSE	26938	535800	1417	167	167	Plectin
PLM_MOUSE	437	10544	25	2	2	Phospholemman
PMFBP_MOUSE	29	120467	1	1	1	Polyamine-modulated factor 1-binding protein 1
POSTN_MOUSE	344	93769	10	1	1	Periostin
PP1B_MOUSE	220	37961	14	2	2	Serine/threonine-protein phosphatase PP1-beta catalytic subunit
PP2BA_MOUSE	31	59291	3	1	1	Serine/threonine-protein phosphatase 2B catalytic subunit alpha isoform
PPAC_MOUSE	27	18636	1	1	1	Low molecular weight phosphotyrosine protein phosphatase
PPCE_MOUSE	20	81669	1	1	1	Prolyl endopeptidase
PPIA_MOUSE	76	18131	6	2	2	Peptidyl-prolyl cis-trans isomerase A
PPR3A_MOUSE	25	122443	2	2	2	Protein phosphatase 1 regulatory subunit 3A
PRAF3_MOUSE	529	21543	30	2	2	PRA1 family protein 3
PRAX_MOUSE	774	147823	60	16	16	Periaxin
PRDX1_MOUSE	746	22390	66	5	5	Peroxiredoxin-1
PRDX2_MOUSE	179	21936	16	3	3	Peroxiredoxin-2
PRDX3_MOUSE	146	28337	7	3	3	Thioredoxin-dependent peroxide reductase, mitochondrial
PRDX5_MOUSE	27	22226	1	1	1	Peroxiredoxin-5, mitochondrial
PRDX6_MOUSE	63	24969	3	1	1	Peroxiredoxin-6
PREB_MOUSE	25	45979	2	1	1	Prolactin regulatory element-binding protein
PRELP_MOUSE	3583	43607	155	11	11	Prolargin
PRG2_MOUSE	45	24981	1	1	1	Bone marrow proteoglycan
PROX1_MOUSE	32	83701	1	1	1	Prospero homeobox protein 1
PRP8_MOUSE	20	274754	1	1	1	Pre-mRNA-processing-splicing factor 8
PRPS1_MOUSE	2497	35325	94	10	10	Ribose-phosphate pyrophosphokinase 1
PRR33_MOUSE	431	28673	19	1	1	Proline-rich protein 33
PRS38_MOUSE	20	36245	1	1	1	Serine protease 38
PRS6A_MOUSE	22	49803	1	1	1	26S proteasome regulatory subunit 6A
PRS8_MOUSE	59	45768	4	1	1	26S proteasome regulatory subunit 8
PRVA_MOUSE	1937	11923	79	5	5	Parvalbumin alpha
PSA1_MOUSE	57	29813	8	1	1	Proteasome subunit alpha type-1

PSB2_MOUSE	51	23063	3	1	1	Proteasome subunit beta type-2
PSB6_MOUSE	31	25591	1	1	1	Proteasome subunit beta type-6
PSD1_MOUSE	42	110360	1	1	1	PH and SEC7 domain-containing protein 1
PSD13_MOUSE	211	43124	9	1	1	26S proteasome non-ATPase regulatory subunit 13
PSMD2_MOUSE	124	100937	9	2	2	26S proteasome non-ATPase regulatory subunit 2
PSMD3_MOUSE	67	60794	2	1	1	26S proteasome non-ATPase regulatory subunit 3
PSMD7_MOUSE	36	36574	2	1	1	26S proteasome non-ATPase regulatory subunit 7
PSME1_MOUSE	33	28826	1	1	1	Proteasome activator complex subunit 1
PTH2_MOUSE	36	19742	1	1	1	Peptidyl-tRNA hydrolase 2, mitochondrial
PTPRB_MOUSE	43	225838	2	1	1	Receptor-type tyrosine-protein phosphatase beta
PTPRT_MOUSE	22	164677	1	1	1	Receptor-type tyrosine-protein phosphatase T
PUR8_MOUSE	24	55572	1	1	1	Adenylosuccinate lyase
PURA1_MOUSE	2717	50508	122	11	11	Adenylosuccinate synthetase isozyme 1
PXMP2_MOUSE	24	22251	1	1	1	Peroxisomal membrane protein 2
PYC_MOUSE	32	130344	1	1	1	Pyruvate carboxylase, mitochondrial
PYGB_MOUSE	18447	97353	666	15	3	Glycogen phosphorylase, brain form
PYGL_MOUSE	7366	97857	322	7	1	Glycogen phosphorylase, liver form
PYGM_MOUSE	73787	97681	2802	61	48	Glycogen phosphorylase, muscle form
PZP_MOUSE	85	167116	4	1	1	Pregnancy zone protein
QCR1_MOUSE	6987	53446	355	18	18	Cytochrome b-c1 complex subunit 1, mitochondrial
QCR10_MOUSE	22	6534	1	1	1	Cytochrome b-c1 complex subunit 10
QCR2_MOUSE	9282	48262	379	19	19	Cytochrome b-c1 complex subunit 2, mitochondrial
QCR6_MOUSE	151	10713	4	2	2	Cytochrome b-c1 complex subunit 6, mitochondrial
QCR7_MOUSE	39	13519	4	2	2	Cytochrome b-c1 complex subunit 7
QCR8_MOUSE	940	9762	53	4	4	Cytochrome b-c1 complex subunit 8
QCR9_MOUSE	633	7441	33	2	2	Cytochrome b-c1 complex subunit 9
QKI_MOUSE	20	37761	1	1	1	Protein quaking
RAB10_MOUSE	833	22755	40	5	5	Ras-related protein Rab-10
RAC1_MOUSE	70	21835	6	2	2	Ras-related C3 botulinum toxin substrate 1
RACK1_MOUSE	190	35511	12	3	3	Receptor of activated protein C kinase 1
RAN_MOUSE	34	24579	2	1	1	GTP-binding nuclear protein Ran
RAP1A_MOUSE	99	21316	2	1	1	Ras-related protein Rap-1A
RBBP5_MOUSE	50	59745	5	1	1	Retinoblastoma-binding protein 5
RCN1_MOUSE	107	38090	8	1	1	Reticulocalbin-1
RHG31_MOUSE	119	156549	10	1	1	Rho GTPase-activating protein 31
RHOA_MOUSE	28	22110	1	1	1	Transforming protein RhoA
RIBC1_MOUSE	26	45106	1	1	1	RIB43A-like with coiled-coils protein 1
RIN3_MOUSE	42	108122	6	1	1	Ras and Rab interactor 3
RL10A_MOUSE	347	25072	42	6	6	60S ribosomal protein L10a
RL10L_MOUSE	670	24998	41	4	4	60S ribosomal protein L10-like
RL12_MOUSE	29	17965	1	1	1	60S ribosomal protein L12
RL13_MOUSE	1065	24348	76	6	6	60S ribosomal protein L13
RL13A_MOUSE	137	23563	15	4	4	60S ribosomal protein L13a
RL14_MOUSE	1658	23663	68	4	4	60S ribosomal protein L14
RL15_MOUSE	846	24245	58	7	7	60S ribosomal protein L15
RL17_MOUSE	230	21637	16	3	3	60S ribosomal protein L17
RL18_MOUSE	1447	21688	70	5	5	60S ribosomal protein L18
RL18A_MOUSE	786	21004	44	4	4	60S ribosomal protein L18a
RL19_MOUSE	559	23565	28	3	3	60S ribosomal protein L19
RL21_MOUSE	142	18607	12	1	1	60S ribosomal protein L21
RL22_MOUSE	285	14807	15	3	3	60S ribosomal protein L22
RL22L_MOUSE	34	14459	1	1	1	60S ribosomal protein L22-like 1
RL23_MOUSE	359	14970	22	2	2	60S ribosomal protein L23
RL23A_MOUSE	138	17684	5	2	2	60S ribosomal protein L23a
RL24_MOUSE	553	17882	32	5	5	60S ribosomal protein L24
RL26_MOUSE	387	17248	36	4	4	60S ribosomal protein L26
RL27_MOUSE	604	15788	45	4	4	60S ribosomal protein L27
RL27A_MOUSE	308	16709	16	3	3	60S ribosomal protein L27a
RL28_MOUSE	142	15781	13	3	3	60S ribosomal protein L28
RL29_MOUSE	60	17633	5	2	2	60S ribosomal protein L29
RL3_MOUSE	301	46366	23	3	3	60S ribosomal protein L3
RL30_MOUSE	907	12947	36	3	3	60S ribosomal protein L30
RL31_MOUSE	251	14454	18	1	1	60S ribosomal protein L31
RL34_MOUSE	317	13513	28	4	4	60S ribosomal protein L34
RL35_MOUSE	72	14544	8	2	2	60S ribosomal protein L35
RL35A_MOUSE	63	12660	8	2	2	60S ribosomal protein L35a
RL36_MOUSE	171	12265	13	1	1	60S ribosomal protein L36
RL36A_MOUSE	98	12718	14	4	4	60S ribosomal protein L36a
RL39_MOUSE	29	6403	1	1	1	60S ribosomal protein L39
RL4_MOUSE	1304	47409	101	13	13	60S ribosomal protein L4
RL5_MOUSE	653	34607	33	4	4	60S ribosomal protein L5
RL6_MOUSE	3446	33546	105	8	8	60S ribosomal protein L6
RL7_MOUSE	1278	31457	58	9	9	60S ribosomal protein L7
RL7A_MOUSE	1083	30129	63	10	10	60S ribosomal protein L7a
RL8_MOUSE	1213	28235	62	5	5	60S ribosomal protein L8
RL9_MOUSE	443	21982	20	4	4	60S ribosomal protein L9
RLA0_MOUSE	40	34366	3	1	1	60S acidic ribosomal protein P0
RN208_MOUSE	21	29283	2	1	1	RING finger protein 208
ROA2_MOUSE	189	37437	19	2	2	Heterogeneous nuclear ribonucleoproteins A2/B1
ROCK2_MOUSE	21	161625	1	1	1	Rho-associated protein kinase 2
RPGP2_MOUSE	20	78718	1	1	1	Rap1 GTPase-activating protein 2
RPN1_MOUSE	87	68657	6	3	3	Dolichyl-diphosphooligosaccharide--protein glycosyltransferase subunit 1
RPN2_MOUSE	653	69134	22	2	2	Dolichyl-diphosphooligosaccharide--protein glycosyltransferase subunit 2

RS10_MOUSE	386	18904	24	3	3	40S ribosomal protein S10
RS11_MOUSE	341	18590	26	5	5	40S ribosomal protein S11
RS13_MOUSE	436	17212	34	5	5	40S ribosomal protein S13
RS14_MOUSE	603	16434	27	2	2	40S ribosomal protein S14
RS15_MOUSE	619	17029	22	2	2	40S ribosomal protein S15
RS15A_MOUSE	60	14944	3	2	2	40S ribosomal protein S15a
RS16_MOUSE	962	16549	43	7	7	40S ribosomal protein S16
RS18_MOUSE	829	17708	51	9	9	40S ribosomal protein S18
RS19_MOUSE	373	16076	22	4	4	40S ribosomal protein S19
RS2_MOUSE	548	31497	29	4	4	40S ribosomal protein S2
RS20_MOUSE	172	13478	12	2	2	40S ribosomal protein S20
RS23_MOUSE	138	15969	8	2	2	40S ribosomal protein S23
RS24_MOUSE	177	15413	9	1	1	40S ribosomal protein S24
RS25_MOUSE	415	13791	30	3	3	40S ribosomal protein S25
RS26_MOUSE	115	13292	12	2	2	40S ribosomal protein S26
RS27A_MOUSE	831	18282	49	3	3	Ubiquitin-40S ribosomal protein S27a
RS27L_MOUSE	99	9813	8	1	1	40S ribosomal protein S27-like
RS3_MOUSE	144	26828	5	2	2	40S ribosomal protein S3
RS30_MOUSE	129	6644	9	1	1	40S ribosomal protein S30
RS3A_MOUSE	918	30094	46	10	10	40S ribosomal protein S3a
RS4X_MOUSE	841	29807	44	7	7	40S ribosomal protein S4, X isoform
RS5_MOUSE	297	23046	18	3	3	40S ribosomal protein S5
RS6_MOUSE	918	28834	43	6	6	40S ribosomal protein S6
RS7_MOUSE	312	22113	23	4	4	40S ribosomal protein S7
RS8_MOUSE	1358	24475	77	7	7	40S ribosomal protein S8
RS9_MOUSE	504	22635	43	9	9	40S ribosomal protein S9
RSMB_MOUSE	19	23811	1	1	1	Small nuclear ribonucleoprotein-associated protein B
RSSA_MOUSE	1191	32931	59	5	5	40S ribosomal protein SA
RTN2_MOUSE	8656	51543	217	6	6	Reticulon-2
RTN4_MOUSE	1636	127048	102	6	6	Reticulon-4
RYR1_MOUSE	61290	570381	2818	182	182	Ryanodine receptor 1
S2542_MOUSE	79	35276	3	1	1	Mitochondrial coenzyme A transporter SLC25A42
S2551_MOUSE	89	33896	4	1	1	Solute carrier family 25 member 51
S27A1_MOUSE	80	72200	5	2	2	Long-chain fatty acid transport protein 1
S61A2_MOUSE	210	52670	12	1	1	Protein transport protein Sec61 subunit alpha isoform 2
SAC1_MOUSE	24	67471	1	1	1	Phosphatidylinositide phosphatase SAC1
SAC2_MOUSE	33	128782	1	1	1	Phosphatidylinositide phosphatase SAC2
SAM50_MOUSE	134	52230	12	2	2	Sorting and assembly machinery component 50 homolog
SAMH1_MOUSE	28	73517	1	1	1	Deoxynucleoside triphosphate triphosphohydrolase SAMHD1
SAPC2_MOUSE	36	43209	1	1	1	Suppressor APC domain-containing protein 2
SAR1B_MOUSE	122	22482	14	4	4	GTP-binding protein SAR1b
SC22B_MOUSE	31	24896	2	2	2	Vesicle-trafficking protein SEC22b
SCAM2_MOUSE	37	36897	1	1	1	Secretory carrier-associated membrane protein 2
SCOT1_MOUSE	41	56352	2	1	1	Succinyl-CoA:3-ketoacid coenzyme A transferase 1, mitochondrial
SCPDL_MOUSE	84	47669	6	3	3	Saccharopine dehydrogenase-like oxidoreductase
SDHA_MOUSE	4323	73623	202	17	17	Succinate dehydrogenase [ubiquinone] flavoprotein subunit, mitochondrial
SDHB_MOUSE	997	32591	73	8	8	Succinate dehydrogenase [ubiquinone] iron-sulfur subunit, mitochondrial
SFI1_MOUSE	33	145596	1	1	1	Protein SFI1 homolog
SFXN3_MOUSE	65	35669	3	2	2	Sideroflexin-3
SGCA_MOUSE	239	43659	20	5	5	Alpha-sarcoglycan
SGCB_MOUSE	26	35193	1	1	1	Beta-sarcoglycan
SGCG_MOUSE	114	32347	4	1	1	Gamma-sarcoglycan
SH3BG_MOUSE	258	23146	25	3	3	SH3 domain-binding glutamic acid-rich protein
SIR7_MOUSE	29	45745	1	1	1	NAD-dependent protein deacetylase sirtuin-7
SMTL2_MOUSE	70	49778	3	1	1	Smoothelin-like protein 2
SMYD1_MOUSE	3659	57428	127	13	13	Histone-lysine N-methyltransferase Smyd1
SNTA1_MOUSE	690	54088	40	4	4	Alpha-1-syntrophin
SODC_MOUSE	470	16104	21	3	3	Superoxide dismutase [Cu-Zn]
SODM_MOUSE	79	24816	4	2	2	Superoxide dismutase [Mn], mitochondrial
SPA3K_MOUSE	559	47021	35	5	5	Serine protease inhibitor A3K
SPB6_MOUSE	144	42913	7	3	3	Serpin B6
SPCS_MOUSE	86	56032	9	1	1	O-phosphoseryl-tRNA(Sec) selenium transferase
SPEG_MOUSE	457	356975	23	5	5	Striated muscle-specific serine/threonine-protein kinase
SPN90_MOUSE	40	79207	3	1	1	NCK-interacting protein with SH3 domain
SPTB1_MOUSE	184	245897	16	6	6	Spectrin beta chain, erythrocytic
SPTB2_MOUSE	224	274908	14	3	3	Spectrin beta chain, non-erythrocytic 1
SRCA_MOUSE	21248	99522	762	35	35	Sarcalumenin
SRPRB_MOUSE	40	29732	1	1	1	Signal recognition particle receptor subunit beta
SRPX_MOUSE	29	52795	1	1	1	Sushi-repeat-containing protein SRPX
SSPN_MOUSE	52	24640	5	2	2	Sarcospan
SSRD_MOUSE	20	19153	1	1	1	Translocon-associated protein subunit delta
ST17B_MOUSE	41	42469	1	1	1	Serine/threonine-protein kinase 17B
STAC3_MOUSE	59	41317	4	2	2	SH3 and cysteine-rich domain-containing protein 3
STIM1_MOUSE	52	77803	5	2	2	Stromal interaction molecule 1
STX11_MOUSE	20	33747	1	1	1	Syntaxin-11
SUCA_MOUSE	305	36474	34	4	4	Succinate--CoA ligase [ADP/GDP-forming] subunit alpha, mitochondrial
SUCB1_MOUSE	843	50424	47	11	11	Succinate--CoA ligase [ADP-forming] subunit beta, mitochondrial
SUCB2_MOUSE	36	47096	2	1	1	Succinate--CoA ligase [GDP-forming] subunit beta, mitochondrial
SURF4_MOUSE	476	30589	13	3	3	Surfeit locus protein 4
SYAC_MOUSE	21	107697	1	1	1	Alanine--tRNA ligase, cytoplasmic
SYC1L_MOUSE	23	28557	1	1	1	Synaptonemal complex central element protein 1-like
SYCP2_MOUSE	23	173839	1	1	1	Synaptonemal complex protein 2
SYDC_MOUSE	52	57567	3	2	2	Aspartate--tRNA ligase, cytoplasmic

SYEP_MOUSE	105	171739	8	2	2	Bifunctional glutamate/proline--tRNA ligase
SYIM_MOUSE	82	113873	3	1	1	Isoleucine--tRNA ligase, mitochondrial
SYNE2_MOUSE	78	787997	11	1	1	Nesprin-2
SYNEM_MOUSE	1438	173276	95	18	18	Synemin
SYNP2_MOUSE	1185	116968	61	7	7	Synaptopodin-2
SYP2L_MOUSE	841	103175	59	12	12	Synaptopodin 2-like protein
SYPL2_MOUSE	1835	29605	55	3	3	Synaptophysin-like protein 2
SYT16_MOUSE	83	71894	8	1	1	Synaptotagmin-16
SYVC_MOUSE	92	141439	4	1	1	Valine--tRNA ligase
TAGL2_MOUSE	36	22552	1	1	1	Transgelin-2
TBA1B_MOUSE	5053	50804	196	12	3	Tubulin alpha-1B chain
TBA4A_MOUSE	6260	50634	230	14	5	Tubulin alpha-4A chain
TBB2A_MOUSE	2578	50274	92	10	2	Tubulin beta-2A chain
TBB4B_MOUSE	3550	50255	164	15	7	Tubulin beta-4B chain
TCPZ_MOUSE	467	58424	17	1	1	T-complex protein 1 subunit zeta
TCTP_MOUSE	64	19564	8	2	2	Translationally-controlled tumor protein
TDRD9_MOUSE	30	157364	1	1	1	ATP-dependent RNA helicase TDRD9
TECR_MOUSE	1213	36466	51	7	7	Very-long-chain enoyl-CoA reductase
TELT_MOUSE	386	19294	33	7	7	Telethonin
TENA_MOUSE	99	237304	5	3	3	Tenascin
TERA_MOUSE	1693	89950	89	15	15	Transitional endoplasmic reticulum ATPase
TGM2_MOUSE	166	78153	11	4	4	Protein-glutamine gamma-glutamyltransferase 2
TGRM1_MOUSE	29	196100	1	1	1	TOG array regulator of axonemal microtubules protein 1
THIL_MOUSE	397	45129	32	6	6	Acetyl-CoA acetyltransferase, mitochondrial
THIM_MOUSE	985	42260	64	9	9	3-ketoacyl-CoA thiolase, mitochondrial
THS7B_MOUSE	26	186774	1	1	1	Thrombospondin type-1 domain-containing protein 7B
THTM_MOUSE	35	33305	4	2	2	3-mercaptopyruvate sulfurtransferase
TIM50_MOUSE	66	39980	2	1	1	Mitochondrial import inner membrane translocase subunit TIM50
TITIN_MOUSE	669588	3933362	31319	1582	1581	Titin
TLK2_MOUSE	20	83008	1	1	1	Serine/threonine-protein kinase tousled-like 2
TLN1_MOUSE	103	271820	5	2	2	Talin-1
TLN2_MOUSE	46	255743	1	1	1	Talin-2
TM109_MOUSE	242	26346	7	2	2	Transmembrane protein 109
TM38A_MOUSE	2891	33686	121	9	9	Trimeric intracellular cation channel type A
TMCO1_MOUSE	197	21389	8	1	1	Calcium load-activated calcium channel
TMM11_MOUSE	107	21754	12	2	2	Transmembrane protein 11, mitochondrial
TMM43_MOUSE	32	44869	1	1	1	Transmembrane protein 43
TMM65_MOUSE	26	25187	1	1	1	Transmembrane protein 65
TMOD1_MOUSE	530	40555	35	4	3	Tropomodulin-1
TMOD4_MOUSE	1960	39579	101	11	10	Tropomodulin-4
TMX2_MOUSE	136	34320	5	1	1	Thioredoxin-related transmembrane protein 2
TNMD_MOUSE	24	37822	1	1	1	Tenomodulin
TNNC2_MOUSE	7586	18155	293	12	11	Troponin C, skeletal muscle
TNNI1_MOUSE	1869	21856	71	6	6	Troponin I, slow skeletal muscle
TNNI2_MOUSE	8091	21515	359	10	10	Troponin I, fast skeletal muscle
TNNT1_MOUSE	630	31325	29	6	6	Troponin T, slow skeletal muscle
TNNT3_MOUSE	30779	32221	1115	16	16	Troponin T, fast skeletal muscle
TOM70_MOUSE	22	68288	1	1	1	Mitochondrial import receptor subunit TOM70
TPIS_MOUSE	5683	32684	243	13	13	Triosephosphate isomerase
TPM1_MOUSE	19023	32718	852	31	13	Tropomyosin alpha-1 chain
TPM2_MOUSE	14905	32931	667	30	18	Tropomyosin beta chain
TPM3_MOUSE	5660	33031	331	22	8	Tropomyosin alpha-3 chain
TRDN_MOUSE	1159	77967	89	7	7	Triadin
TRFE_MOUSE	1915	78841	93	14	14	Serotransferrin
TRI72_MOUSE	2389	53695	117	13	13	Tripartite motif-containing protein 72
TSKS_MOUSE	62	64150	2	1	1	Testis-specific serine kinase substrate
TSP1_MOUSE	47	133555	1	1	1	Thrombospondin-1
TSP4_MOUSE	1378	108979	94	17	15	Thrombospondin-4
TT21A_MOUSE	21	150232	1	1	1	Tetratricopeptide repeat protein 21A
TUFT1_MOUSE	43	44720	3	1	1	Tuftelin
TXD16_MOUSE	36	92674	2	1	1	Thioredoxin domain-containing protein 16
UB2L3_MOUSE	54	18021	2	1	1	Ubiquitin-conjugating enzyme E2 L3
UB2V1_MOUSE	49	16458	4	2	2	Ubiquitin-conjugating enzyme E2 variant 1
UBA1_MOUSE	116	118931	7	2	2	Ubiquitin-like modifier-activating enzyme 1
UCP3_MOUSE	26	34288	1	1	1	Mitochondrial uncoupling protein 3
UCRI_MOUSE	925	29634	91	9	9	Cytochrome b-c1 complex subunit Rieske, mitochondrial
UGPA_MOUSE	1453	57115	69	8	8	UTP--glucose-1-phosphate uridylyltransferase
USMG5_MOUSE	1008	6434	39	2	2	Up-regulated during skeletal muscle growth protein 5
VAPA_MOUSE	325	28065	13	3	2	Vesicle-associated membrane protein-associated protein A
VAPB_MOUSE	417	27157	20	5	4	Vesicle-associated membrane protein-associated protein B
VAT1_MOUSE	39	43297	1	1	1	Synaptic vesicle membrane protein VAT-1 homolog
VDAC1_MOUSE	11305	32502	413	18	18	Voltage-dependent anion-selective channel protein 1
VDAC2_MOUSE	1708	32340	96	10	10	Voltage-dependent anion-selective channel protein 2
VDAC3_MOUSE	4289	31076	182	10	10	Voltage-dependent anion-selective channel protein 3
VIME_MOUSE	2803	53712	175	24	18	Vimentin
VINC_MOUSE	136	117215	13	3	3	Vinculin
VKORL_MOUSE	42	20051	2	1	1	Vitamin K epoxide reductase complex subunit 1-like protein 1
VTDB_MOUSE	404	55162	18	1	1	Vitamin D-binding protein
WDR1_MOUSE	83	67049	5	1	1	WD repeat-containing protein 1
WFS1_MOUSE	68	101597	6	3	3	Wolframin
WN10A_MOUSE	33	47793	4	1	1	Protein Wnt-10a
XIRP1_MOUSE	35	124096	2	1	1	Xin actin-binding repeat-containing protein 1
XPO5_MOUSE	75	139050	23	1	1	Exportin-5

XXLT1_MOUSE	119	44266	19	1	1	Xyloside xylosyltransferase 1
YBOX3_MOUSE	449	38790	23	4	4	Y-box-binding protein 3
ZFY26_MOUSE	24	287343	2	1	1	Zinc finger FYVE domain-containing protein 26
ZFY27_MOUSE	250	47483	36	1	1	Protrudin
ZN692_MOUSE	31	60228	3	1	1	Zinc finger protein 692
